# Molecular signatures and phylogenomic analysis of the genus *Burkholderia*: proposal for division of this genus into the emended genus *Burkholderia* containing pathogenic organisms and a new genus *Paraburkholderia* gen. nov. harboring environmental species

**DOI:** 10.3389/fgene.2014.00429

**Published:** 2014-12-19

**Authors:** Amandeep Sawana, Mobolaji Adeolu, Radhey S. Gupta

**Affiliations:** Department of Biochemistry and Biomedical Sciences, Health Sciences Center, McMaster UniversityHamilton, ON, Canada

**Keywords:** Burkholderia, *Burkholderia cepacia* complex, conserved signature indels, phylogenetic trees, molecular signatures

## Abstract

The genus *Burkholderia* contains large number of diverse species which include many clinically important organisms, phytopathogens, as well as environmental species. However, currently, there is a paucity of biochemical or molecular characteristics which can reliably distinguish different groups of *Burkholderia* species. We report here the results of detailed phylogenetic and comparative genomic analyses of 45 sequenced species of the genus *Burkholderia*. In phylogenetic trees based upon concatenated sequences for 21 conserved proteins as well as 16S rRNA gene sequence based trees, members of the genus *Burkholderia* grouped into two major clades. Within these main clades a number of smaller clades including those corresponding to the clinically important *Burkholderia cepacia* complex (BCC) and the *Burkholderia pseudomallei* groups were also clearly distinguished. Our comparative analysis of protein sequences from *Burkholderia* spp. has identified 42 highly specific molecular markers in the form of conserved sequence indels (CSIs) that are uniquely found in a number of well-defined groups of *Burkholderia* spp. Six of these CSIs are specific for a group of *Burkholderia* spp. (referred to as Clade I in this work) which contains all clinically relevant members of the genus (viz. the BCC and the *B. pseudomallei* group) as well as the phytopathogenic *Burkholderia* spp. The second main clade (Clade II), which is composed of environmental *Burkholderia* species, is also distinguished by 2 identified CSIs that are specific for this group. Additionally, our work has also identified multiple CSIs that serve to clearly demarcate a number of smaller groups of *Burkholderia* spp. including 3 CSIs that are specific for the *B. cepacia* complex, 4 CSIs that are uniquely found in the *B. pseudomallei* group, 5 CSIs that are specific for the phytopathogenic *Burkholderia* spp. and 22 other CSI that distinguish two groups within Clade II. The described molecular markers provide highly specific means for the demarcation of different groups of *Burkholderia* spp. and they also offer novel and useful targets for the development of diagnostic assays for the clinically important members of the BCC or the *pseudomallei* groups. Based upon the results of phylogenetic analyses, the identified CSIs and the pathogenicity profile of *Burkholderia* species, we are proposing a division of the genus *Burkholderia* into two genera. In this new proposal, the emended genus *Burkholderia* will correspond to the Clade I and it will contain only the clinically relevant and phytopathogenic *Burkholderia* species. All other *Burkholderia* spp., which are primarily environmental, will be transferred to a new genus *Paraburkholderia* gen. nov.

## Introduction

The genus *Burkholderia* is a morphologically, metabolically, and ecologically diverse group of gram-negative bacteria (Yabuuchi et al., 1992; Coenye and Vandamme, 2003; Mahenthiralingam et al., 2005; Palleroni, 2005; Compant et al., 2008). *Burkholderia* species are ubiquitous in the environment (Coenye and Vandamme, 2003). They inhabit a wide range of ecological niches, ranging from soil to the human respiratory tract (Coenye and Vandamme, 2003). A group of 17 closely related *Burkholderia* species, the *Burkholderia cepacia* complex (BCC), are responsible for prevalent and potentially lethal pulmonary infections in immunocompromised individuals, such as individuals with cystic fibrosis (Mahenthiralingam et al., 2002, 2005; Biddick et al., 2003; Hauser et al., 2011). *Burkholderia pseudomallei*, a *Burkholderia* species related to the BCC, is the causative agent for the disease melioidosis, a potentially lethal septic infection which accounts for up to 20% of all community-acquired septicemias in some regions (White, 2003; Limmathurotsakul and Peacock, 2011). Other species related to the BCC are the causative agents of major infections in both animals (*Burkholderia mallei*) and plants (*Burkholderia glumae* and *Burkholderia gladioli*) (Whitlock et al., 2007; Nandakumar et al., 2009).

In spite of the large diversity and varied pathogenicity among the >70 members of the group, all *Burkholderia* species are currently placed within one genus (Coenye and Vandamme, 2003; Palleroni, 2005). The phylogeny and taxonomy of the genus *Burkholderia* is primarily defined on the basis of 16S rRNA sequence analysis (Yabuuchi et al., 1992; Palleroni, 2005; Yarza et al., 2008). The inferences obtained from 16S rRNA analysis have been further substantiated by other phylogenetic methods, including *recA* gene based analysis (Payne et al., 2005), *acdS* gene based analysis (Onofre-Lemus et al., 2009), DNA–DNA hybridization (Gillis et al., 1995), whole cell fatty acid analysis (Stead, 1992), multilocus sequence analysis (Tayeb et al., 2008; Spilker et al., 2009; Estrada-de los Santos et al., 2013), gene gain/loss analysis (Zhu et al., 2011), and whole genome phylogenetic analysis (Ussery et al., 2009; Segata et al., 2013). In many of these phylogenetic studies, the members of the genus *Burkholderia* can be divided into two or more distinct phylogenetic groups, with one group consisting of members of the BCC and related species (Payne et al., 2005; Tayeb et al., 2008; Yarza et al., 2008; Spilker et al., 2009; Ussery et al., 2009; Gyaneshwar et al., 2011; Vandamme and Dawyndt, 2011; Zhu et al., 2011; Estrada-de los Santos et al., 2013; Segata et al., 2013). Although there are some commonly shared features among closely related groups of *Burkholderia* species, there is no known morphological, biochemical, or molecular characteristic specific to the larger phylogenetic groups within the genus (ex. the BCC and related species).

The advent of next generation sequencing methods has led to a rapid increase in the number of genome sequences available for bacterial species (Mardis, 2008). The availability of these sequences for members of the genus *Burkholderia* provides us better means to evaluate the phylogenetic relationships among different species (Ciccarelli et al., 2006; Wu et al., 2009). Importantly, the large data sets of sequences allows for the use of comparative genomic techniques to discover novel molecular markers that can provide independent evidence for different phylogenetic groups within the genus *Burkholderia* (Gupta, 1998, 2014; Gao and Gupta, 2012). In this work, we describe one type of molecular marker, conserved sequence insertions or deletions (CSIs), which are uniquely present in protein sequences from a defined group of organisms, that can be used to delineate different phylogenetic groups of *Burkholderia* species independently of traditional phylogenetic methods (Gupta, 1998, 2001; Gao and Gupta, 2012). Our comparative analysis of *Burkholderia* genomes has led to the identification of 42 unique CSIs that delineate different phylogenetic groups within the genus in clear molecular terms. A clade of *Burkholderia* containing the BCC and related organisms (Clade I) was supported by both phylogenetic evidence and 6 identified CSIs. We have also identified 3 CSIs specific for the BCC, 4 CSIs specific for the *B. pseudomallei* group, and 5 CSIs specific for the plant pathogenic *Burkholderia* spp. The remaining members of the genus *Burkholderia* formed another monophyletic clade (Clade II) in our phylogenetic trees which was supported by 2 CSIs. Within Clade II, we identified two smaller clades of *Burkholderia* that were supported by 16 and 6 CSIs. The grouping of members of the genus *Burkholderia* into at least two large, monophyletic groups has also been observed in a large body of prior phylogenetic research (Payne et al., 2005; Tayeb et al., 2008; Yarza et al., 2008; Spilker et al., 2009; Ussery et al., 2009; Gyaneshwar et al., 2011; Zhu et al., 2011; Estrada-de los Santos et al., 2013; Segata et al., 2013). Based on the phylogenetic evidence and our identified CSIs, we propose division of the genus *Burkholderia* into two genera: an emended genus *Burkholderia* containing clinically important and phytopathogenic members of the genus and a new genus *Paraburkholderia* gen. nov. harboring the environmental species.

## Materials and methods

### Phylogenetic analysis

A concatenated sequence alignment of 21 highly conserved proteins (viz. ArgRS, EF-G, GyrA, GyrB, Hsp60, Hsp70, IleRS, RecA, RpoB, RpoC, SecY, ThrRS, TrpS, UvrD, ValRS, 50S ribosomal proteins L1, L5 and L6, and 30S ribosomal proteins S2, S8 and S11) was used to perform phylogenetic analysis. Due to their presence in most bacteria, these proteins have been extensively utilized for phylogenetic studies (Gupta, 1998, 2009; Kyrpides et al., 1999; Harris et al., 2003; Charlebois and Doolittle, 2004; Ciccarelli et al., 2006). The amino acid sequences for these conserved proteins were obtained from NCBI database for all of the species/strains listed in Table 1, which includes 45 sequenced species of the genus *Burkholderia*. Furthermore, three genomes from other members of class *Betaproteobacteria* (viz. *Cupriavidus necator* N-1, *Bordetella pertussis* Tohama I, and *Neisseria meningitides* MC58), serving as outgroups in our analysis, were also retrieved from NCBI database. Depending on genome availability, type strains were selected for most of the species. Multiple sequence alignments for these proteins were created using Clustal_X 1.83 and concatenated into a single alignment file (Jeanmougin et al., 1998). Poorly aligned regions from the alignment file were removed using Gblocks 0.91b and the resulting alignment, which contained 7688 aligned characters, was ultimately utilized for phylogenetic analysis (Castresana, 2000). A maximum likelihood (ML) tree based on 100 bootstrap replicates of this alignment was constructed using MEGA 6.0 while employing Jones-Taylor–Thornton substitution model (Jones et al., 1992; Tamura et al., 2013).

**Table 1:** **Genome characteristics of the sequenced members of the genus *Burkholderia***.

**Organism**	**BioProject**	**Size (Mb)**	**GC%**	**Chromosomes**	**Proteins**	**References**
*Burkholderia cenocepacia* J2315	PRJNA57953	8.06	66.9	3	7116	Holden et al., 2009
*Burkholderia pseudomallei* K96243	PRJNA57733	7.25	68.1	2	5727	Holden et al., 2004
*Burkholderia mallei* ATCC 23344	PRJNA57725	5.84	68.5	2	5022	Nierman et al., 2004
*Burkholderia thailandensis* E264	PRJNA58081	6.72	67.6	2	5632	Kim et al., 2005
*Burkholderia oklahomensis* C6786	PRJNA54789	6.99	67.0	–	6954	NMRC^b^
*Burkholderia multivorans* ATCC 17616	PRJNA58909	7.01	66.7	3	6111	DOE^d^
*Burkholderia ambifaria* AMMD	PRJNA58303	7.53	66.8	3	6610	Coenye et al., 2001b
*Burkholderia glumae* BGR1	PRJNA59397	7.28	67.9	2	5773	Lim et al., 2009
*Burkholderia xenovorans* LB400	PRJNA57823	9.73	62.6	3	8702	Chain et al., 2006
*Burkholderia sp. CCGE1002*	PRJNA42523	7.88	63.3	3	6889	Ormeno-Orrillo et al., 2012
*Burkholderia sp. CCGE1001*	PRJNA42975	6.83	63.6	2	5965	DOE^d^
*Burkholderia sp. CCGE1003*	PRJNA46253	7.04	63.2	2	5988	DOE^d^
*Burkholderia sp. Ch1-1*	PRJNA48975	8.74	62.4	–	7742	DOE^d^
*Burkholderia sp. H160*	PRJNA55101	7.89	62.9	–	7460	Ormeno-Orrillo et al., 2012
*Burkholderia sp. 383*	PRJNA58073	8.68	66.3	3	7716	DOE^d^
*Burkholderia sprentiae* WSM5005	PRJNA66661	7.76	63.2	–	–	DOE^d^
*Burkholderia sp. YI23*	PRJNA81081	8.90	63.3	3	7804	Lim et al., 2012
*Burkholderia sp. SJ98*	PRJNA160003	7.88	61.4	–	7268	Kumar et al., 2012
*Burkholderia sp. WSM2230*	PRJNA165309	6.31	63.1	–	–	DOE^d^
*Burkholderia sp. KJ006*	PRJNA165871	6.63	67.2	3	6024	Kwak et al., 2012
*Burkholderia sp. TJI49*	PRJNA179699	7.38	66.9	–	8940	Khan et al., 2013
*Burkholderia sp. BT03*	PRJNA180532	10.64	61.9	–	10126	Oak Ridge^c^
*Burkholderia sp. WSM2232*	PRJNA182741	7.21	63.1	–	–	DOE^d^
*Burkholderia sp. WSM3556*	PRJNA182743	7.68	61.8	–	–	DOE^d^
*Burkholderia sp. URHA0054*	PRJNA190816	7.24	62.8	–	–	DOE^d^
*Burkholderia sp. WSM4176*	PRJNA199219	9.07	62.9	–	8336	DOE^d^
*Burkholderia sp. JPY251*	PRJNA199221	8.61	63.1	–	7873	DOE^d^
*Burkholderia sp. JPY347*	PRJNA199222	6.39	63.1	–	5963	DOE^d^
*Burkholderia sp. RPE64*	PRJNA205541	6.96	63.1	3	6498	Shibata et al., 2013
*Burkholderia vietnamiensis* G4	PRJNA58075	8.39	65.7	3	7617	DOE^d^
*Burkholderia dolosa* AUO158	PRJNA54351	6.42	66.8	–	4795	Broad Institute^a^
*Burkholderia phymatum* STM815	PRJNA58699	8.68	62.3	2	7496	Vandamme et al., 2002b
*Burkholderia phytofirmans* PsJN	PRJNA58729	8.21	62.3	2	7241	Weilharter et al., 2011
*Burkholderia ubonensis* Bu	PRJNA54793	6.93	67.3	–	7181	NMRC^b^
*Burkholderia graminis* C4D1M	PRJNA54887	7.48	62.9	–	6747	DOE^d^
*Burkholderia rhizoxinica* HKI 454	PRJNA60487	3.75	60.7	1	3870	Lackner et al., 2011
*Burkholderia gladioli* BSR3	PRJNA66301	9.05	67.4	2	7411	Seo et al., 2011
*Burkholderia cepacia* GG4	PRJNA173858	6.47	66.7	2	5825	Hong et al., 2012
*Candidatus* Burkholderia kirkii UZHbot1	PRJNA74017	4.01	62.9	–	2069	Van Oevelen et al., 2002b
*Burkholderia mimosarum* LMG 23256	PRJNA163559	8.41	63.9	–	–	DOE^d^
*Burkholderia terrae* BS001	PRJNA168186	11.29	61.8	–	10234	Nazir et al., 2012
*Burkholderia pyrrocinia* CH-67	PRJNA199595	8.05	67.4	–	7324	Song et al., 2012
*Burkholderia kururiensis* M130	PRJNA199910	7.13	65.0	–	6311	Coutinho et al., 2013
*Burkholderia phenoliruptrix* BR3459a	PRJNA176370	7.65	63.1	2	6496	Oliveira Cunha et al., 2012
*Burkholderia bryophila* 376MFSha3.1	PRJNA201182	7.38	61.9	–	6722	DOE^d^

a *The Broad Institute Genome Sequencing Platform (Broad Institute)*.

b *Naval Medical Research Center/ Biological Defense Research Directorate (NMRC)*.

c *Oak Ridge National Lab (Oak Ridge)*.

d *DOE Joint Genome Institute (DOE)*.

A maximum likelihood 16S rRNA gene sequence consensus tree was also created for 101 sequences, which included 97 representative strains from the genus *Burkholderia* and four outgroup sequences from the genera *Cupriadivus* and *Ralstonia*. The sequences utilized in the study were obtained from the Ribosomal Database Project (RDP III) (Cole et al., 2009) and NCBI. All the sequences were aligned using MAAFT 7 (Katoh and Standley, 2013) and a ML tree based upon 1000 bootstrap replicates of this alignment was constructed using the General Time Reversible Model (Tavaré, 1986) in MEGA 6.0 (Tamura et al., 2013).

### Identification of molecular markers (CSIs)

BLASTp searches were conducted for all proteins from chromosomes 2 and 3 (accession numbers NC_008061 and NC_008061) of *Burkholderia cenocepacia* J2315 (Holden et al., 2009) to identify CSIs that are shared by different members of the genus *Burkholderia*. Species that appeared as top hits with high scoring homologs (*E* values < 1e^−20^) from the genus *Burkholderia* and other outgroups were selected. Multiple sequence alignments were created using the Clustal_X 1.83 (Jeanmougin et al., 1998). These alignments were visually inspected for the presence of insertions or deletions (indels) restricted to either some or all members of the genus *Burkholderia* and flanked by at least 5–6 conserved amino acid residues on both sides in the neighboring 30–40 amino acids. Indel queries that were not flanked by conserved regions were not further evaluated. The species specificity of the indel queries meeting the above criterion was further evaluated by performing BLASTp searches on short sequence segments containing the insertions or deletions, and their flanking conserved regions (60–100 amino acids long). The searches were conducted against the NCBI non-redundant (nr) database and a minimum of 250 BLAST hits were examined for the presence or absence of CSIs. The results of these analyses were evaluated as described in detail in our recent work (Gupta, 2014). Signature files for the CSIs that were specific for members of the genus *Burkholderia* were created and formatted using the programs SIG_CREATE and SIG_STYLE (accessible from Gleans.net) as described by Gupta (2014). The sequence alignment files presented here contain information for all detected insertions or deletions from the *Burkholderia* group of interest, but only a limited number from species that are serving as outgroups. Sequence information for different strains of various species is not shown, but they all exhibited similar pattern. Lastly, unless otherwise indicated, the CSIs shown here are specifically found in the indicated groups and similar CSIs were not detected in the 250 Blast hits with the query sequences.

## Results

### Branching pattern of *Burkholderia* species in concatenated protein and 16S rRNA trees

**Figure 1:**
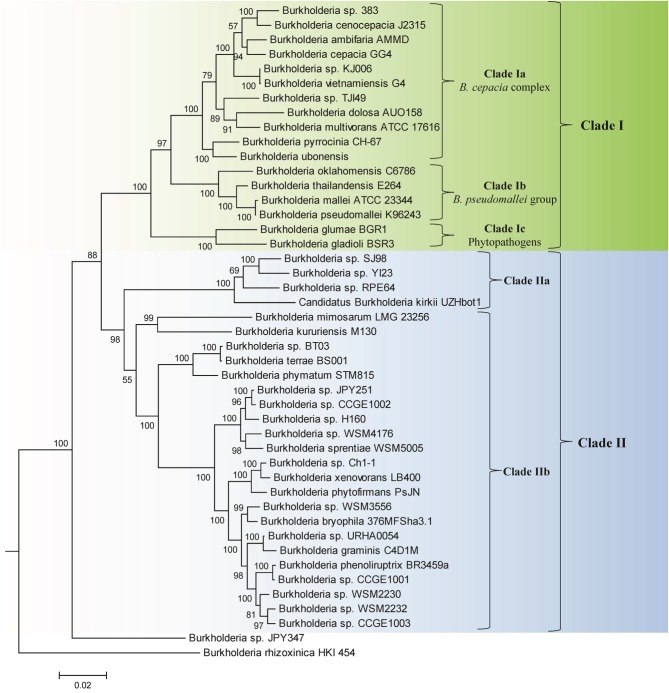
**A maximum likelihood phylogenetic tree of the genome sequenced members of the genus *Burkholderia* based upon concatenated sequences of 21 conserved proteins**. The tree was rooted using *Cupriavidus necator* N-1, *Bordetella pertussis* Tohama I, and *Neisseria meningitides* MC58. Bootstrap analysis scores are indicated for each node. The major *Burkholderia* clades (Clades I and II) and their main sub-clades are indicated by brackets.

Genome sequences of 45 species of *Burkholderia* were available from the NCBI genome database at the time of this work (NCBI, 2014). Some characteristics of these genomes are listed in Table 1. The genome sizes of the sequenced *Burkholderia* species show large variation (from 3.75–11.29 Mb) and the numbers of proteins in them also varied in a similar proportion. In this work we have produced a ML phylogenetic tree based on the concatenated amino acid sequences of 21 conserved housekeeping and ribosomal proteins obtained from 45 sequenced *Burkholderia* species (Figure 1). The *Burkholderia* species formed two large clades in the protein based ML tree: One consisting of the BCC and related organisms (Clade I) and another comprised mainly of environmental or poorly characterized *Burkholderia* species (Clade II). Within Clade I, three smaller, distinct clades are also observed. The first of these clades (Clade Ia) is wholly comprised of the sequenced BCC species, the second clade (Clade Ib) groups *B. pseudomallei* and closely related species, and the third clade (Clade Ic) consists of the plant pathogenic species, *B. glumae* and *B. gladioli*. Clade II could also be divided into two smaller clades, Clade IIa and Clade IIb. Clade IIa is separated from Clade IIb by a long branch, suggesting that a large amount of genetic divergence has occurred between the two groups. In addition to the two main clades of *Burkholderia*, two species, *Burkholderia* sp. JPY347 and *Burkholderia rhizoxinica*, branched early in the tree and did not associate with either Clade I or II.

**Figure 2:**
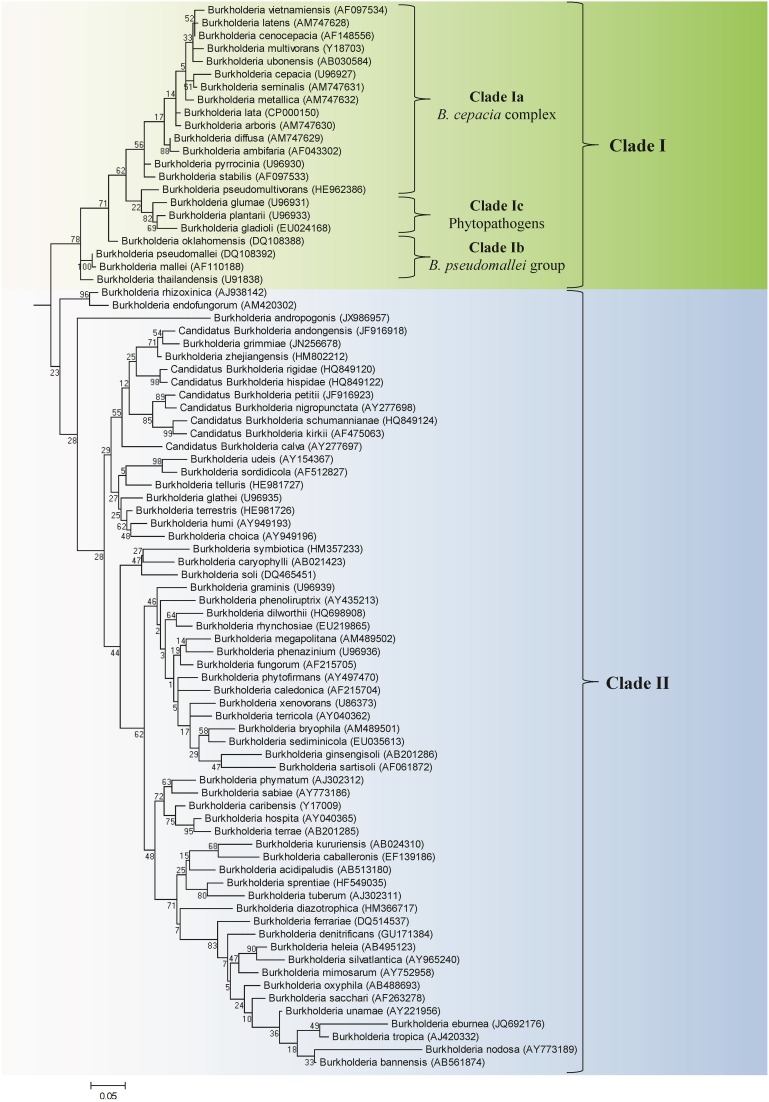
**A maximum likelihood tree based on the 16S rRNA gene sequences of 97 members of the genus *Burkholderia***. Accession numbers for the 16S rRNA sequenced used for each organism are provided in the brackets following the name of the organism. The tree was rooted using four species from the genera *Cupriadivus* and *Ralstonia*. Bootstrap analysis scores are indicated for each node. The major *Burkholderia* clades (Clades I and II) and the subclades within Clade I are indicated by brackets.

We have also constructed a 16S rRNA based ML phylogenetic tree for 97 *Burkholderia* strains and candidate species (Figure 2). In this 16S rRNA based phylogenetic tree we observed broadly similar patterns to our protein based phylogeny. A clade consisting of the BCC and related organisms (Clade I) was clearly resolved. The three subclades within Clade I, the BCC (Clade Ia), the *B. pseudomallei* group (Clade Ib), and the plant pathogenic species (Clade Ic) were well resolved, though some species exhibited aberrant branching (ex. *B. oklahomensis* and *B. pseudomultivorans*). A large assemblage of the remaining *Burkholderia* species, roughly corresponding to Clade II in our concatenated protein based phylogenetic tree, was also observed in the 16S rRNA tree. However, due to significant number of unsequenced *Burkholderia* species which are present in the 16S rRNA database it is difficult to accurately identify the groups within Clade II of the 16S rRNA tree which correspond to Clades IIa and IIb in our concatenated protein based phylogenetic tree. Bootstrap support for branches in the 16S rRNA based tree were also significantly lower than they were in the concatenated protein tree indicating that some of the observed branching patterns may not be reliable. However, the clade consisting of the BCC and related organisms (Clade I) has strong bootstrap support and has been identified in a large number of previous 16S rRNA based phylogenetic studies (Yabuuchi et al., 1992; Palleroni, 2005; Yarza et al., 2008; Suarez-Moreno et al., 2012).

### Molecular signatures distinguishing the clade I and clade II *Burkholderia*

Rare genetic changes, such as insertions and deletions in essential genes/proteins, which occur in a common ancestor can be inherited by the various decedent species related to this common ancestor (Gupta, 1998; Rokas and Holland, 2000; Gogarten et al., 2002; Gupta and Griffiths, 2002). Due to the rarity and the specific presence of these rare genetic changes to a related group of organisms, they can serve as important molecular markers and provide a novel means to understand the evolutionary interrelationships between different closely related species (Gupta, 1998; Gupta and Griffiths, 2002; Gao and Gupta, 2012).

**Figure 3:**
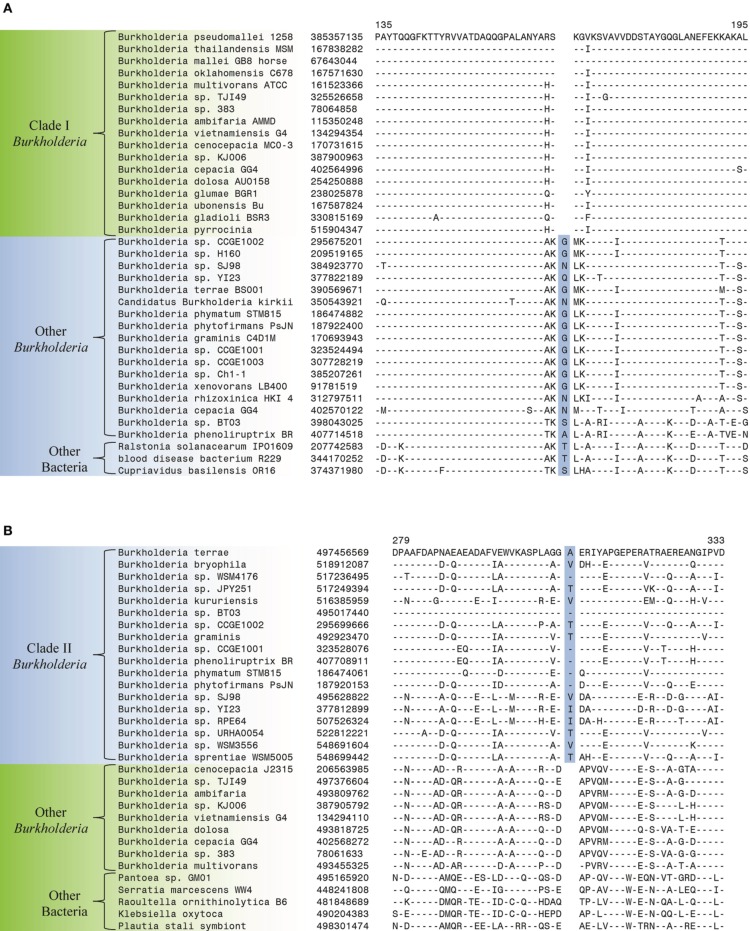
**Partial sequence alignments of (A) a periplasmic amino acid-binding protein showing a 1 amino acid deletion identified in all members of Clade I of the genus *Burkholderia* (B) a dehydrogenase showing a 1 amino acid insertion (boxed) identified only in members of Clade II of the genus *Burkholderia***. These CSIs were not found in the sequence homologs of these proteins from any other sequenced bacteria. In each case, sequence information for a *Burkholderia* species and a limited number other bacteria are shown, but unless otherwise indicated, similar CSIs were detected in all members of the indicated group and not detected in any other bacterial species in the top 250 BLAST hits. The dashes (–) in the alignments indicate identity with the residue in the top sequence. GenBank identification (GI) numbers for each sequence are indicated in the second column. Sequence information for other CSIs specific to the members of Clade I and Clade II of the genus *Burkholderia* are presented in Supplemental Figures 1–5 and Supplemental Figure 6, respectively, and their characteristics are summarized in Table 2.

The comparative analysis of protein sequences from *Burkholderia* species that was carried out in the present work has identified a number of CSIs that serve to clearly distinguish a number of different clades within the genus *Burkholderia*. These studies have led to identification of 6 CSIs that are specific for the Clade I *Burkholderia*, consisting of the BCC and related organisms, enabling clear distinction of this group from all other *Burkholderia*. This clade, which contains all well characterized pathogens within the genus, represents the most clinically relevant group within the *Burkholderia*. All species within this clade are potentially pathogenic to human, animals, or plants and most have been isolated from clinical human samples (Simpson et al., 1994; Mahenthiralingam et al., 2002, 2005; Biddick et al., 2003; O'Carroll et al., 2003). One example of a CSI that is specific to the Clade I *Burkholderia* is shown in Figure 3A. In this case, a one amino acid deletion is present in a highly conserved region of a periplasmic amino acid-binding protein. The indel is flanked on both sides by highly conserved regions indicating that it is not the result of alignment artifacts and that it is a reliable genetic characteristic. This CSI is present in all of the sequenced members of the Clade I *Burkholderia*, but absent in all other bacterial homologs of this protein. Our work has identified 5 additional CSIs in other widely distributed proteins that are specific for the Clade I *Burkholderia* and sequence alignments for these CSIs are shown in Supplemental Figures 1–5 and a summary of their characteristics is provided in Table 2.

**Table 2:** **Conserved signature indels specific for the two major clades within the genus *Burkholderia***.

**Protein Name**	**GI Number**	**Figures**	**Indel size**	**Indel position^a^**	**Specificity**
Periplasmic amino acid-binding protein	385357135	Figure 3A	1 aa del	135–195	Clade I
Putative lyase	167724527	Supplemental Figure 1	1 aa del	70–121	Clade I
4-hydroxybenzoate 3-monooxygenase	238023559	Supplemental Figure 2	1 aa ins	101–171	Clade I
6-phosphogluconate dehydrogenase, decarboxylating	330820932	Supplemental Figure 3	1 aa ins	137–202	Clade I
Putative lipoprotein	121598811	Supplemental Figure 4	1 aa del	363–393	Clade I
Sarcosine oxidase subunit alpha	493818877	Supplemental Figure 5	3 aa ins	904–965	Clade I
Dehydrogenase	497456569	Figure 3B	1 aa ins	279–333	Clade II
LysR family transcriptional regulator	187919777	Supplemental Figure 6	2 aa del	260–294	Clade II

a *The region of the specified protein that contains the indel*.

Two additional CSIs identified in this work are specific for the Clade II *Burkholderia* species which is made up of mainly environmental organisms. One of these CSIs, shown in Figure 3B, consists of a one amino acid insertion in a dehydrogenase protein that is uniquely found in members of the Clade II *Burkholderia* and absent in all other *Burkholderia* species as well all other bacterial groups. A sequence alignment for another CSI that is specific for the Clade II *Burkholderia* (a 2 aa deletion in a LysR family of transcription regulator protein) is shown in Supplemental Figure 6 and its characteristics are summarized in Table 2.

### CSIs distinguishing different main groups within the clade I *Burkholderia*

**Figure 4:**
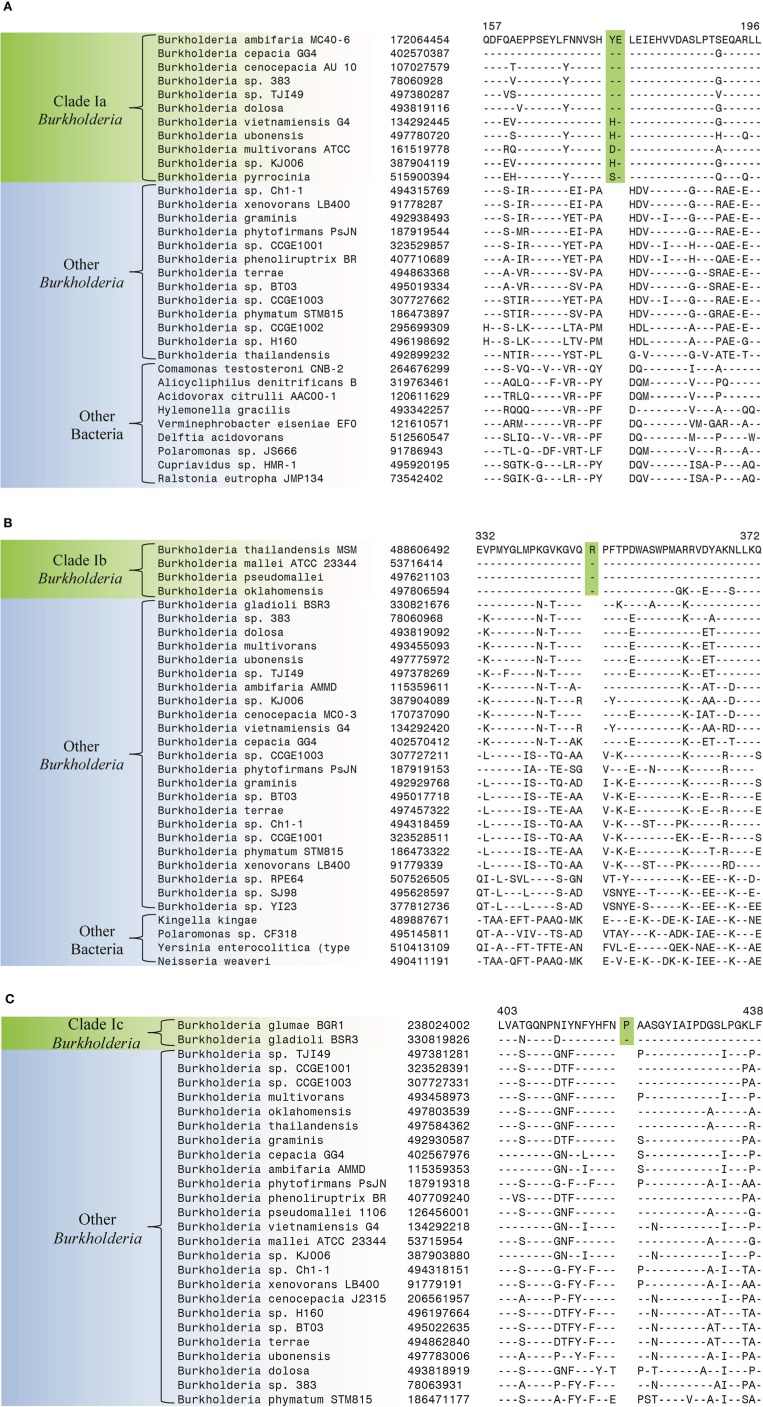
**Partial sequence alignments of (A) a histidine utilization repressor showing a 2 amino acid insertion (boxed) identified in all members of the *Burkholderia cepacia* complex (Clade Ia) within the genus *Burkholderia* (B) a periplasmic oligopeptide-binding protein showing a 1 amino acid insertion (boxed) identified in all members of the *Burkholderia pseudomallei* group (Clade Ib) within the genus *Burkholderia* (C) a SMP-30/gluconolaconase/LRE-like region-containing protein showing a 1 amino acid insertion (boxed) identified in all members of the phytopathogenic *Burkholderia* clade (Clade Ic)**. These CSIs were not found in the sequence homologs of these proteins from any other sequenced bacteria in the top 250 BLAST hits. Sequence information for other CSIs specific to subclades within Clade I of the genus *Burkholderia* are presented in Supplemental Figures 7–15 and their characteristics are summarized in Table 3.

The species within Clade I of the genus *Burkholderia* are responsible for a range of human, animal, and plant diseases (Biddick et al., 2003; Mahenthiralingam et al., 2005). The members of Clade I (i.e., the BCC and related *Burkholderia*) are commonly separated into 3 main groups which correspond to clades identified in our phylogenetic trees. The first group, the members of the BCC (Clade 1a), are prevalent pathogens in cystic fibrosis patients, the second group, the *B. pseudomallei* group (Clade Ib), contains the causative agents of melioidosis and glanders, while the third group contains the plant pathogenic *Burkholderia* species (Clade Ic) (White, 2003; Mahenthiralingam et al., 2005; Whitlock et al., 2007; Nandakumar et al., 2009). Our analysis has identified 3 CSIs that are specific for all members of the BCC clade (Clade 1a). One example of a BCC clade specific CSI is shown in Figure 4A. This CSI consists of a 2 amino acid insertion in a conserved region of a histidine utilization repressor which is only found in members of the BCC. Sequence alignments for two other BCC clade specific CSIs are shown in Supplemental Figures 7, 8 and their characteristics are summarized in Table 3.

**Table 3:** **Conserved signature indels specific for groups within Clades I and II**.

**Protein Name**	**GI Number**	**Figures**	**Indel size**	**Indel position^a^**	**Specificity**
Histidine utilization repressor	172064454	Figure 4A	2 aa ins	157–196	Clade Ia
Molybdate ABC transporter substrate-binding protein	189352411	Supplemental Figure 7	1 aa ins	110–158	Clade Ia
Acid phosphatase	221203041	Supplemental Figure 8	1 aa ins	305–338	Clade Ia
Periplasmic oligopeptide-binding protein	488606492	Figure 4B	1 aa ins	332–372	Clade Ib
OpgC protein	53716883	Supplemental Figure 9	1 aa ins	137–204	Clade Ib
Polysaccharide deacetylase family protein	167725414	Supplemental Figure 10	1 aa ins	29–63	Clade Ib
Thioredoxin domain protein	497613277	Supplemental Figure 11	1 aa ins	247–294	Clade Ib
SMP-30/gluconolaconase/LRE-like region-containing protein	238024002	Figure 4C	1 aa ins	403–438	Clade Ic
Cation efflux protein	330820376	Supplemental Figure 12	1 aa ins	129–160	Clade Ic
putative peptidoglycan-binding LysM/M23B peptidase	238024763	Supplemental Figure 13	1 aa ins	155–198	Clade Ic
SMP-30/gluconolaconase/LRE-like region-containing protein	238024002	Supplemental Figure 14	2 aa del	80–130	Clade Ic
hypothetical protein bgla_2g22890	330821370	Supplemental Figure 15	1 aa ins	322–358	Clade Ic
3-phosphoglycerate dehydrogenase	494056927	Figure 5A	1 aa ins	61–100	Clade IIa
Hypothetical protein BYI23_A021470	377821591	Supplemental Figure 16	1 aa del	16–76	Clade IIa
Prepilin peptidase	377821714	Supplemental Figure 17	1 aa ins	179–230	Clade IIa
Uracil-DNA glycosylase	495619839	Supplemental Figure 18	2 aa ins	191–230	Clade IIa
Hypothetical protein BYI23_A015260	377820970	Supplemental Figure 19	2 aa ins	221–270	Clade IIa
Carboxylate-amine ligase	377822128	Supplemental Figure 20	1 aa del	321–362	Clade IIa
NADH:ubiquinone oxidoreductase subunit M	494056355	Supplemental Figure 21	3 aa ins	303–348	Clade IIa
NADH:ubiquinone oxidoreductase subunit L	494056354	Supplemental Figure 22	1 aa ins	538–585	Clade IIa
ABC transporter	377821271	Supplemental Figure 23	1 aa del	59–99	Clade IIa
Hypothetical protein BYI23_A002220	377819666	Supplemental Figure 24	2 aa ins	133–172	Clade IIa
16S rRNA-processing protein RimM	494056031	Supplemental Figure 25	1 aa ins	147–201	Clade IIa
FAD linked oxidase domain-containing protein	377819737	Supplemental Figure 26	1 aa ins	106–144	Clade IIa
Preprotein translocase subunit SecD	495626933	Supplemental Figure 27	1 aa del	306–341	Clade IIa
Mechanosensitive ion channel protein MscS	494057445	Supplemental Figure 28	3 aa ins	101–143	Clade IIa
Hypothetical protein BYI23_A006130	377820057	Supplemental Figure 29	1 aa ins	199–253	Clade IIa
Uroporphyrinogen-III synthase	494056428	Supplemental Figure 30	7 aa ins	37–79	Clade IIa
4-hydroxyacetophenone monooxygenase	496202984	Figure 5B	1 aa ins	380–449	Clade IIb
Transposase A-like protein	187923943	Supplemental Figure 31	1 aa ins	5–50	Clade IIb
Group 1 glycosyl transferase	186475830	Supplemental Figure 32	1 aa ins	153–194	Clade IIb
4-hydroxyacetophenone monooxygenase	496202984	Supplemental Figure 33	3 aa ins	145–219	Clade IIb
Undecaprenyl-phosphate glucose phosphotransferase	209521823	Supplemental Figure 34	1 aa ins	208–275	Clade IIb
putative flavin-binding monooxygenase-like protein	186476032	Supplemental Figure 35	3 aa ins	102–148	Clade IIb

a *The region of the specified protein that contains the indel*.

Our work has also identified 4 CSIs that are specific for the *B. pseudomallei* group (Clade Ib) which contains the most prevalent human pathogen within the genus, *B. pseudomallei* (Wiersinga et al., 2006). One example of a CSI specific to the *B. pseudomallei* group, which consists of a 1 amino acid insertion in a conserved region of a periplasmic oligopeptide-binding protein, is shown in Figure 4B. Sequence alignments for three other CSIs in three different proteins that are specific for the *B. pseudomallei* group are shown in Supplemental Figures 9–11 and their characteristics are summarized in Table 3.

We have also identified 5 CSIs that are specific for the major plant pathogenic group within the genus *Burkholderia* (Clade 1c) which contains the species *B. glumae* and *B. gladioli*. An example of a CSI representing this group is shown in Figure 4C. This CSI consists of a 1 amino acid insertion in a conserved region of a SMP-30/gluconolaconase/LRE-like region-containing protein that is found in the members of Clade 1c of the genus *Burkholderia* but absent in all other *Burkholderia* and all other bacterial groups. Sequence alignments for the other 4 CSIs are shown in Supplemental Figures 12–15 and their key features are highlighted in Table 3.

### CSIs that are specific for two groups within the clade II *Burkholderia*

**Figure 5:**
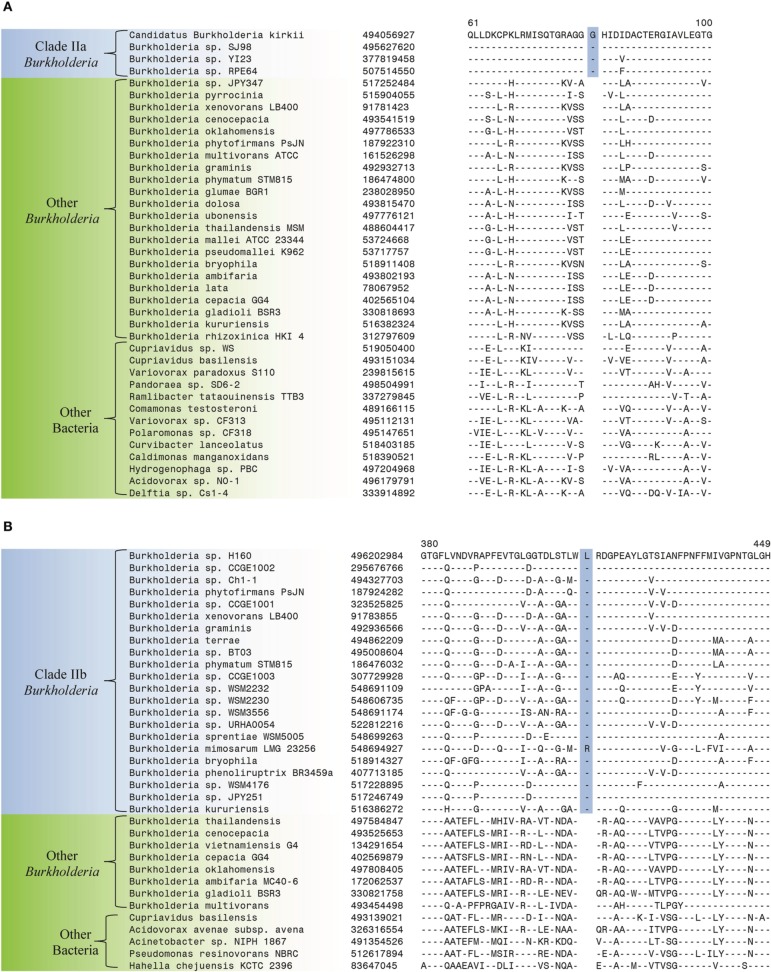
**Partial sequence alignments of (A) 3-phosphoglycerate dehydrogenase showing a 1 amino acid insertion (boxed) identified in all members of Clade IIa of the genus *Burkholderia* (B) 4-hydroxyacetophenone monooxygenase showing a 1 amino acid insertion (boxed) identified only in members of Clade IIb of the genus *Burkholderia***. These CSIs were not found in the sequence homologs of these proteins from any other sequenced bacteria in the top 250 BLAST hits. Sequence information for other CSIs specific to subclades within Clade II of the genus *Burkholderia* are presented in Supplemental Figures 16–35 and their characteristics are summarized in Table 3.

The species within Clade II of the genus *Burkholderia* inhabit a variety of environmental niches, but there is little evidence of their colonization of healthy or immunocompromised human patients (Coenye and Vandamme, 2003). The branching of different groups within Clade II is not well resolved in 16S rRNA trees and there is currently a lack of sequence data that can be used to generate trees based on concatenated gene sets that reliably resolve the interrelationships of the clade while sufficiently reflecting the total diversity of species within the clade (Figures 1, 2) (Cole et al., 2009; NCBI, 2014). Despite the limited sequence data, we have been able to identify two robust groups within Clade II that are supported by a number of CSIs. The first Clade, Clade IIa, primarily consists of unclassified members of the genus and candidatus *Burkholderia* species (Figure 1). Clade IIa is supported by 16 CSIs identified in this work. One example of a CSI specific for Clade IIa, consisting of a 1 amino acid insertion in 3-phosphoglycerate dehydrogenase, is shown in Figure 5A. This insertion is present in a highly conserved region of this protein in all sequenced members of Clade IIa and absent in all other *Burkholderia* and all other bacterial groups. Sequence alignments for the other 15 CSIs that are specific for Clade IIa *Burkholderia* spp. are shown in Supplemental Figures 16–30 and their characteristics are summarized in Table 3.

The second group within Clade II of the *Burkholderia* (Clade IIb), is comprised of a large variety of environmental *Burkholderia* species (Coenye and Vandamme, 2003; Suarez-Moreno et al., 2012). Our analysis has identified 6 CSIs that are specific to this large group of *Burkholderia* species. One example of a CSI specific to the members of Clade IIb of the genus *Burkholderia* is shown in Figure 5B. The CSI consists of a one amino acid insertion in 4-hydroxyacetophenone monooxygenase, which is only present in members of Clade IIb of the genus *Burkholderia* and not in protein homologs from any other sequenced bacterial group. Information for other 5 CSIs which are specific to members of Clade IIb of the genus *Burkholderia* are shown in Supplemental Figures 31–35 and their characteristics are summarized in Table 3.

## Discussion

The genus *Burkholderia* is one of the largest groups of species within the class *Betaproteobacteria* (Palleroni, 2005; Parte, 2013). The genus contains a variety of bacteria that inhabit a wide range of ecological niches including a number of bacteria that have pathogenic potential (Yabuuchi et al., 1992; Coenye and Vandamme, 2003; Mahenthiralingam et al., 2005; Palleroni, 2005; Compant et al., 2008). The phylogeny of the genus *Burkholderia* has been studied using a wide array of methodologies based on phenotypic, biochemical, genetic, and genomic characteristics (Stead, 1992; Gillis et al., 1995; Payne et al., 2005; Tayeb et al., 2008; Onofre-Lemus et al., 2009; Spilker et al., 2009; Ussery et al., 2009; Gyaneshwar et al., 2011; Vandamme and Dawyndt, 2011; Zhu et al., 2011; Estrada-de los Santos et al., 2013). These studies have provided novel insights into the evolutionary relationship of the species within the genus *Burkholderia*. However, no taxonomic changes have been made to date due to a lack of discrete, distinguishing characteristics identified for the different phylogenetic lineages within the genus (Estrada-de los Santos et al., 2013).

**Figure 6:**
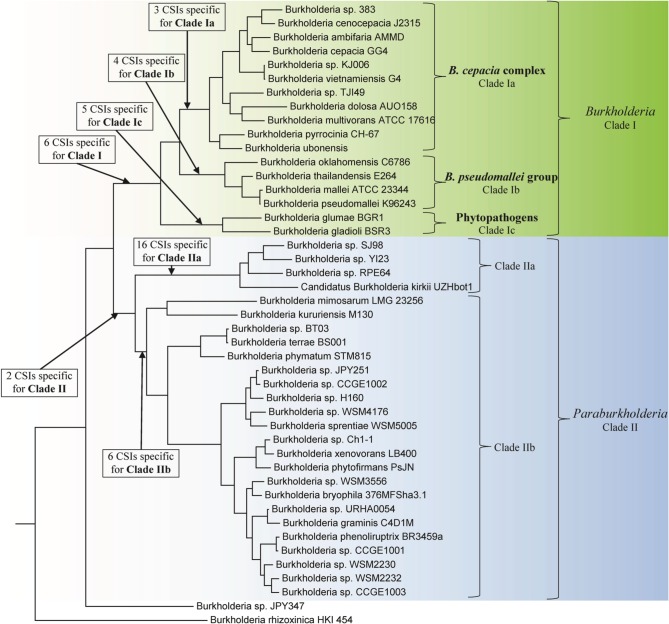
**A summary diagram depicting the distribution of identified CSIs and the proposed names of the two major groups (Clade I and II) within *Burkholderia***. The major *Burkholderia* clades are indicated by brackets and highlighting.

In the present work, we have outlined two major groups of species within the genus *Burkholderia*: Clade I, which contains all pathogenic members of the genus, and Clade II, which contains a large variety of environmental species. These two groups were found to branch distinctly in a highly resolved phylogenetic tree based on a large number of concatenated protein sequences produced in this work (Figure 1). Evidence for the distinctness of Clade I organisms from other *Burkholderia* species has been observed in a wide range of previous phylogenetic studies (Payne et al., 2005; Tayeb et al., 2008; Yarza et al., 2008; Spilker et al., 2009; Ussery et al., 2009; Gyaneshwar et al., 2011; Vandamme and Dawyndt, 2011; Zhu et al., 2011; Suarez-Moreno et al., 2012; Estrada-de los Santos et al., 2013; Segata et al., 2013). Importantly, we have also identified 6 and 2 CSIs that serve as discrete molecular characteristics of Clade I and Clade II, respectively (Figure 6 and Table 2). These CSIs are the first discrete features that have been identified that are unique to either Clade I or Clade II of the genus *Burkholderia*. These CSIs act as independent verification of the phylogenetic trends identified in this and other studies and provide clear evidence that the species from the Clade I are distinct from all other *Burkholderia* and that they are derived from a common ancestor exclusive of all other *Burkholderia*. Although sequence information for Clade II members is at present somewhat limited, based upon the shared presence of two CSIs by them, it is likely that they are also derived from a common ancestor exclusive of other bacteria.

Additionally, we have identified molecular evidence, in the form of large numbers of CSIs, which support the distinctiveness of several smaller groups within the genus *Burkholderia*. The most important of these groups, the *B. cepacia* complex (BCC; Clade Ia) and the *B. pseudomallei* group (Clade Ib), are supported by the 3 and 4 of the identified CSIs, respectively. The BCC are a group of opportunistic pathogens which colonize immunodificient human hosts and are among the most prevalent and lethal infections in cystic fibrosis patients (Mahenthiralingam et al., 2002, 2005; Biddick et al., 2003; Hauser et al., 2011). The 17 species that make up the BCC are closely related and form a tight monophyletic cluster within the genus *Burkholderia* (Vandamme and Dawyndt, 2011). The *B. pseudomallei* group consists of 4 closely related species: *B. pseudomallei*, the causative agent of the highly lethal septicemia melioidosis (White, 2003; Limmathurotsakul and Peacock, 2011), *B. mallei*, the causative agent of the equine disease glanders and occasional human infections (Whitlock et al., 2007), and the largely non-pathogenic organisms, *Burkholderia thailandensis* and *Burkholderia oklahomensis* (Deshazer, 2007). The identified CSIs are highly specific characteristics of these two important pathogenic groups and they provide novel and useful targets for the development of diagnostic assays for either the BCC or the *B. pseudomallei* group (Ahmod et al., 2011; Wong et al., 2014). We have identified CSIs for three other groups within the genus *Burkholderia*: A group of plant pathogenic *Burkholderia* related to the BCC and *B. pseudomallei* group (Clade Ic), a group containing unnamed and candidate *Burkholderia* species (Clade IIa), and a group consisting of environmental *Burkholderia* (Clade IIb). We have identified 6, 16, and 6 CSIs for these three groups, respectively. These CSIs provide important differentiating characteristics for these groups, particularly for Clades IIa and IIb which are related groups that have no other identified differentiating characteristics (Suarez-Moreno et al., 2012).

The phylogenetic analyses, identified CSIs, and the pathogenic characteristics of the different *Burkholderia* species presented in this work strongly suggest that the genus *Burkholderia* is made up of at least two distinct lineages. One lineage consisting of the BCC and related organisms (Clade I) and another consisting of a wide range of environmental organisms (Clade II). This latter clade is phylogenetically highly diverse and there is a paucity of sequence information available for its members. Thus, it is possible that in future this latter clade may be found to consist of more than one distinct bacterial lineage, however, it is currently clear that Clade I and Clade II represent distinct lineages. Evidence for the distinctness of the Clade I members from other *Burkholderia* species has been identified in a number of previous phylogenetic studies (Payne et al., 2005; Tayeb et al., 2008; Yarza et al., 2008; Spilker et al., 2009; Ussery et al., 2009; Gyaneshwar et al., 2011; Vandamme and Dawyndt, 2011; Zhu et al., 2011; Suarez-Moreno et al., 2012; Estrada-de los Santos et al., 2013; Segata et al., 2013). Estrada-de los Santos et al. (2013) recently completed a phylogenetic analysis of the genus *Burkholderia* utilizing the multilocus sequence analysis of *atpD, gltB, lepA*, and *recA* genes in combination with the 16S rRNA gene, which provides compelling evidence for the presence of two distinct evolutionary lineages within the genus *Burkholderia*. However, these authors have refrained from formally proposing a division of the genus into two genera due to a paucity of differentiating characteristics for the two groups. Our comparative analysis of *Burkholderia* genomes has identified a set of distinctive molecular characteristics that clearly differentiate the two evolutionary lineages within the genus *Burkholderia* in addition the phylogenetic evidence. In light of the abundance of phylogenetic and molecular evidence for the presence of two distinct evolutionary lineages within the genus *Burkholderia*, and the distinct pathogenicity profiles of the members of these two groups, we are proposing that genus *Burkholderia* should be divided into two separate genera. The first of these monophyletic genera, which comprises of all the clinically relevant species and clearly distinguished from all other *Burkholderia* species, will retain the name *Burkholderia* (Clade I). For the remainder of the *Burkholderia* species (Clade II), which include a wide range of environmental species, we propose the name *Paraburkholderia* gen. nov. An emended description of the genus *Burkholderia* and a description of *Paraburkholderia* gen. nov. are provided below. Brief descriptions of the new species combinations within *Paraburkholderia* gen. nov. are presented in Table 4.

**Table 4:** **Descriptions of the new combinations in the genus *Paraburkholderia* gen. nov**.

**New Combination**	**Basonym**	**Type Strain**	**References**
*Paraburkholderia acidipaludis comb. nov*.	*Burkholderia acidipaludis*	SA33	Aizawa et al., 2010b
		NBRC 101816	
		VTCC-D6-6	
*Candidatus Paraburkholderia andongensis comb. nov*.	*Candidatus Burkholderia andongensis*	—	Lemaire et al., 2011
*Paraburkholderia andropogonis comb. nov*.	*Burkholderia andropogonis*	ATCC 23061	Gillis et al., 1995
		CCUG 32772	
		CFBP 2421	
		CIP 105771	
		DSM 9511	
		ICMP 2807	
		JCM 10487	
		LMG 2129	
		NCPPB 934	
		NRRL B-14296	
*Paraburkholderia aspalathi comb. nov*.	*Burkholderia aspalathi*	VG1C	Mavengere et al., 2014
		DSM 27239	
		LMG 27731	
*Paraburkholderia bannensis comb. nov*.	*Burkholderia bannensis*	E25	Aizawa et al., 2011
		BCC 36998	
		NBRC 103871	
*Paraburkholderia bryophila comb. nov*.	*Burkholderia bryophila*	1S18	Vandamme et al., 2007
		CCUG 52993	
		LMG 23644	
*Paraburkholderia caballeronis comb. nov*.	*Burkholderia caballeronis*	TNe-841	Martínez-Aguilar et al., 2013
		CIP 110324	
		LMG 26416	
*Paraburkholderia caledonica comb. nov*.	*Burkholderia caledonica*	W50D	Coenye et al., 2001a
		CCUG 42236	
		CIP 107098	
		JCM 21561	
		LMG 19076	
		NBRC 102488	
*Candidatus Paraburkholderia calva comb. nov*.	*Candidatus Burkholderia calva*	—	Van Oevelen et al., 2004
*Paraburkholderia caribensis comb. nov*.	*Burkholderia caribensis*	MWAP64	Achouak et al., 1999
		CCUG 42847	
		CIP 106784	
		DSM 13236	
		LMG 18531	
*Paraburkholderia caryophylli comb. nov*.	*Burkholderia caryophylli*	ATCC 25418	Yabuuchi et al., 1992
		CCUG 20834	
		CFBP 2429	
		CFBP 3818	
		CIP 105770	
		DSM 50341	
		HAMBI 2159	
		ICMP 512	
		JCM 9310	
		JCM 10488	
		LMG 2155	
		NCPPB 2151	
*Paraburkholderia choica comb. nov*.	*Burkholderia choica*	LMG 22940	Vandamme et al., 2013
		CCUG 63063	
*Paraburkholderia denitrificans comb. nov*.	*Burkholderia denitrificans*	KIS30-44	Lee et al., 2012
		DSM 24336	
		KACC 12733	
*Paraburkholderia diazotrophica comb. nov*.	*Burkholderia diazotrophica*	JPY461	Sheu et al., 2013
		NKMU-JPY461	
		BCRC 80259	
		KCTC 23308	
		LMG 26031	
*Paraburkholderia dilworthii comb. nov*.	*Burkholderia dilworthii*	WSM3556	De Meyer et al., 2014
		LMG 27173	
		HAMBI 3353	
*Paraburkholderia eburne comb. nov*.	*Burkholderia eburne*	RR11	Kang et al., 2014
		KEMC 7302-065	
		JCM 18070	
*Paraburkholderia endofungorum comb. nov*.	*Burkholderia endofungorum*	HKI 456	Partida-Martinez et al., 2007
		CIP 109454	
		DSM 19003	
*Paraburkholderia ferrariae comb. nov*.	*Burkholderia ferrariae*	FeGl01	Valverde et al., 2006
		CECT 7171	
		DSM 18251	
		LMG 23612	
*Paraburkholderia fungorum comb. nov*.	*Burkholderia fungorum*	Croize P763-2	Coenye et al., 2001a
		CCUG 31961	
		CIP 107096	
		JCM 21562	
		LMG 16225	
		NBRC 102489	
*Paraburkholderia ginsengisoli comb. nov*.	*Burkholderia ginsengisoli*	KMY03	Kim et al., 2006
		KCTC 12389	
		NBRC 100965	
*Paraburkholderia glathei comb. nov*.	*Burkholderia glathei*	ATCC 29195	Vandamme et al., 1997
		CFBP 4791	
		CIP 105421	
		DSM 50014	
		JCM 10563	
		LMG 14190	
*Paraburkholderia graminis comb. nov*.	*Burkholderia graminis*	C4D1M	Viallard et al., 1998
		ATCC 700544	
		CCUG 42231	
		CIP 106649	
		LMG 18924	
*Paraburkholderia grimmiae comb. nov*.	*Burkholderia grimmiae*	R27	Tian et al., 2013
		CGMCC 1.11013	
		DSM 25160	
*Paraburkholderia heleia comb. nov*.	*Burkholderia heleia*	SA41	Aizawa et al., 2010a
		NBRC 101817	
		VTCC-D6-7	
*Candidatus Paraburkholderia hispidae comb. nov*.	*Candidatus Burkholderia hispidae*	—	Lemaire et al., 2012
*Paraburkholderia hospita comb. nov*.	*Burkholderia hospita*	LMG 20598	Goris et al., 2002
		CCUG 43658	
*Paraburkholderia humi comb. nov*.	*Burkholderia humi*	LMG 22934	Vandamme et al., 2013
		CCUG 63059	
*Candidatus Paraburkholderia kirkii comb. nov*.	*Candidatus Burkholderia kirkii*	—	Van Oevelen et al., 2002a
*Paraburkholderia kururiensis comb. nov*.	*Burkholderia kururiensis*	KP23	Zhang et al., 2000
		ATCC 700977	
		CCUG 43663	
		CIP 106643	
		DSM 13646	
		JCM 10599	
		LMG 19447	
*Paraburkholderia megapolitana comb. nov*.	*Burkholderia megapolitana*	A3	Vandamme et al., 2007
		CCUG 53006	
		LMG 23650	
*Paraburkholderia mimosarum comb. nov*.	*Burkholderia mimosarum*	PAS44	Chen et al., 2006
		BCRC 17516	
		LMG 23256	
*Candidatus Paraburkholderia nigropunctata comb. nov*.	*Candidatus Burkholderia nigropunctata*	—	Van Oevelen et al., 2004
*Paraburkholderia nodosa comb. nov*.	*Burkholderia nodosa*	Br3437	Chen et al., 2007
		BCRC 17575	
		LMG 23741	
*Paraburkholderia oxyphila comb. nov*.	*Burkholderia oxyphila*	OX-01	Otsuka et al., 2011
		DSM 22550	
		NBRC 105797	
*Candidatus Paraurkholderia petitii comb. nov*.	*Candidatus Burkholderia petitii*	—	Lemaire et al., 2011
*Paraburkholderia phenazinium comb. nov*.	*Burkholderia phenazinium*	ATCC 33666	Viallard et al., 1998
		CCUG 20836	
		CFBP 4793	
		CIP 106502	
		DSM 10684	
		JCM 10564	
		LMG 2247	
		NCIMB 11027	
*Paraburkholderia phenoliruptrix comb. nov*.	*Burkholderia phenoliruptrix*	AC1100	Coenye et al., 2004
		CCUG 48558	
		LMG 22037	
*Paraburkholderia phymatum comb. nov*.	*Burkholderia phymatum*	STM815	Vandamme et al., 2002a
		LMG 21445	
		CCUG 47179	
*Paraburkholderia phytofirmans comb. nov*.	*Burkholderia phytofirmans*	PsJN	Sessitsch et al., 2005
		CCUG 49060	
		LMG 22146	
*Paraburkholderia rhizoxinica comb. nov*.	*Burkholderia rhizoxinica*	HKI 454	Partida-Martinez et al., 2007
		CIP 109453	
		DSM 19002	
*Paraburkholderia rhynchosiae comb. nov*.	*Burkholderia rhynchosiae*	WSM3937	De Meyer et al., 2013b
		LMG 27174	
		HAMBI 3354	
*Candidatus Paraburkholderia rigidae comb. nov*.	*Candidatus Burkholderia rigidae*	—	Lemaire et al., 2012
*Paraburkholderia sabiae comb. nov*.	*Burkholderia sabiae*	Br3407	Chen et al., 2008
		BCRC 17587	
		LMG 24235	
*Paraburkholderia sacchari comb. nov*.	*Burkholderia sacchari*	CCT 6771	Brämer et al., 2001
		CCUG 46043	
		CIP 107211	
		IPT 101	
		LMG 19450	
*Paraburkholderia sartisoli comb. nov*.	*Burkholderia sartisoli*	RP007	Vanlaere et al., 2008
		CCUG 53604	
		ICMP 13529	
		LMG 24000	
*Candidatus Paraburkholderia schumannianae comb. nov*.	*Candidatus Burkholderia schumannianae*	—	Lemaire et al., 2012
*Paraburkholderia sediminicola comb. nov*.	*Burkholderia sediminicola*	HU2-65W	Lim et al., 2008
		KCTC 22086	
		LMG 24238	
*Paraburkholderia silvatlantica comb. nov*.	*Burkholderia silvatlantica*	SRMrh-20	Perin et al., 2006
		ATCC BAA-1244	
		LMG 23149	
*Paraburkholderia soli comb. nov*.	*Burkholderia soli*	GP25-8	Yoo et al., 2007
		DSM 18235	
		KACC 11589	
*Paraburkholderia sordidicola comb. nov*.	*Burkholderia sordidicola*	CCUG 49583	Lim et al., 2003
		JCM 11778	
		KCTC 12081	
*Paraburkholderia sprentiae comb. nov*.	*Burkholderia sprentiae*	WSM5005	De Meyer et al., 2013a
		LMG 27175	
		HAMBI 3357	
*Paraburkholderia symbiotica comb. nov*.	*Burkholderia symbiotica*	JPY-345	Sheu et al., 2012
		NKMU-JPY-345	
		BCRC 80258	
		KCTC 23309	
		LMG 26032	
*Paraburkholderia telluris comb. nov*.	*Burkholderia telluris*	LMG 22936	Vandamme et al., 2013
		CCUG 63060	
*Paraburkholderia terrae comb. nov*.	*Burkholderia terrae*	KMY02	Yang et al., 2006
		KCTC 12388	
		NBRC 100964	
*Paraburkholderia terrestris comb. nov*.	*Burkholderia terrestris*	LMG 22937	Vandamme et al., 2013
		CCUG 63062	
*Paraburkholderia terricola comb. nov*.	*Burkholderia terricola*	CCUG 44527	Goris et al., 2002
		LMG 20594	
*Paraburkholderia tropica comb. nov*.	*Burkholderia tropica*	Ppe8	Reis et al., 2004
		ATCC BAA-831	
		DSM 15359	
		LMG 22274	
*Paraburkholderia tuberum comb. nov*.	*Burkholderia tuberum*	STM678	Vandamme et al., 2002a
		CCUG 47178	
		LMG 21444	
*Paraburkholderia udeis comb. nov*.	*Burkholderia udeis*	LMG 27134	Vandamme et al., 2013
		CCUG 63061	
*Paraburkholderia unamae comb. nov*.	*Burkholderia unamae*	MTl-641	Caballero-Mellado et al., 2004
		ATCC BAA-744	
		CIP 107921	
*Paraburkholderia xenovorans comb. nov*.	*Burkholderia xenovorans*	LB400	Goris et al., 2004
		CCUG 46959	
		LMG 21463	
		NRRL B-18064	
*Paraburkholderia zhejiangensis comb. nov*.	*Burkholderia zhejiangensis*	OP-1	Lu et al., 2012
		KCTC 23300	

### Emended description of the genus *Burkholderia* (Yabuuchi et al., 1993 emend. Gillis et al., 1995)

The genus contains the type species *B. cepacia* (Yabuuchi et al., 1993). The species from this genus are gram-negative, straight or slightly curved rods, which exhibit motility mediated by one or more polar flagella. Only, *B. mallei* lacks flagella and is non-motile. The species do not produce sheaths or prosthecae and do not go through any resting stages. Most species are able to accumulate and utilize poly-β-hydroxybutyrate (PHB) for growth. The species are mostly aerobic chemoorganotrophs, but some species are capable of anaerobic respiration using nitrate as the terminal electron acceptor. The G+C content for the members of the genus ranges from 65.7 to 68.5%. The members of the genus form a distinct monophyletic clade in phylogenetic trees, and they are distinguished from all other bacteria by the conserved sequence indels reported in this work in the following proteins: Periplasmic amino acid-binding protein, 4-hydroxybenzoate 3-monooxygenase, 6-phosphogluconate dehydrogenase, Sarcosine oxidase subunit alpha, a putative lipoprotein, and a putative lyase (Table 2).

### Description of the genus *paraburkholderia* gen. nov.

The genus contains the type species *Paraburkholderia graminis* comb. nov. (Basonym: *Burkholderia graminis*, Viallard et al., 1998) The species from this genus are gram-negative straight or slightly curved rods with one or more polar flagella. Other morphological and metabolic characteristics are similar to genus *Burkholderia*. The G+C content for the members of the genus ranges from 61.4 to 65.0%. The species are not associated with humans. The members of this genus generally form a distinct clade in the neighborhood of genus *Burkholderia* in phylogenetic trees, and they lack the molecular signatures which are specific for *Burkholderia*. Most of the sequenced members from this genus contain the conserved sequence indels reported in this work in the protein sequences of an unnamed dehydrogenase and a LysR family transcriptional regulator (Table 2).

## Conflict of interest statement

The authors declare that the research was conducted in the absence of any commercial or financial relationships that could be construed as a potential conflict of interest.
