# Supplementary material for: Molecular signatures and phylogenomic analysis of the genus Burkholderia: proposal for division of this genus into the emended genus Burkholderia containing pathogenic organisms and a new genus Paraburkholderia gen. nov. harboring environmental species
Source: Front Genet. 2014 Dec 19;5:429. doi: 10.3389/fgene.2014.00429 (PMC4271702; doi:10.3389/fgene.2014.00429)
Supplement: Supplementary file 1 [file DataSheet1.PDF]

Group	Species	ID	70		121
Clade I *Burkholderia*	Burkholderia pseudomallei DM98	167724527	NRFGRVGVRIHDISHPHWRDDVRVVLRAAR		PPAYLTLPKVGGAADAAEMCAF
	Burkholderia mallei ATCC 23344	53716125	------------------------------		---------------------
	Burkholderia oklahomensis C678	167573545	-------A---------------I------		---------------------
	Burkholderia thailandensis E26	83716056	-------A-L-------------II-----		---------------------
	Burkholderia multivorans ATCC	421469340	-------A----F----------II-----		---------IAN---------
	Burkholderia vietnamiensis G4	134293102	------------F----------I------		A---V-----A--------I--
	Burkholderia sp. KJ006	387904801	------------F----------I------		A---V-----A--------I--
	Burkholderia sp. TJI49	416935983	-------A----F----------II----Q		---------IAS---------
	Burkholderia cenocepacia H111	421866226	------------F----------I----S-		V---I-----AN-------T--
	Burkholderia sp. 383	78063002	------------F----------I------		A---I----IAS-------T--
	Burkholderia glumae BGR1	238024238	-----A------F--------L-I------		----I----TTD---V--V---
	Burkholderia ubonensis Bu	167583939	-------A----F----------II----Q		A---I----IAN---------
	Burkholderia gladioli BSR3	330820921	-----A------F--------L-I------		A---I-----TS---V--A---
	Burkholderia ambifaria MC40-6	172062856	------------F----------I------		A---V----IAS-------V--
	Burkholderia cepacia GG4	402569561	------------F----------I------		A---------AS-------V--
	Burkholderia dolosa AU0158	254253637	------------F----------I------		A---V----IAN-------A--
	Burkholderia pyrrocinia	515899442	-------A----F----------II---G-		---------IAN---------
Other *Burkholderia*	Burkholderia rhizoxinica HKI 4	312796845	-----------AYD---------I--H-P-	C	----IM----R-LHE---Q---
	Burkholderia phenoliruptrix BR	407710017	D-----------FA---------LI-----	Q	A---I----IRSVH-----V--
	Burkholderia sp. CCGE1001	323529174	D-----------FV---------LI-----	Q	A---I----IRSVH-----V--
	Burkholderia phymatum STM815	186473176	D-----------FN-A-------LI-----	R	A--FIM---IRSVP--------
	Burkholderia xenovorans LB400	91777238	D-----------FD-A-------LI----K	R	A---I----IRHVH-----V--
	Burkholderia sp. Ch1-1	385206389	D-----------FD-A-------LI----K	R	A---I----IRNGH-----V--
	Burkholderia sp. CCGE1003	307726619	D-----------FE---------LI-----	R	A---IA---IRTVP-----I--
	Burkholderia graminis C4D1M	170691020	D-----------YD--Y------LI----K	R	A--FI----IRNVL-----V--
	Burkholderia sp. CCGE1002	295700752	D-----------FA-T-------LI----K	R	A---I----IRNVH-----I--
	Burkholderia sp. H160	209521211	D-----------FA-A-------LI----K	R	A---I----IRNVH-----I--
	Burkholderia terrae BS001	390568312	D-----------FN-A-------LI----K	R	A--FI----IRNVP--------
	Burkholderia sp. BT03	420252306	D-----------FN-A-------LI----K	R	A--FI----IRNVP--------
	Burkholderia phytofirmans PsJN	187921490	D-----------FD-A-------LI----K	R	A---I----IRNIP-----V--
	Candidatus Burkholderia kirkii	350546108	D-----------FN-RA------II--Q-K	K	A--FIM---IRSVFE----V--
	Burkholderia sp. YI23	377813435	D-----------FH-RA------II--S-K	K	A--FIM---IRSVL-----V--
	Burkholderia sp. SJ98	413965205	D-----------FD-RA------II--R-K	K	A--FIM---IRS----T--V--
Other Bacteria	Methylibium petroleiphilum PM1	124267360	--H------V-PYP--SFA----SL-AG-G	E	RL---MI--AQ-VEELRDAI-A
	Ralstonia eutropha H16	116694307	-----A-A----P---A--Q--DIIV-E-G	S	RL--I-V--AT-SGQV--VIRY
	Cupriavidus sp. HMR-1	430805917	-----A-A----P---A--Q--EIIVGQ-G	S	RL--I-V--ATRHEQV--VI–

Supplemental Figure 1: Partial sequence alignment of a conserved region of a Putative lyase showing a 1 amino acid deletion that is uniquely shared by the Clade I species.

Group	Organism	GI	101	(boxed)	171
Clade I *Burkholderia*	Burkholderia glumae BGR1	238023559	SQHEVVRDLIAAGVAHGHEMHFEVSEVALHDVESERPSVTFRH	R	DGRAGRIDCDFIAGCDGFHGIARQTIP
	Burkholderia cenocepacia J2315	206561927	--------------E---Q------D-----------F---K-	A	----D-----Y----------------
	Burkholderia ambifaria MEX-5	171316023	-------------D----A-----TD-----------F---K-	A	----D-----Y----------------
	Burkholderia cepacia GG4	402570263	-------------D----Q------D---------H-F---K-	A	----D-----Y----------------
	Burkholderia sp. 383	78063976	--------------E---A---D--D-------G-H-F---K-	A	----D-----Y----------------
	Burkholderia vietnamiensis G4	134292456	-------------DT---Q---D--D--------D--F---T-	A	----E-----Y----------------
	Burkholderia dolosa AU0158	254254867	--------------R-R-P-----TD------D----F---K-	A	----E-----Y----------------
	Burkholderia gladioli BSR3	330819252	-------------IEQ--A-----------E-TG---A----R	Q	--V-E----------------------
	Burkholderia multivorans ATCC	161521824	--------------E---P---D-TD------DGD--F---K-	A	----D-----Y----------------
	Burkholderia sp. KJ006	387904154	-----------V-DT---Q---D--D--------D--F---T-	A	----E-----Y--------------V-
	Burkholderia sp. TJI49	416935621	--------------E---Q------G--------AH-F---T-	A	---ED-----Y----------------
	Burkholderia ubonensis Bu	167588045	--------------E---A------D------DGDK-Y-I-K-	A	----D-----Y-------------A--
	Burkholderia pseudomallei DM98	167721946	------------ALE--QPI--D-RD------AT-------M-	A	---PD-----YV--------V---A--
	Burkholderia mallei ATCC 23344	53715971	----------E-ALE--QPI--D-RD------AT-------M-	A	---PD-----YV--------V---A--
	Burkholderia thailandensis MSM	167838513	------------ALEN-QPI--D-RD----G-ATD------T-	A	---TD-----Y---------V------
	Burkholderia oklahomensis EO14	167564717	------------ALE--LPA--D-RD-E----AT-------T-	A	---TE-----YV--------T------
	Burkholderia pyrrocinia	515900267	--------------E---A------D—V----D-DH-----K-	A	----E-----Y-------------A--
Other *Burkholderia*	Burkholderia sp. BT03	420254863	G-----C-M---A-E-QQQLY-D----S---LT-DK-W-Q-T-		--AVQ-----YV--------V--ES--
	Burkholderia terrae BS001	390572078	G-----C-M---A-E-QQQLY-D----S---LT-DK-WIQ-T-		--AVQ-----YV--------V--ES--
	Burkholderia phymatum STM815	186473274	G-------M--SADE-EQPLY-G--D-T---LATDG-W-G-TL		--AMQ----E-V------------S--
	Burkholderia sp. Ch1-1	385209444	A----IK--V--R--D-GKLR-G--QTSI--I-TD---IRYH-		E-E-YELQ---VI----SQ-VS-AS--
	Burkholderia sp. H160	209520001	A----LK--V--R--A-A-LR-G--DTSV-GIDTDK--IRY--		E-AEHELQ---VI----SQ-VS--A--
	Burkholderia phytofirmans PsJN	187924211	A----IK--V--R--DDGKLR-G---TSI-GIDTDT--IRY--		E-E-YELQ---VI----SQ-VS-AS--
Other Bacteria	Ralstonia pickettii 12D	241663645	G-----K-----R-EQ-APLL-D----SV--I--AT---Q-V-		--VPQTLQ--Y----------C--A--
	Ralstonia solanacearum CMR15	299066155	G-----K-----RIGQ-APLL------S---I--AT---R-V-		E-VPQTLH--Y----------C-PA--
	blood disease bacterium R229	344167318	G-----K-----RIGQ-APLL------S---I--AS---R-V-		E-VPQTLH--Y----------C-PA--
	Ralstonia syzygii R24	344171972	G-----K-----RIGQ-APLL------S---I--AS---R-V-		E-VVQTLH--Y----------C-PA--
	Ralstonia eutropha JMP134	73537839	G-----------RQ-C-APLL----Q----GLDTQS--LS-E-		G-QRHVLA---------S--VS-HAV-
	Cupriavidus basilensis OR16	374371149	G-----K-----RL-A-APTL-Q--G----GFDGNK-----A-		G--RHTLH------------VS-EA--
	Herbaspirillum sp. CF444	399021994	A----IK--V--RL-AQGQLL----D-VPK--QT-H---S-M-		--Q-QQLQA--VI-------VS-PQ--
	Cupriavidus necator N-1	339328246	G-T--T---MD-RL-A-APTIYQAAN-R----KGAQ-Y---ER		--ETI-L---Y----------S--A—

Supplemental Figure 2: Partial sequence alignment of a conserved region of 4-hydroxybenzoate 3-monooxygenase showing a 1 amino acid insertion (boxed) that is uniquely shared by the Clade I species and *Ramlibacter tataouinensis*.

Group	Organism	ID	137	Insertion	202
Clade I *Burkholderia*	Burkholderia gladioli BSR3	330820932	GPSIMPGGQRDAYDLVEPILKQIAAKAP	G	DGEPCVAYMGPDGAGHYVKMVHNGIEYGDMQLIAESY
	Burkholderia cenocepacia AU 10	107026803	----------------------------	S	-------------------------------------
	Burkholderia ambifaria MC40-6	172062833	----------------------------	S	-------------------------------------
	Burkholderia vietnamiensis G4	134293079	----------------------------	S	-------------------------------------
	Burkholderia ubonensis Bu	167583918	----------------------------	S	-------------------------------------
	Burkholderia sp. 383	78063028	----------------------------	S	-------------------------------------
	Burkholderia multivorans CF2	421476962	----------------------------	S	-------------------------------------
	Burkholderia sp. TJI49	416955048	----------------------------	S	-------------------------------------
	Burkholderia oklahomensis C678	167573573	----------------------------	A	-------------------------------------
	Burkholderia dolosa AU0158	254253619	----------------------------	S	-------------------------------------
	Burkholderia thailandensis E26	83717630	----------------------------	A	-------------------------------------
	Burkholderia pseudomallei Past	254186165	--------------------E-------	A	-------------------------------------
	Burkholderia mallei ATCC 23344	53716584	--------------------E-------	A	-------------------------------------
	Burkholderia cepacia GG4	402569584	----------------------------	S	-D-----------------------------------
	Burkholderia sp. KJ006	387904778	----------------------------	S	-------------------------------------
	Burkholderia pyrrocinia	515899462	----------------------------	S	-------------------------------------
Other *Burkholderia*	Burkholderia glumae BGR1	238024189	----------------------------		----------------F--------------------
	Burkholderia xenovorans LB400	91777918	----------------A---TE------		----------------F--------------------
	Burkholderia sp. Ch1-1	385204185	----------------A---TE------		----------------F--------------------
	Burkholderia sp. CCGE1003	307726273	----------------A---TE------		----------------F--------------------
	Burkholderia phytofirmans PsJN	187920646	----------------A---TE------		----------------F--------------------
	Burkholderia graminis C4D1M	170692681	----------------A---TE------		----------------F--------------------
	Burkholderia sp. CCGE1002	295699075	----------------A---TE------		----------------F--------------------
	Burkholderia sp. CCGE1001	323529513	----------------A---TE------		----------------F--------------------
	Burkholderia phymatum STM815	186473528	----------E-----A---TE------		----------------F--------------------
	Burkholderia sp. BT03	420254205	----------E-----A---TE------		----------------F--------------------
	Burkholderia sp. H160	209521521	--------P-------A---TE------		----------------F--------------------
	Burkholderia terrae BS001	390575456	----------E-----A---TE------		----------A-----F--------------------
	Burkholderia sp. SJ98	413965241	---------KE--E--A---TE------		----------------F--------------------
	Burkholderia sp. YI23	377813458	---------KE--E--A---TE------		----------------F--------------------
	Candidatus Burkholderia kirkii	350545940	---------KET-E--A---TE------		----------------F--------------------
	Burkholderia rhizoxinica HKI 4	312602594	--------P----ER-----TR---R-D		-------------------------------------
Other Bacteria	Arsenophonus nasoniae	284007095	---------KQ-----A---Q--V---		------S-I--------------------------V-
	Rhodanobacter fulvus Jip2	389792522	--------P-----------TE---R-K		--K-------T--------------------------

Supplemental Figure 3: Partial sequence alignment of a conserved region of 6-phosphogluconate dehydrogenase showing a 1 amino acid insertion (boxed) that is uniquely shared by the Clade I species excluding *Burkholderia glumae*.

Group	Organism	GI	363		393
Clade I *Burkholderia*	Burkholderia mallei SAVP1	121598811	LDYTQAVPGTVTSTHVGASST		TPTGKFIFTG
	Burkholderia gladioli BSR3	330815207	---------------------		----------
	Burkholderia pseudomallei 1026	386863510	---------------------		----------
	Burkholderia thailandensis MSM	488604877	---A-----------------		----------
	Burkholderia oklahomensis	497792825	-----T----I--A------S		----------
	Burkholderia glumae BGR1	238025899	------T------V-T--T--		----------
	Burkholderia sp. 383	78064888	-----K------TI-T-----		----------
	Burkholderia sp. TJI49	497377103	-----K------TI-T----S		----------
	Burkholderia ubonensis	497778790	-----K------TV-T--G--		----------
	Burkholderia ambifaria	493810174	-----K-------I-T-SG--		S---------
	Burkholderia cepacia GG4	402565031	-----K------TI-T-SG--		S---------
	Burkholderia cenocepacia J2315	206558605	-----K------TI-T-SG--		S---------
	Burkholderia multivorans	493457356	-----K------TI-T-SG--		S---------
	Burkholderia vietnamiensis G4	134294407	-----K------TI-T-SG--		S---------
	Burkholderia dolosa	493815257	-----K------TI-T-SG--		S---------
	Burkholderia sp. KJ006	387903811	--F--Q---V--TV-AD-A-G		PA----V---
	Burkholderia pyrrocinia	515904380	-----K------TV-S-SG--		----------
Other *Burkholderia*	Burkholderia sp. Ch1-1	494322698	--F------V--T-Q----TG	T	-----M----
	Burkholderia xenovorans LB400	91785505	--F------V--T-Q----TG	T	-----M----
	Burkholderia phytofirmans PsJN	187925654	--F------V--T-K----TG	T	-----M----
	Burkholderia sp. CCGE1001	323527639	--F--T---V--T------TG	N	-----M----
	Burkholderia graminis	492940925	--F------V--T---N--TG	N	-----M----
	Burkholderia sp. CCGE1003	307731291	--F--S---V--T-R----TG	N	-----M----
	Burkholderia terrae	497455747	--F--T---V----YAN-AAG	S	A---------
	Burkholderia sp. BT03	495004789	--F--T---V----YAN-AAG	S	A---------
Other Bacteria	Ralstonia pickettii 12D	241663615	--F--S---VIRT-QTS-PAG	S	A----L----
	Ralstonia sp. 5_2_56FAA	496534466	--F--T---VIRT-QTS-PAG	S	A----L----
	Ralstonia sp. 5_7_47FAA	496576345	--F--T---VIRT-QTS-PAG	S	A----L----
	Cupriavidus necator N-1	339327765	---S-Q---V-RTSRRE-PAG	S	A----LM---
	Ralstonia eutropha JMP134	73543126	---S-T---V-RV-PRD-AAN	A	L----LM---
	Cupriavidus taiwanensis LMG 19	194291262	--FS-Q---V-RTSRRE-PAG	S	A----LM---
	Ralstonia sp. GA3-3	498510901	--FS-Q---V-RTSRRD-PAG	S	A----LM---

Supplemental Figure 4: Partial sequence alignment of a conserved region of putative lipoprotein showing a 1 amino acid deletion (unboxed) that is uniquely shared by the Clade I species and *Cupriavidus basilensis*.

Group	Species	Accession	904	(boxed insertion)	965
Clade I *Burkholderia*	Burkholderia dolosa	493818877	RKQFVGLLTDDEQFVLPEGAQIVAKDTQVS	TVD	PTPMIGHVTSSYYSPILQRSIALAVVKGG
	Burkholderia ubonensis	497775187	------------------------------	-TE	-----------------R-----------
	Burkholderia multivorans	493461290	---------E------------------I-	--E	-----------------K-----------
	Burkholderia sp. TJI49	497375641	---------E-----------I--------	-TE	-----------------K-----------
	Burkholderia pseudomallei	490687287	-----------A-S-----G---EL-AAAR	ADG	T---L------------N-----------
	Burkholderia cenocepacia	493526553	---------E-----------I--------	AT-	-----------------K-----------
	Burkholderia thailandensis	497585684	-----------A-Y-----G---EL-AAAR	ADG	T---L------------N-----------
	Burkholderia sp. 383	78061338	---------E-----------I--------	A--	-----------------K-----------
	Burkholderia ambifaria MC40-6	172064078	---------E-----------I--------	---	-----------------K-----------
	Burkholderia cepacia GG4	402568047	---------E-----------I--------	A--	-----------------K-----------
	Burkholderia mallei ATCC 23344	77358750	-----------A-S-----G---EL-AAAR	ADG	T---L------------N-----------
	Burkholderia vietnamiensis G4	134292174	---------A-D-L-------I--------	AAE	-----------------K-----------
	Burkholderia oklahomensis	497805043	---L-----E-A-Y-----G---EA-ASAR	ADG	T-A-L------------K-----------
	Burkholderia gladioli BSR3	330819791	-----------PAT--E--G---EPNAAAN	AEG	L----------------K-----------
	Burkholderia glumae BGR1	238023951	---------E-PAL--E--G---EP-ASAN	ADG	L----------------K-----------
	Burkholderia sp. KJ006	387903835	---------A-D-L-------I--------	AAE	-----------------K-----------
	Burkholderia pyrrocinia	515900771	-----------A-T-------I--------	AT-	----L------------------------
Other *Burkholderia*	Burkholderia phymatum STM815	186472191	--------S--P---I---S----RPF-GD		TA--L------------N-----------
	Burkholderia sp. BT03	495020832	-----------P-L-----S----GPF-GD		TA--L------------N-----------
	Burkholderia terrae	494851809	-----------P-L-----S----GPF-GD		TA--L------------N-----------
	Burkholderia sp. SJ98	495629595	-----------PS-----------GPF-GD		TA--L------------K-----------
	Burkholderia xenovorans LB400	91778508	---L----S--PS--I---S----GPF-GD		TA--L------------K----M------
	Burkholderia sp. Ch1-1	494316231	---L----S--PS--I---S----GPF-GD		TA--L------------K----M------
	Burkholderia sp. RPE64	507527032	-----------PSY---------SGPF-GD		TA--L------------K-----------
	Burkholderia sp. CCGE1003	307727855	---L----A--PS--I---S----GPF-GD		TA--L-------F----K----M------
	Burkholderia sp. YI23	377813523	-----------PSI---------SGPF-GD		TA--L------------K-----------
	Burkholderia phenoliruptrix BR	407710903	---L----A--PT--I---S----GPF-GD		TA--L-------F----K----M------
	Burkholderia sp. CCGE1001	323530100	---L----A--PT--I---S----GPF-GD		TA--L-------F----K----M------
	Burkholderia graminis	492938001	---L----A--PS--I---S----GPF-GE		TA--L------------K----M------
	Burkholderia sp. H160	496204437	---L----A--AS--I---S----GPF-GD		TA--L------------K----M------
	Burkholderia phytofirmans PsJN	187919753	---L----S--PS--I---S----GPF-GE		TAA-L------------K----M------
	Burkholderia sp. CCGE1002	295699473	---L----SE-AS--I---S----GPF-GE		TA--L------------R----M----D-
Other Bacteria	Pseudogulbenkiania ferrooxidan	496242150	---L---KPL-PK------G----QP-SGG		M---L-------F----A----M--M---
	Ralstonia sp. PBA	497208945	---L----AK-PS---Q--G--L-APCTER		IA----------M--T-N----M------
	Collimonas fungivorans Ter331	340788263	---L----SE-KDV-----S--L-EPSNEP		IAD---------F---------F-LI---
	Pseudogulbenkiania sp. NH8B	347539157	---L---KPL-PAC-----G----QP-SGG		M---L-------F----A----M--M---
	Achromobacter piechaudii	493249878	--------AR-PAV-----S--MHRQSRAP		IA----------M--T-N-----GW--D-
	Verminephrobacter aporrectodea	497794174	---L----PE-PSV--S-----MESAHVGA		HN--L-------H-AF-G--------A--
	Verminephrobacter eiseniae EF0	121610964	---W-----Q-PSV--------MDSARTGA		HNR-L-------H-AF-G--------AA-

Supplemental Figure 5: Partial sequence alignment of a conserved region of sarcosine oxidase subunit alpha showing a 3 amino acid insertion (boxed) that is uniquely shared by the Clade I species, *Ralstonia solanacearum*, and *Ralstonia eutropha*.

Group	Species	ID	260		294
Clade II *Burkholderia*	Burkholderia phytofirmans PsJN	187919777	GQLIELFEPPVITERGYLLV		TQRTATPVVSAFRHW
	Burkholderia sp. WSM2232	548691252	---V---DA--V-----M--		---A---A-D---Q-
	Burkholderia sp. WSM3556	548691604	---V---GA--V-----M--		---A---A-D---Q-
	Burkholderia sp. WSM2230	548606704	---V---DV--V-----M--		---A---A-D---Q-
	Burkholderia sp. URHA0054	522812183	---V---DA--V-----M--		---A---A-D---Q-
	Burkholderia bryophila 376MFSha3.1	482970435	---V---DT--V-----M--		---A---A-E---Q-
	Burkholderia xenovorans LB400	91778532	---V---D---V-----M--		-------A-N---Q-
	Burkholderia sp. Ch1-1	494316277	---V---D---V-----M--		---A---A-----Q-
	Burkholderia graminis	492937910	---V---DA--V-----M--		---A---A-D---Q-
	Burkholderia sp. CCGE1003	307727876	---V---DA--V-----M--		---A---A-D---Q-
	Burkholderia phenoliruptrix BR	407710924	---V---DA--V-G---M--		---A--SA-E---Q-
	Burkholderia sp. CCGE1001	323530121	---V---DA--V-G---M--		---A--SA-E---Q-
	Burkholderia sp. YI23	377813494	---VA-CDA-LV-D---V--		-P-E-SAA-R--SE-
	Burkholderia sp. RPE64	507527007	---VA-CDA-LV-----V--		-P-EPSSA-R--SE-
	Burkholderia sp. SJ98	495629563	---VA-CDA-LV-D---V--		-P-EPSAA-R--SE-
Other *Burkholderia*	Burkholderia sp. KJ006	387903787	---VK-VDA--V-S---F--	RP	P--PE-DATHV--R-
	Burkholderia ambifaria AMMD	115359231	---VK-VDA--V-S---F--	RP	P--PE-DATHV--R-
	Burkholderia vietnamiensis G4	134292128	---VK-VDA--V-S---F--	RP	P--PE-DATHV--R-
	Burkholderia sp. TJI49	497381553	---VK-VDA--V-S---F--	RP	P--PEPDATHV--R-
	Burkholderia sp. 383	78061436	---VK-VDA--V-S---F--	RP	P--PEPDATHV--R-
	Burkholderia ubonensis	497775710	---VK-VDA--V-S---F--	RP	P--PEPDATHV--R-
	Burkholderia cepacia GG4	402568114	---VK-VDA--V-S---F--	RP	P--PEPDATHV--R-
	Burkholderia multivorans	493440937	---VK-VDA--V-S---F--	RP	P--PEPDATHV--R-
	Burkholderia cenocepacia MCO-3	170737571	---VK-VDA--V-S---F--	RP	P--PEPDATHV--R-
	Burkholderia dolosa	493818829	---VK-VDA--V-S---F--	RP	P--PEPDATHV--R-
	Burkholderia gladioli BSR3	330819977	---VRCV-ETAV-----F--	RP	PS-PEA-A-GR--R-
	Burkholderia pseudomallei	490670125	---VR-VDA--V-P---Y--	RP	PA-PEARA-PL--R-
	Burkholderia thailandensis	497586787	---VR-VDA--V-P---Y--	RP	PA-PEARA-PL--R-
	Burkholderia mallei ATCC 23344	53717106	---VR-VDA--V-P---Y--	LP	PA-PEARA-PL--R-
Other Bacteria	Polaromonas sp. CF318	495139023	-R-VK-NDK-F-SP-N-F--	TT	HE-AKA--IQSL-Q-
	Cupriavidus sp. HMR-1	495925593	--V-AP-DL-IRLN---F-C	AP	RE-RDH-ALT---D-
	Cupriavidus metallidurans CH34	94312440	--V-AP-DL-IRLN---F-C	AP	RE-RDH-ALT---D-
	Achromobacter piechaudii	493247437	---AVP-DL-ITL----F-C	IP	SN-RPP-GYG---N-
	Pseudogulbenkiania ferrooxidan	496240818	---VS-VDA-IT-P---F--	LR	ESTRL-G-IGR--R-
	Pseudogulbenkiania sp. NH8B	347539872	---VS-VDA-IT-P---F--	LR	ESTRL-G-IGR--R-
	Verminephrobacter eiseniae EF0	121608949	-R-VR---RS-PAKHA-YM-	CR	HEMS-N-L-A--TE-

Supplemental Figure 6: Partial sequence alignment of a LysR family transcriptional regulator showing a 2 amino acid deletion that is uniquely shared by the Clade II species.

Group	Organism	GI	110		158
Clade Ia *Burkholderia*	Burkholderia multivorans ATCC	189352411	RRDFAANSLVLIVPADSHATP	P	ASLRDLTAPGVKRIAYGDPASVPVGRY
	Burkholderia dolosa	84311095	-----------------RSPA	-	---N-----A-----------------
	Burkholderia ubonensis Bu	167590694	-----------------RTAA	-	------A--------------------
	Burkholderia sp. TJI49	416964351	-----------------Q-NA	-	T--------------------------
	Burkholderia cenocepacia MCO-3	170736049	-----------------R-AA	-	T--N-----------------------
	Burkholderia sp. 383	78063083	-----------------K-AA	-	T--N-----------------------
	Burkholderia cepacia GG4	402569629	-------------------AA	-	T--N-----------------------
	Burkholderia vietnamiensis G4	134292945	-----------------RTPA	-	TT-K--SG-------------------
	Burkholderia sp. KJ006	387904695	-----------------RTPA	-	TT-K--SG-------------------
	Burkholderia ambifaria MC40-6	172062789	-----------------R-AA	-	T--N---V---R---------------
	Burkholderia pyrrocinia	515899567	-------------------AA	-	---H—A-------V-------------
Other *Burkholderia*	Burkholderia thailandensis TXD	167577141	-----------------R--F		G-----S--A---V-F-----------
	Burkholderia pseudomallei DM98	167724631	-----------------R--F		G--------A---V-F-----------
	Burkholderia mallei SAVP1	121597483	-----------------R--F		G--------A---V-F-----------
	Burkholderia oklahomensis EO14	167566534	-----------------R--F		G--------A---V-F-----------
	Burkholderia sp. H160	209520614	--------------S--RFA-		---N---TAN---V-F-----------
	Burkholderia graminis C4D1M	170691098	-K---------------RFA-		S--NE-ASSN-----------------
	Burkholderia sp. CCGE1003	307726570	-K---------------RFA-		S--AE--SSN-----------------
	Burkholderia sp. CCGE1001	323529224	-H---------------RFA-		S--NE-ASSN-----------------
	Burkholderia phytofirmans PsJN	187921437	-K------------T--RFA-		TA-N---SAN---V-------------
	Burkholderia terrae BS001	390569908	-K------------T--KLA-		SN-NE--SAS---VS------------
	Burkholderia sp. CCGE1002	295700807	------------------FA-		-N-NA--TAN---V-F-----------
	Burkholderia xenovorans LB400	91777289	-K------------T---F--		T--N--ASAS---V---------I---
	Burkholderia sp. Ch1-1	385206438	-K------------T--RFA-		T--N---SAS---V---------I---
	Burkholderia phymatum STM815	186473223	-K------------T--KLA-		SN-NE--SAS-R---------------
Other Bacteria	Ralstonia eutropha JMP134	73540340	-------QI-M---Q--KLNL		G--Q---K-E---V-F-N---------
	Cupriavidus basilensis OR16	374370848	-------Q-A--L----KLEI		GK-Q--AR-EF--V-F-N---------
	Cupriavidus necator N-1	339324818	--N----QV--------KLAI		---Q---R-E-------N---------
	Collimonas fungivorans Ter331	340786754	-KN--N-QI--------QLRI		QQ-Q--AQ-A---V---N---------
	Cupriavidus taiwanensis LMG 19	194288777	--N----QV--------KLPI		---Q---R-E-R-----N---------
	Cupriavidus sp. HMR-1	430804675	-K-----QV-----Q--KLGI		G--K---RAD---V---N-S-------
	Cupriavidus metallidurans CH34	94309516	-K-----QV-----Q--KLGI		G--K---RAD---V---N-S-------
	Achromobacter xylosoxidans A8	311107571	-V-----QV--------K-NI		TA-K---RDD-------N---------
	Achromobacter piechaudii ATCC	293606431	-V-----QI-----E--K-GI		T--K---RDDI------N---------
	Achromobacter arsenitoxydans S	359800621	-V-----QI-----T--K-GI		NA-K---RAD-------N---------
	Herbaspirillum sp. CF444	399020386	-KN----QI-----N--PLSL		T--K---QA---KV-L-N-----F---
	Janthinobacterium sp. PAMC 257	395760485	-Q--VS--------H--KLGI		N--N---RK--T-V-IAN---------
	Ralstonia solanacearum CFBP295	300696641	----V--Q------SA-AVPV		HA-G--AR-D-Q-V-I-N---------

Supplemental Figure 7: Partial sequence alignment of a conserved region of a Molybdate ABC transporter substrate-binding protein showing a 1 amino acid insertion (boxed) that is uniquely shared by the Clade Ia species.

Group	Species	ID	305		338
Clade Ia *Burkholderia*	Burkholderia multivorans CGD2	221203041	IGAQPPRYPDAHKHP	H	AAKLLSVLDGDDPAGTRL
	Burkholderia cenocepacia AU 10	107026274	-S----L--------	-	------KVE---------
	Burkholderia sp. 383	78062369	-S----L--------	-	------KVE---------
	Burkholderia ubonensis Bu	167584240	------L--------	Q	------AVE---------
	Burkholderia dolosa AU0158	254254100	-S----L--------	-	-D----K-E---------
	Burkholderia ambifaria IOP40-1	170701409	-S----I--------	-	------KV----------
	Burkholderia sp. KJ006	387904967	-S----V--------	Q	-T----KV----------
	Burkholderia vietnamiensis G4	134293680	-S----V--------	Q	-T----KV----------
	Burkholderia cepacia GG4	402568894	-S----M--------	-	------KV----------
	Burkholderia sp. TJI49	497376881	-S--A----------	-	-------VE----T----
	Burkholderia pyrrocinia	515898831	-S--A-L--------	Q	-K-----VE----T----
Other *Burkholderia*	Burkholderia glumae BGR1	238025833	-S--A-F---IATS-		-KS-V--V----------
	Burkholderia phenoliruptrix BR	407709876	-S--A-F---I-NS-		-K-MV--VE----T----
	Burkholderia sp. CCGE1001	323529034	-S--A-F---I-NS-		-K-MV--VE----T----
	Burkholderia gladioli BSR3	330815124	-S--A-F---IASS-		-KH-A--VE---------
	Burkholderia sp. H160	209515930	-S--A-Y---I-NS-		-K--V--VE----T----
	Burkholderia pseudomallei 91	167817914	-S--A-L---V-QS-		-KH-V--VE---------
	Burkholderia sp. CCGE1003	307726514	-S--A-F---I-NS-		-K--V--VE----T-A--
	Burkholderia thailandensis TXD	167582737	-S--A-L---V--S-		-KH-V--VE---------
	Burkholderia sp. Ch1-1	385206195	-S--A-F---IQNS-		-K-MV--VE----T----
	Burkholderia sp. CCGE1002	295700618	-S--A-Y---I-NS-		-K-MV---E----T----
	Burkholderia xenovorans LB400	91779916	-S-RA-F---IQNS-		-K-MV--VE---------
	Burkholderia mallei	8572766	-S--A-L---V-QSA		-KH-V--VE---------
	Burkholderia phytofirmans PsJN	187921680	-S--A-F--NIQNS-		-K-MV--V-----T-A--
	Burkholderia oklahomensis EO14	167564528	-S--A-L---VQ-S-		-KH-V--VE----T-A--
	Burkholderia graminis C4D1M	170690850	-S--A-F---I-NS-		-K-MV--VE----T-V--
	Burkholderia sp. SJ98	384924444	-S--A-F---VKGS-		-----A-VE------S--
	Burkholderia sp. YI23	377812895	-S--A-F---VKNG-		-----A-IE----K----
	Burkholderia sp. BT03	398042607	-S--A-LV--IGSG-		GK--V--VE----T----
	Burkholderia terrae BS001	390572622	-S--A-LV--IGSG-		GK--V--VE----T----
	Burkholderia phymatum STM815	186474141	-S--A-LV--IGNGT		GK--V--------L----
Other Bacteria	Collimonas fungivorans Ter331	340787460	VSG-T-F---V-NS-		-RHNVATVE----K----
	Herbaspirillum sp. YR522	398836595	-S-TA-Y--NVASSV		-RTQVARTV-G--LS-Q-
	Achromobacter piechaudii HLE	400197016	VAGR--F---VADSV		-KTQIAQ-QSA---DP--
	Pseudomonas sp. R81	408483976	-S--V-F----ANSV		-KSQIAT-Q----T----
	Frateuria aurantia DSM 6220	383318193	VA----F--K-DQS-		-RFSIAQVE---------
	Rhodanobacter sp. 115	389770655	VA----Y--H-DTS-		-KLQIARIE----T-LV-
	Serratia odorifera 4Rx13	270260928	-C-R--F---VQNSI		-KEGIAQ-ES--A-DP--
	Sphingomonas elodea ATCC 31461	383641399	VA-R--F----D-S-		-RGRIAQVE----T-I--
	Zymomonas mobilis subsp. pomac	338708048	-A----F--Y-NRS-		S-G-IAQIE-N--S-I—

Supplemental Figure 8: Partial sequence alignment of a conserved region of acid phosphatase showing a 1 amino acid insertion (boxed) that is uniquely shared by the Clade Ia species.

Group	Organism	GI	137	(boxed)	204
Clade Ib *Burkholderia*	Burkholderia mallei ATCC 23344	53716883	RQPYLASVLPMYAFFALLVPLALPVARGRWW	W	LLVAVSAAIWLAARRIAGYLPTVDGVPWDFNPFAWQ
	Burkholderia pseudomallei K962	53721966	-------------------------------	-	------------------------------------
	Burkholderia oklahomensis	497794626	---------------------------S---	-	--A-G---L-----K-------A-------------
	Burkholderia thailandensis	497583067	------------T-----------I--S-G-	-	----A---T--G--E--A------------------
Other *Burkholderia*	Burkholderia glumae BGR1	238024769	----------------A-------L--DKP-		--L---LL---S----------A-------------
	Burkholderia gladioli BSR3	330820305	-------------I--A-------L---KP-		--L-I-LLG--S---------G--M-----------
	Burkholderia sp. 383	78062083	---------------------T--L--TQP-		--L-F-GSL-F--PHV-RF----E-A----------
	Burkholderia sp. CCGE1003	307727748	--------------------MI--L--SKP-		--L-G-V-L-AG-PS--A---ASPDMH---------
	Burkholderia sp. CCGE1002	295699390	--------------------AV--L--SKP-		----G-V-L-AG-PA-SK---AAPDMH---------
	Burkholderia graminis	492940891	--------------------MI--L--SKP-		--L-G-V-L-AG-PA-NA---AAPDMH---------
	Burkholderia sp. CCGE1001	323529944	--------------------MI--L--SKP-		--L-G-V-L-AG-PA-NA---AAPDMH---------
	Burkholderia phenoliruptrix BR	407710774	--------------------MI--L--SKP-		--L-G-V-L-AG-PA-NA---AAPDMH---------
	Burkholderia phytofirmans PsJN	187919650	--------------------MI--L--SKP-		--L-G-V-L-AG-PA-VA---AAPDMH---------
	Burkholderia sp. Ch1-1	494316047	--------------------MI--L--SKP-		--L-G-V-L-AG-PA-DA---AAPDMH---------
	Burkholderia xenovorans LB400	91778408	--------------------MI--L--SKP-		--L-G-V-L-AG-PA-DA---AAPDMH---------
	Burkholderia sp. H160	496198608	--------------------TI--L--SKP-		--L-G-V-L-AG-PV-SQ---AAPDMH---------
	Burkholderia phymatum STM815	186472139	----------------FA--VI--L--SKP-		--L-G-L-V-GI-PS-LPH--VAENAQ---------
	Burkholderia vietnamiensis G4	134293811	------------------------L--TQP-		--L-F-GSL-Y--PHA-RF----E------------
	Burkholderia ambifaria MC40-6	172063517	------------------------L--TQP-		-ML-F-GSL-Y--PHV-RF----E-A----------
	Burkholderia cepacia GG4	402568588	------------------------L--TQP-		-ML-F-GSL-Y--PHV-RF----E-A----------
	Burkholderia cenocepacia J2315	206563639	------------------------L--TQP-		-ML-F-GSL-Y--PHV-RF----E-A----------
	Burkholderia terrae	497456047	----------------FA--AI--L--SKP-		--L-G-L-V-SI-PSLLPH--VAEDAQ---------
	Burkholderia sp. BT03	495023261	----------------FA--AI--L--SKP-		--L-G-L-V-SI-PSLLPH--VAEDAQ---------
	Burkholderia ubonensis	497775423	------------------A-----L--TQP-		--L-F-TSL-F--PHA-------E-T----------
	Burkholderia rhizoxinica HKI 4	312796297	-----------------A--VII-L--S-P-		--A-C-L-L-CL-GDAYWL--AAQENR---------
	Burkholderia dolosa	493819095	------------VL---AS-VIV-F--RHP-		----F---S-I--SWLGPE-LDT--FR-S-------
	Burkholderia multivorans	493454502	------------------A-----L--TQP-		--L-F-GSL-Y--PHA-RF-----------------
	Burkholderia sp. TJI49	497378265	------------VL---AS-ALV-F--RHP-		----L-V-S----GWLGPE-LDT-TFR-S-------
	Burkholderia sp. YI23	377812665	------A----------A--VM--L--SKP-		--LGL-L-L-AF-AQ-GEHM-S--DNL-----A---
	Burkholderia sp. RPE64	507526539	------A------L---A---V--L--SKP-		--LGL-L-L-AF-AQVGEHM-S--DNL-----A---
	Burkholderia sp. SJ98	495628550	-----SA------L---A---M--L--SKP-		--LGL-L-L-AF-AQ-GEFM-S--DNL-----A---
	Burkholderia sp. KJ006	387904094	------------VL---AS-VLV-F--RHP-		-T--F-V-S-G--GWLGPE-LDT-SFR-S-------
Other Bacteria	Cupriavidus basilensis	493149580	---FVSD------V-I-FA---IRM--AMPL		TF-GA--LL--M-PMLTPL--SS--NG-S-------
	Cupriavidus sp. HMR-1	495921183	---FVSD------V-IG-A---IGW--KYPL		AF-CG-V-L-IM-PALLPL--SSEPRG-S-------
	Cupriavidus metallidurans CH34	94313808	---FVSD------V-IG-A---IGW--KYPL		AF-CG-V-L--M-PALLPL--SSEPRG-S-------

Supplemental Figure 9: Partial sequence alignment of a conserved region of opgC protein showing a 1 amino acid insertion (boxed) that is uniquely shared by the Clade Ib species.

Group	Species	ID	29		63
Clade Ib *Burkholderia*	Burkholderia pseudomallei DM98	167725414	ILVYHRFSTSAP	P	DSMTVRVSTFGAQLAFLRAHGY
	Burkholderia mallei ATCC 10399	254176506	------------	-	----------------------
	Burkholderia thailandensis Bt4	167617303	------------	-	----------D-----------
	Burkholderia oklahomensis C678	167574106	----------T-	-	------I---E-----------
Other *Burkholderia*	Burkholderia phytofirmans PsJN	187920294	-------AS-VN		----------N---R---ER--
	Burkholderia sp. Ch1-1	385209223	-------AATVN		-------A--E---R--KER--
	Burkholderia dolosa AU0158	254255487	-------AEQRV		-------H--IE--RVID----
	Burkholderia ambifaria MC40-6	172065489	-------A-ARL		------TD--RN--QAIE-N--
	Burkholderia cenocepacia J2315	206562178	-------AAARL		------ID--RN--QAID-N--
	Burkholderia sp. TJI49	416924263	-------A-ARL		------TE-LRN--QAIE-N--
	Burkholderia multivorans ATCC	161522897	-------A-ARL		------TE-LRN--RAIE-N--
	Burkholderia vietnamiensis G4	134291694	--A----A-TRV		------ID-LRD--QAID-N--
	Burkholderia sp. KJ006	387906399	--A----A-TRV		------ID-LRD--QAID-N--
	Burkholderia gladioli BSR3	330816982	--A----A-ERR		------TV--LR--QAIE-N--
Other Bacteria	Pseudogulbenkiania ferrooxidan	224825925	-------AATVT		------S---EDH-RV--SQ--
	Pseudogulbenkiania sp. NH8B	347540540	-------AATVT		------SA--EDH-RV--SQ--
	Ralstonia pickettii 12J	187926584	V------APTVD		ST----MQ--E-H-QAIE-A--
	Ralstonia sp. 5_7_47FAA	309782692	V------APTVD		ST----MQ--E-H-QAIE-A--
	Azoarcus sp. KH32C	358636512	V------GAV-A		------TEV-E---QQ-H-S-V
	Pseudomonas sp. GM18	398999936	--------DT-D		------M---ET--R--HE---
	Acidithiobacillus ferrooxidans	218665767	--L----GPVLR		-A----TMV-A--MEY--S---
	gamma proteobacterium NOR5-3	254515067	--L---V-DTG-		--TR-TPAR-A-H-DLIKDR--
	Pseudoalteromonas arctica A 37	392535643	--Q--HV-ETL-		AVTS-SAN--TKH-SY-KE-NF

Supplemental Figure 10: Partial sequence alignment of a conserved region of polysaccharide deacetylase family protein showing a 1 amino acid insertion (boxed) that is uniquely shared by the Clade Ib species.

Group	Species	ID	247		294
Clade Ib *Burkholderia*	Burkholderia pseudomallei	497613277	LRDPKRARADYDVLVAAPAEVVAYL	A	GGDAAARALLAKQYDAALARLS
	Burkholderia mallei ATCC 23344	53715913	-------------------------	-	----------------------
	Burkholderia oklahomensis	497794971	-------------------D--K--	-	--------P----F--------
	Burkholderia thailandensis MSM	488606631	-------------------D--R--	-	-D------S----F--------
Other *Burkholderia*	Burkholderia ambifaria AMMD	115359601	--E-VL----S--------R-----		-----Q--K-RGA---------
	Burkholderia sp. KJ006	387904079	----PL----S--------R-----		--EP-Q--N-RGA---------
	Burkholderia cepacia GG4	402570425	--E-ALS---S--------R-----		----TQ--T-RAA--T------
	Burkholderia vietnamiensis G4	134292408	----PL----S--------R-----		--EP-Q-TK-RGA---------
	Burkholderia sp. 383	78060978	-Q--ALS---S--------R-----		---D-Q-TK-RGA---------
	Burkholderia ubonensis	497775965	---AALS--NA--------G-----		---D-R--Q-RSA---------
	Burkholderia cenocepacia MCO-3	170737100	-H-AALS---S--------R-----		---D-Q-SK-RGA--T------
	Burkholderia gladioli BSR3	330816915	-N--RLS-EQS--------DL-RW-		AK-S-E--R-S-A-----T---
	Burkholderia dolosa	493819082	---AALS---S--------R---F-		-S-DTQ--A-RGA-GT------

Supplemental Figure 11: Partial sequence alignment of a conserved region of thioredoxin domain protein showing a 1 amino acid insertion (boxed) that is uniquely shared by the Clade Ib species.

Group	Species	GI	129		160
Clade Ic *Burkholderia*	Burkholderia gladioli BSR3	330820376	GKGGLSQWMLKRARAIGGA	T	LIEASAFHLRAD
	Burkholderia glumae BGR1	238024743	--ES--R---G--------	A	------W-I-V-
Other *Burkholderia*	Burkholderia multivorans CF2	400229634	--VA--R-L-A---ET-S-		-L--NGW-V---
	Burkholderia sp. TJI49	325524485	--AA--R---RK--ET-S-		-L---GW-V-T-
	Burkholderia sp. 383	78062468	--AV--H---RK--ET-S-		-L---GW-V-T-
	Burkholderia cenocepacia AU 10	107026320	--AV--Y---RK--ET-S-		-L---GW-V-T-
	Burkholderia ambifaria MC40-6	172063306	--AA--R---RK--ET-S-		-L---GW-V-T-
	Burkholderia dolosa AUO158	254254042	--AA--H---SN--ET-S-		-L---GW-V-T-
	Ralstonia solanacearum CFBP295	300702726	A-E--FRY--AV--R--SR		MLI-N-W-A-S-
	Ralstonia pickettii 12J	187930422	A-E--FRY--AM-KR-NSR		MLI-N-W-A-S-
	Ralstonia sp. 5_7_47FAA	309780591	A-E--FRY--AM-KR-NSR		MLI-N-W-A-S-
	Burkholderia vietnamiensis G4	134294358	S-E--FRY--RE--RVRS-		MLV-N-W-A-S-
	Burkholderia sp. KJ006	387900968	S-E--FRY--RE--RVRS-		MLV-N-W-A-S-
	Burkholderia phymatum STM815	186474887	S-E--FRY--RE-QRVRS-		MLI-N-W-A-S-
	Curvibacter putative symbiont	260221880	T-EL-FRY--RT-K-VKSS		-LV-N-W-A-S-
	Ralstonia syzygii R24	344172781	-E--FRY--AV--R--SR		MLI-N-W-A-S-
	Burkholderia pseudomallei 1655	254183857	S-E--FRY--RE-QRVRS-		MLV-N-W-A-S-
	Burkholderia graminis C4D1M	170693939	S-E--FRY--RE-QRVRS-		MLI-N-W-A-S-
	Burkholderia cenocepacia PC184	254246785	S-EA-FRY--RE--RVRS-		MLV-N-W-A-S-
	Burkholderia ambifaria IOP40-1	170701643	S-EA-FRY--RE--RVRS-		MLV-N-W-A-S-
	Burkholderia sp. 383	78064862	S-EA-FRY--RE--RVRS-		MLV-N-W-A-S-
	Burkholderia cepacia GG4	402565000	S-EA-FRY--RE--RVRS-		MLV-N-W-A-S-
	Burkholderia phytofirmans PsJN	187922406	S-ES-FRY--RE-QRVRS-		MLI-N-W-A-S-
Other Bacteria	Thioalkalivibrio thiocyanoxida	350559857	A-E--Y-YTVRV--R--SP		-LH-N-W-H-S-
	Providencia stuartii ATCC 2582	183599828	A-E--FRY---V-NQVNSS		MLV-N-W-A-S-
	Thioalkalivibrio sulfidophilus	220936414	F-E--Y-YTIRV--R-RSN		-LK-N-W-H-S-
	Providencia rettgeri DSM 1131	268591690	A-E--FRY--HV-KKVNSS		MLV-N-W-A-S-
	Klebsiella pneumoniae subsp. r	262044483	V-E--FRY--AA--RLNSS		-LI-N-W-A-S-
	Klebsiella sp. 4_1_44FAA	365138183	V-E--FRY--AA--RLNSS		-LI-N-W-A-S-
	Klebsiella sp. MS 92-3	330010635	V-E--FRY--AA--RLNSS		-LI-N-W-A-S-
	Thioalkalivibrio sp. K90mix	289209735	A-E--Y-YTHRV-KR--SR		MLD-N-W-H-T-
	Proteus penneri ATCC 35198	226328219	A-E--FRY--AV-KKVDSN		MLV-N-W-A-S-

Supplemental Figure 12: Partial sequence alignment of a conserved region of cation efflux protein showing a 1 amino acid insertion (boxed) that is uniquely shared by the Clade Ic species.

			155		198
Clade Ic *Burkholderia*	Burkholderia glumae BGR1	238024763	VKTGDIVRQGEPIAQAGAPDRAR	D	AAVCFEVRRDGKPVDPVAYL
	Burkholderia gladioli BSR3	330820315	--------------E--GAGWV-	G	T--L----------------
Other *Burkholderia*	Burkholderia cepacia GG4	402568596	----------DE--EM-DL-N--		V-LL----------N-IP--
	Burkholderia ambifaria	493808261	----------DA--EM-DL-NS-		V-LL----------N-MP--
	Burkholderia ubonensis	497783919	-----V----DE--EM-DL-NS-		V-LL----------N-LP--
	Burkholderia sp. 383	78062094	----------DE--EM-DL-NS-		V-LL----------N-MP--
	Burkholderia sp. KJ006	387904839	----------DA--EM-DV-N-H		V-LL----------N-MP--
	Burkholderia vietnamiensis G4	134293806	----------DA--EM-DV-S-H		V-LL----------N-MP--
	Burkholderia thailandensis	497583060	-----V-----Q--EM-TG-ST-		-GML----------N-MQ--
	Burkholderia pseudomallei	497619586	-----V-Q---Q--EM-TG-ST-		-GML----------N-MQ--
	Burkholderia cenocepacia AU 10	107026122	-------H--DA--EM-DL-NS-		V-LL----------N-MP--
	Burkholderia sp. H160	496198610	--------K-QQ--EM-DENNS-		VS-G------------LS--
	Burkholderia multivorans	493441411	-----V-H--DE--EM-DL-NS-		V-LL----------N-LP--
	Burkholderia terrae	494853027	--------R-QQ--EM-SENNS-		VS-L--L------I--MPF-
	Burkholderia sp. CCGE1003	307727751	----------QQ--EM-DENNS-		VS-G--L---------MP--
	Burkholderia sp. BT03	495006908	--------R-QQ--EM-SENNS-		VS-L--L------I--MPF-
	Burkholderia phymatum STM815	186472124	--------K-QQ--EM-NENNS-		VS-L--L-H----I--MP--
	Burkholderia oklahomensis	497794632	-----V-Q---Q--EM-IG-S--		-GML----------N-MQ--
	Burkholderia mallei SAVP1	121597179	-----V-Q---Q--EM-TG-ST-		-GML----------N-MP--
	Burkholderia phenoliruptrix BR	407710778	----E-----QQ--EM-DENNS-		VS-G--L---------MP--
	Burkholderia sp. CCGE1001	323529948	----E-----QQ--EM-DENNS-		VS-G--L---------MP--
	Burkholderia graminis	492940874	----E-----QQ--EM-DENNS-		VS-G--L---------MP--
	Burkholderia phytofirmans PsJN	187919659	-------A--QQ--EM-DENNS-		VS-G--L------I--MP--
	Burkholderia xenovorans LB400	91778414	-------V--QQ--EM-DENNS-		VSLG--L------I--MP--
	Burkholderia sp. CCGE1002	295699392	--M-----K-QQ--EM-DENNS-		VS-G----H-------LR--
	Burkholderia sp. Ch1-1	494316056	-------V--QQ--EM-DENNS-		VSLG--L------I--MP--
	Burkholderia sp. TJI49	497378896	--E--A-TK-QK--EM-NS-SD-		VMLH-----Q------LK--
	Burkholderia rhizoxinica HKI 4	312796083	--E--P-T--QT--EM-GS-ADK		VMLH----KQ------MK--
	Burkholderia sp. RPE64	507516129	--E--A-TK-QK--EM-NS-SD-		VMLH-----Q------LK--
	Burkholderia sp. SJ98	495624781	--E--A-TK-QK--EM-NS-SD-		VMLH-----Q------LK--
	Burkholderia sp. YI23	377820998	--E--A-TK-QK--EM-NS-SD-		VMLH-----Q------LK--
	Burkholderia dolosa	493816625	--E--A-TK-QK--EM-NS-AD-		VMLH-----Q------LK--
Other Bacteria	Pandoraea sp. SD6-2	498506635	--E--S-TR-QK--EM-NS-AD-		VMLH------------MK--
	Pseudogulbenkiania sp. NH8B	347538487	--E-QA-KK-Q---EM-NT-ADQ		VKLH--I--F------LQ--
	Neisseria sp. oral taxon 020	496977268	--E--F-KR-QT--RM-NT-AD-		VKLH----Q-----N--N-V
	Bordetella holmesii	491160778	--Q-QS-K--QQ--EM-NS-ASS		NQLY--L---------TGV-
	Pseudogulbenkiania ferrooxidan	496240019	--E-QA-KK-Q---EM-NT-ADQ		VKLH--I--F------LQ—

Supplemental Figure 13: Partial sequence alignment of a conserved region of putative peptidoglycan-binding LysM/M23B peptidase showing a 1 amino acid insertion (boxed) that is uniquely shared by the Clade Ic species.

Group	Species	Accession	80		130
Clade Ic *Burkholderia*	Burkholderia glumae BGR1	238024002	GSIGTHAEFQGGAITGNATSIFTPLQGK		NLSGTVGRYNRATGQRDLSINVS
	Burkholderia gladioli BSR3	330819826	----------------------------		-Q--F---------L---T----
Other *Burkholderia*	Burkholderia pseudomallei	490683105	----------------------AAM-YS	TP	QG-----------L----T-P--
	Burkholderia mallei NCTC 10229	124381210	----------------------AAM-YS	TP	QG-----------L----T-P--
	Burkholderia thailandensis	497584362	----------------------AAM-YG	TP	QG-----------L----T-P--
	Burkholderia phymatum STM815	186471177	----I-S---------------AAM-AG	TQ	YGT-G---------A---V----
	Burkholderia oklahomensis	497807748	----------------------AA--SS	RT	YG--A--------RT---F-Q--
	Burkholderia sp. BT03	495004854	----I-S---------------AAM-VG	TQ	YG--G---------T---V----
	Burkholderia terrae	494859066	----I-S----S----------AAM-AG	TQ	YG--G---------T---V----
	Burkholderia ubonensis	497783006	----A-G---------------AA--AN	RS	YG--A--------KA---F-Q--
	Burkholderia xenovorans LB400	91779191	-------D---S----------AA--YN	KS	FG--S--------QT---I-P--
	Burkholderia sp. Ch1-1	494318151	-------D---S----------AA--YN	KS	FG--S--------QT---I-P--
	Burkholderia sp. 383	78061259	-T--I-D---------------VA-GYN	RN	FG--S------S-N----R-P--
	Burkholderia sp. TJI49	497374511	----A-G----S----------AA--PG	KT	YG--A--------KV--RL-Q--
	Burkholderia phytofirmans PsJN	187919318	-------D---S----------AA--YN	RS	FG--S------V-QT---I-P--
	Burkholderia sp. CCGE1003	307727331	-------D---S-----------A--YN	RT	FG--S--------KALE-R-P--
	Burkholderia graminis	492930587	-------D---S-----------A--YN	RS	FG--S--------KALE-R-P--
	Burkholderia cenocepacia J2315	206564284	-T--I-D----------S--L-VA-GYN	RT	FG--S------S-N----R-P--
	Burkholderia vietnamiensis G4	134292218	-T--I-D-----------N-L-VA-GYN	RT	FG--S------S-N----R-P--
	Burkholderia sp. KJ006	387903880	-T--I-D-----------N-L-VA-GYN	RT	FG--S------S-N----R-P--
	Burkholderia ambifaria AMMD	115359353	-T--I-D-----------N-L-VA-GYN	RT	FG--S------S-N----R-P--
	Burkholderia sp. CCGE1001	323528391	-------D---S----------VA--YN	RS	FG--S--------KALE-R-P--
	Burkholderia cepacia GG4	402570222	--F---S----S--------L-VA--PG	KG	YG--A--------RF--KL-Q--
	Burkholderia multivorans	493458973	-T--I-D-------------L-VA-GYN	RT	FG--S------S-NA---R-P--
	Burkholderia dolosa	493818919	-T--I-D-------------L-VA-GYN	RT	FG--S----D-S-NK---R-P--
	Burkholderia phenoliruptrix BR	407709240	-------D---S----------VA--YN	RS	FG--S------V-KALE-R-P--
	Burkholderia sp. H160	496197664	----G-G----S----D-----AA--FN	TT	YG--A------T-QT--FL-S—

Supplemental Figure 14: Partial sequence alignment of a conserved region of SMP-30/gluconolaconase/LRE-like region-containing protein showing a 1 amino acid insertion (boxed) that is uniquely shared by the Clade Ic species.

Group	Species	Accession	322	Insertion	358
Clade Ic *Burkholderia*	Burkholderia gladioli BSR3	330821370	LDADYETRASEGEAPWILRDQ	P	REPTPDAGRYLVVID
	Burkholderia glumae BGR1	238025104	---------AQ--L-R----A	-	---------------
Other *Burkholderia*	Burkholderia oklahomensis	497797196	---E--A--AS-----V--AH		-QI--W-------V-
	Burkholderia pseudomallei	497608872	---E--A--AG-DP--V--AH		-QV--W---------
	Burkholderia mallei NCTC 10229	124381799	---E--A--AG-DP--V--AH		-QV--W---------
	Burkholderia thailandensis	497587232	---E--A--AA-DP--V--VH		-QV--W-------V-
	Burkholderia sp. YI23	377813220	---Q--A--A--QSM-V-KAT		-QT--W--H----V-
	Burkholderia sp. SJ98	495629219	---Q--A-V---QSM-V-KAT		-KT--W--H----V-
	Burkholderia ubonensis	497775132	------A--EA-KSF-V-HPG		--I--IE--------
	Burkholderia sp. RPE64	507525967	---Q--A--A--QSM-V-KAT		-KT--W--H----V-
	Burkholderia ambifaria	493805907	--D--QA-VEA-KSL-V--HD		-DI--I---------
	Burkholderia sp. 383	78061236	--D--QA-VEA-KSL-V--RD		-DI--I---------
	Burkholderia cenocepacia J2315	206564303	--D--QA-VEA-KSL-V--RD		-DI--I---------
	Burkholderia dolosa	493818929	--D--QA-VES-KSL-V--RD		-DI--I---------
	Burkholderia multivorans	493453510	--D--QA-VEA-KSL-V--GD		--IS-I---------
	Burkholderia vietnamiensis G4	134292236	--D--QA-VES-RSL-V--HD		-DI--I---------
	Burkholderia sp. TJI49	497377021	--D--QA-VEA-KSF-V--AD		-DI--I---------
	Burkholderia sp. KJ006	387903895	--D--QA-VES-RSL-V--HD		-DI--I---------
	Burkholderia cepacia GG4	402567957	--D--QA-VEA-KSL-V--HD		-DI--I-----A---
	Burkholderia rhizoxinica HKI 4	312797119	--------IR--HFL-V-KPD		--ASRW----I-I--
	Burkholderia phymatum STM815	186473851	--R---ALVA--RSL-V-HSD		-VA--W----I--V-
	Burkholderia sp. Ch1-1	494315651	---E-AALVES-KSL-V-KSD		-QV--W----I--V-
	Burkholderia terrae	494861251	--R---ALVAD-RSL-V-HSD		-VV--W----I--V-
	Burkholderia xenovorans LB400	91778139	---E-AALVES-RSL-V-KSD		-QV--W----I--V-
	Burkholderia sp. BT03	495020379	--R---ALVAD-RSL-V-HSD		-VV--W----I--V-
	Burkholderia sp. CCGE1002	295699264	---E--QLVDS-QSL-V-KSD		-QIM-W---FI--V-
	Burkholderia sp. H160	496202389	---E-A-LVDS-KSL-V-KSD		-QIL-W----I--V-
	Burkholderia phenoliruptrix BR	407710609	---E-AALVDS-RSL-V-KSD		--IV-W----I--V-
	Burkholderia sp. CCGE1001	323529777	---E-AALVDS-RSL-V-KSD		--IV-W----I--V-
	Burkholderia phytofirmans PsJN	187919489	---E-VALVES-KSL-V-KSD		-QV--W----IA-V-
	Burkholderia sp. CCGE1003	307725932	---E-AALVES-KSL-V-KSD		-QIL-W----I--V-
Other Bacteria	Polaromonas sp. CF318	495138436	-----LQSVR--RQL-V--PD		---V-E-------LE
	Polaromonas naphthalenivorans	121603737	--E---RAVAS-DQL-V--ED		-T---SP------V-
	Methylibium petroleiphilum PM1	124267246	-----DAAVAR--SF-V--SD		-LM--W---W---V-
	Variovorax paradoxus S110	239815807	-----LRAKA--S---V-PPD		QA-VEG-----I-V-
	Achromobacter xylosoxidans NH4	507103132	------LA-ALVAP--V-LRD		-T-VAG-----IIV-
	Achromobacter piechaudii	493258358	------LA-DLVST--V--RD		-T-AYG-----I-VE
	Gallionella capsiferriformans	302878947	-----LQALEN--KM-M--AD		-T--AWP----II--
	Herminiimonas arsenicoxydans	134095528	---A--SMQ-A-QTM-V-PSN		ST-VSGP----I-V-

Supplemental Figure 15: Partial sequence alignment of a conserved region of hypothetical protein bgla_2g22890 showing a 1 amino acid insertion (boxed) that is uniquely shared by the Clade Ic species.

			16		76
Clade IIa *Burkholderia*	Burkholderia sp. YI23	377821591	WGVNFVVIKLGLHGVPPMLLGALRFALAAVPVFF		VKRPQIPLRWLFAYSLTISLGQFALLF
	Burkholderia sp. RPE64	507516797	-----------------------------L-A--		--------------G------------
	Burkholderia sp. SJ98	495618174	-------------------------------AI-		--------------G------------
	Candidatus Burkholderia kirkii	494057185	-------------N-------VF--------A--		------A-------GF-----------
Other *Burkholderia*	Burkholderia sp. JPY251	517248995	---------V---------------------AV-	F	-----M-W------GA---F----F--
	Burkholderia sp. CCGE1002	295677512	---------V---------------------AV-	F	-----M-W------GA---F----F--
	Burkholderia kururiensis	516387247	---------V---------------M-----AV-	F	----RV--------GA---F----F--
	Burkholderia sp. CCGE1001	323527187	---------V---------------S-----AV-	F	-----L-W---L--G----F----F--
	Burkholderia sp. H160	496201455	---------V---------------T-----AV-	F	-----M-W------GA---F----F--
	Burkholderia sp. WSM4176	517232824	---------V---------------T-----AV-	F	-----L-W------GA---F----F--
	Burkholderia sp. CCGE1003	307730823	---------V---------------T---L-AV-	F	-R---L-W---L--G---------F--
	Burkholderia graminis	492931008	---------V---------------T---I-AV-	F	-----LAW---L--G---------F--
	Burkholderia phytofirmans PsJN	187925191	---------V---------------------AV-	F	----NL-W---I--GA---F----F--
	Burkholderia bryophila	518913297	---------V---------------M---F-AI-	F	-----L-W---L--GV--------F--
	Burkholderia phymatum STM815	186475286	---------V---------------M---F-AV-	F	I----M-W---L--GA--------F--
	Burkholderia sp. BT03	495017216	---------V---------------M---F-AV-	F	I----M-W---I--GA--------F--
	Burkholderia terrae	494852779	---------V---------------M---F-AV-	F	I----M-W---I--GA--------F--
	Burkholderia sp. Ch1-1	494324911	---------V---------------T-----AV-	F	I---KL-W---L--GA---F----F--
	Burkholderia xenovorans LB400	91785035	--I------V---------------T-----AV-	F	I---NL-W---L--GA---F----F--
	Burkholderia dolosa	493815971	---------V----M----------T-----AV-	F	-R-----W-M-IL-GA--Q----VF--
	Burkholderia multivorans ATCC	189351327	---------V----M----------T-----AV-	F	-R-----W-M-LL-GA--Q----VF--
	Burkholderia cepacia GG4	402565662	---------V----M----------T-----AV-	F	-R-----W-M-IL-GA--Q----VF--
	Burkholderia ambifaria AMMD	115352663	---------V----M----------T-----AV-	F	-R-----W-M-IL-GA--Q----VF--
	Burkholderia sp. TJI49	325519691	---------V----M----------T-----AV-	F	-R-----W-L-LL-GA--Q----VF--
	Burkholderia vietnamiensis G4	134296749	---------V----M----------T-----AV-	F	-R-----W-M-IL-GA--Q----VF--
	Burkholderia lata	78067365	---------V----M----------T-----AV-	F	-R-----W-L-VL-GA--Q----VF--
	Burkholderia glumae BGR1	238028464	-----------------------------L-AV-	F	-P--R----M-VL-GA--L-A--VF--
	Burkholderia thailandensis E26	83718434	---------V-----------G---L-----AV-	F	-R--R----L-AL-GS--L----VF--
	Burkholderia cenocepacia AU 10	107023502	---------V----M----------T-----AV-	F	-R----SW-L-VL-GA--Q----VF--
	Burkholderia oklahomensis	497787827	---------V-----------G---L--S--AV-	F	-R-----W-L-AL-GS--L----VF--
	Burkholderia ubonensis	497776693	--------RV--D-M----------T-----AV-	F	-R-----W-L-VL-GA--Q----VF--
	Burkholderia pyrrocinia	515903421	---------V----M----------T-----AV-	F	-R--R-SW-L-VL-GA--Q----VF--
	Burkholderia pseudomallei	497649922	---------V-----------G---L--S--AV-	F	-R--R--W-L-VL-GS--L----VF--
	Burkholderia mallei ATCC 23344	53725147	---------V-----------G---L--S--AV-	F	-R--R--W-L-VL-GS--L----VF--
	Burkholderia gladioli BSR3	330818129	-------------------------T-----AV-	L	-P--R--W-M-LL-GA--L-A--VF--
	Burkholderia rhizoxinica HKI 4	312795624	---------V----L----------L-S-F-AV-	F	-R--AV-WPLFL--GA------LG---
Other Bacteria	Pandoraea sp. B-6	515801574	---------V---------------L---F-AI-	F	-----V-----I--GA---F-------
	Ralstonia sp. AU12-08	544771944	---------V-----------------V-F-AVL	F	-P--K---K--L--GA--------F--
	blood disease bacterium R229	344168619	------I--V-----------------V-F-AIL	F	-P--K-A-K--L--GI---F----F--
	Cupriavidus metallidurans CH34	94312356	---------V--V------------C-V-F-AV-	F	IP--R----M-V--GA-------VF--
	Curvibacter putative symbiont	260220140	-----A---V-VV----L-----------F-ALL	F	M-P-KV----YL--G----V----F--
	Pseudogulbenkiania sp. NH8B	347538988	---------W-VAD---L-------T---L-AVL	L	-R--AV-W---A--A--VGV---G---
	Leeia oryzae	516894188	---------F----I--LM--G---LT--L-AV-	L	-P--KV-FKLYL--G----V---GF--
	Achromobacter piechaudii	493268863	--L----M-V-MR-LS--M----------F-LIL	F	-R---L-WH-IA--G-VQG----G---
	Chromobacterium violaceum ATCC	34497359	---------W-VA----L---------V-L-AV-	V	IR--AL-W-Y-AL-G--VGV---GC--
	Pseudomonas stutzeri RCH2	431927896	--M-----MI---D-----------M-----AV-	F	I----V----ML--G---------F--
	Pantoea sp. AS-PWVM4	544756798	--------------M--F--AG---T-V-F-AI-	F	----N------VV-GM---F----F--
	Plautia stali symbiont	549490376	------------Q-I--F--AG---T-V-F-AI-	F	-R--A------VV-GM---F----F--
	Vibrio natriegens	520914355	---------V--Q-M--L--AG-----V-F-ALL	F	IP--KV--K--V--G----F-------

Supplemental Figure 16: Partial sequence alignment of a conserved region of hypothetical protein BYI23_A021470 showing a 1 amino acid deletion (unboxed) that is uniquely shared by the Clade IIa species.

Group	Species	ID	179		230
Clade IIa *Burkholderia*	Burkholderia sp. YI23	377821714	IDYDTRYLPDVLTLPLLWAGLIVNFG	E	GGFATLHDAVTGAIAGYLFLWCVYW
	Burkholderia sp. RPE64	507516934	----------I---------------	-	-------------------------
	Burkholderia sp. SJ98	495618382	---------------------L----	-	-------------V-----------
	Candidatus Burkholderia kirkii	494056137	--------------------------	A	---V---------------------
Other *Burkholderia*	Burkholderia phymatum STM815	186477405	--I--HL---SM-------------N		AV--------V-------A----H-
	Burkholderia sp. JPY347	517253793	--IE--L---S--------------A		DT--N-RE--A---V------G---
	Burkholderia graminis	492936834	--M--HL---SM--------I----N		-M--------L---F---V--A-H-
	Burkholderia sp. CCGE1002	295677745	--I--HL---S--------------N		-M--N-----L---F---V--A-H-
	Burkholderia kururiensis	516387433	--L-HQL---SM----------I-LS		ST-VS-----I-------A------
	Burkholderia multivorans	493454338	--MR-G----SM---------VL-L-		-T--S-RS--I--M-------S---
	Burkholderia sp. CCGE1003	307731056	--I--HL---SM--------I----N		-M--S-----L---F---V--A-H-
	Burkholderia sp. Ch1-1	494324369	--I--HL---S---------I----N		-M--N-----L---F---V--V-H-
	Burkholderia xenovorans LB400	91785277	--I--HL---S---------I----N		-M--N-----L---F---V--V-H-
	Burkholderia glumae BGR1	238026148	--IK-G----SM---------V--LW		-S-VD-RE--I------------F-
	Burkholderia sp. CCGE1001	323527414	--M--HL---SM--------IV---N		-V--S-----L---F---V--A-H-
	Burkholderia sp. JPY251	517248807	--I--HL---S-------T------N		-M--N-----L---F---V--A-H-
	Burkholderia phenoliruptrix BR	407714808	--M--HL---SM--------IV---N		-V--S-----L---F---V--A-H-
	Burkholderia cenocepacia	493542906	--IR-G----SM---------VL-L-		-T--S-RS--I--M-------SI--
	Burkholderia gladioli BSR3	330815459	--IK-G----SM---------L--LW		-N-VD-RE--I------------F-
	Burkholderia sp. WSM4176	517233055	--V--HL---S---------V----N		-M-TN-----L---F---V--A-H-
	Burkholderia vietnamiensis	544730105	--IR-G----SM---------VL-L-		-T-TS-RS--I--M-------S---
	Burkholderia phytofirmans PsJN	187925426	--I--HL---SM--------I----N		-M-TN-----L---F---V--V-H-
	Burkholderia ubonensis	497777072	--IQ-G----SM---------AL-L-		-T--S-RA--I--M-------SI--
	Burkholderia lata	78065137	--IR-G----SM---------VL-L-		-T-TS-RS--I--M-------SI--
	Burkholderia pseudomallei	497617772	--MQ-GF---S----------C--LW		-T--S-RA--I-----------IL-
	Burkholderia pyrrocinia	515902897	--IQ-G----SM---------AL-L-		-T--S-RA--I--M-------SI--
	Burkholderia dolosa	493817680	--IR-S----SM---------AA-L-		-T-TS-RS--I--M-------AI--
	Burkholderia ambifaria	493807209	--IQ-G----SM---------AL-L-		-T-TS-RS--V--M-------SI--
	Burkholderia sp. H160	496201347	--I--HL---S--------------N		-M-TN--G--L---F---V--A-H-
	Burkholderia thailandensis	497574621	--MQ-GF---S----------C--LW		-T--S-RA--V-----------IL-
	Burkholderia mallei ATCC 23344	53726065	--MQ-GF---S----------C--LW		-T--S-RA--I-----------IL-
	Burkholderia bryophila	518915362	--I--HL---SM--------I----Y		-V--S-Q---L---F---V--A-H-
	Burkholderia oklahomensis	497792405	--MQ-GF---S----------C--LW		DT--S-RT--I-----------IL-
	Burkholderia cepacia GG4	402567690	--IR-G----SM---------AL-LA		-T-TS-RS--I--M-------SI--
	Burkholderia sp. TJI49	325518037	--IR-G----SM---------AL-LA		DTLTS-RS--I--M-------SI--
	Burkholderia rhizoxinica HKI 4	312795077	--LE-KL---S----------LI-LH		AV--P-DA--I--A----S--A---
Other Bacteria	Thiobacillus denitrificans ATC	74318386	--L--TL---S----------LI-L-		-H--S-P---A--A----V--S---
	Oxalobacteraceae bacterium AB_	522197631	--F--QL---D--Y--I----LM-LN		-T--------I--A----A--S-N-
	Rhodocyclaceae bacterium RZ94	518758947	--L--QL---SI----I----LL-LV		---TD-QS--V-------V--S---
	Janthinobacterium sp. CG3	516490728	--A--QL---D--Y-------M--LN		-T-VP-----V--A----V--SI--
	Massilia niastensis	522140205	--F--QL---D--Y-------L--LN		-T-VP-Q---I--A----V--A---
	Methyloversatilis universalis	517041892	--L--QL---SI----V----LL-LS		---TD-ASS----M----V--T---
	Nitrosomonas eutropha C91	114331043	--LN-QL---SI-Q----------LH		N--VDI-S--I--V----T--S---
	Nitrosospira sp. APG3	490280335	--M--QL---DI------G--L--L-		N--TDI-S-II-TV----A--S---
	Ralstonia sp. AU12-08	544770801	--A--QL---AI-Q----L--G--LF		SM--H-----I--M-------SI--
	Gallionella capsiferriformans	302877537	--F--QL---DI---------LF-LY		-A-TS----FL--VF---A--S-F-
	Methylotenera sp. 1P/1	519026824	--F--QL---DI------L--FF-LN		N--VS-SS--I--M----I--SI--
	Pandoraea sp. B-6	515803985	--H--QL---I----------LC-L-		HW--A-PA--V--AT---S--AL--
	Candidatus Glomeribacter gigas	493732946	--LQ-HL---A--M----S--L--LE		AR--P-A---I--L----S--LIA-
	Candidatus Accumulibacter phos	257095697	--L--LL---SI---------LF-LF		-T--D--S--I--AS---S--L---
	Chitiniphilus shinanonensis	517578304	--A--YL---SI----V-L--LI-LM		---VP-Q---L--VF---S--SI--
	Thauera sp. MZ1T	217969594	--L--QL---DI------L--AF-L-		TT--E-P---I--M----A--A-F-
	Methylobacillus flagellatus KT	91776577	--L--HL---DI---------LF-LD		H--TDISS--I--A----V--S---
	Leeia oryzae	516893698	--L--ML---S-----M----LFSWH		---TS-GS-LL--AG---L--S---

Supplemental Figure 17: Partial sequence alignment of a conserved region of prepilin peptidase showing a 1 amino acid insertion (boxed) that is uniquely shared by the Clade IIa species.

Group	Species	Accession	191		230
Clade IIa *Burkholderia*	Burkholderia sp. SJ98	495619839	LWGAHAQAKRKLIDD	TQ	KAHCVLEAAHPSPLSAYRGFFGC
	Burkholderia sp. RPE64	507516982	----------G--EG	-G	--------V--------------
	Candidatus Burkholderia kirkii	494056224	----------G--EG	-A	---------------------S-
	Burkholderia sp. YI23	377821762	---------HA---G	-A	-S-----S---------------
Other *Burkholderia*	Burkholderia sp. JPY347	517251513	---S------V----		RP------P-------H---L--
	Burkholderia sp. Ch1-1	494324248	----------A-LGG		-S------P-------H---L--
	Burkholderia xenovorans LB400	91785323	----------A-LGG		-S------P-------H---L--
	Burkholderia phytofirmans PsJN	187925473	----------A-LGG		-S------P-------H---L--
	Burkholderia sp. H160	496198829	----------A-LGG		-S------P-------H---L--
	Burkholderia cepacia GG4	402567741	----------T-F-A		N-------P-------H---L--
	Burkholderia cenocepacia AU 10	107024121	----------A-F-A		N-------P-------H---L--
	Burkholderia multivorans	493457543	----------A-F-A		S-------P-------H---L--
	Burkholderia ubonensis	497778628	----------A-F-A		N-------P-------H---L--
	Burkholderia vietnamiensis G4	134294618	----------A-F-A		N-------P-------H---L--
	Burkholderia pyrrocinia	515903114	----------A-F-A		N-------P-------H---L--
	Burkholderia ambifaria	493810772	----------A-F-A		S-------P-------H---L--
	Burkholderia sp. TJI49	325523891	----------A-F-A		S-------P-------H---L--
	Burkholderia lata	78065092	----------A-F-A		S-------P-------H---L--
	Burkholderia oklahomensis	497802988	----------A-F-A		SE------P-------H---L--
	Burkholderia phymatum STM815	186477451	----------A-LEG		-S------P-------H---L--
	Burkholderia thailandensis MSM	488601778	----------A-F-P		RE------P-------H---L--
	Burkholderia dolosa	493817719	----------A-F-A		SE------P-------H---L--
	Burkholderia pseudomallei K962	53720664	----------A-F-A		RE------P-------H---L--
	Burkholderia mallei ATCC 23344	53717347	----------A-F-A		RE------P-------H---L--
	Burkholderia kururiensis	516386443	----------A-LAG		RP------P-------H---L--
Other Bacteria	Cupriavidus sp. BIS7	518278912	---S------A-L-G		QT------P-------H---L--
	Cupriavidus metallidurans CH34	94312114	---S------A-LEG		QT------P-------H---L--
	Cupriavidus sp. HMR-1	495922570	---S------A-LEG		QT------P-------H---L--
	Subdoligranulum sp. CAG:314	548249409	---GN-RR-KS-LTN		TS-L-------------N-----
	Taylorella equigenitalis 14/56	479196398	---GN-KT-S----K		SK-LI--SP--------------
	Neisseria polysaccharea	489848722	---GY--Q------S		QN-LI-T-P--------------
	Neisseria lactamica	489810458	---GY--Q------S		QN-LI-T-P--------------
	Oligella urethralis	516658460	---S---K-GRF--K		QK-L--T-P--------------
	Neisseria meningitidis	488162684	---GY--Q------S		QN-LI-T-P--------------

Supplemental Figure 18: Partial sequence alignment of a conserved region of uracil-DNA glycosylase showing a 2 amino acid insertion (boxed) that is uniquely shared by the Clade IIa species.

			221		270
Clade IIa *Burkholderia*	Burkholderia sp. YI23	377820970	KYFADIYALARLRLADLGVD	AA	RIHGGTHCTVTEKERFYSYRRDRVTGRM
	Burkholderia sp. SJ98	495624831	-----------M------I-	--	-V--------------------------
	Candidatus Burkholderia kirkii	494056945	------F----------D--	--	--Y----------A--------------
	Burkholderia sp. RPE64	507516101	---------------A----	T-	-M-----------Q--------------
Other *Burkholderia*	Burkholderia pseudomallei	497623000	--L-NL---------RV--T		-VS--AA--A--R---------------
	Burkholderia mallei NCTC 10247	126450684	--L-NL---------RV--T		-VS--AA--A--R---------------
	Burkholderia pyrrocinia	515901625	--L--LH-------VRA--S		-VS--GA-----PS--------------
	Burkholderia xenovorans LB400	91783471	--L--LP-------QA---T		--V--DL----LR---------------
	Burkholderia cenocepacia	493524638	-FL--L--------VRA--A		HVS---A---A-PA--------------
	Burkholderia kururiensis	516383731	--L-NLP-------NAI-IE		S-A--D-----DR-H-------------
	Burkholderia vietnamiensis G4	134295823	--L--LP----V---RA--V		-VS---A---A-RT--------------
	Burkholderia sp. JPY347	517252833	--L--L---------RH-IV		QVT--DA----QAD--------------
	Burkholderia phenoliruptrix BR	407713453	--L--LP-------QR---T		--A--DL----DR----------E----
	Burkholderia sp. CCGE1001	323526133	--L--LP-------QR---T		--A--DV----DR----------E----
	Burkholderia terrae	494860343	-FL--LPG---W---QI--T		NVA--D------R----------E----
	Burkholderia phytofirmans PsJN	187923832	--L--LP-------QR---T		--V--DL----QR----------E----
	Burkholderia graminis	492928437	--L--LP-------QR---T		-VV--DL---S-R----------E----
	Burkholderia sp. BT03	495016559	-FL--LPR---W---QI--T		NVT--D------R----------E----
	Burkholderia multivorans	493460484	--L--L-----V-V-RA--V		HVS--DA--L--RA--------------
	Burkholderia gladioli BSR3	330817147	-HH--L--------VR--IM		QAT---A-----RH-Y------------
Other Bacteria	Aquaspirillum serpens	550892691	--L----Q---Q--QAV--S		H-S--------DR---F-----GQ----
	Cupriavidus sp. HMR-1	495922251	--W--------I---RA-CV		-----EA----DAD--F------T----
	Oxalobacter formigenes	492547811	--L-------KMT-DE---T		DVS--GF----D----------G-----
	Chromobacterium violaceum ATCC	34497646	--L-----------RK---S		-VY--DF---IDRD--F-----G-----
	Oxalobacteraceae bacterium IMC	497350717	--L--L-Q----V--GQ--L		-VA------L--R-----F---GQ----
	Pseudogulbenkiania ferrooxidan	545115139	--L-----------EK---S		-VY--DF---IDRD--F------E----
	Cupriavidus basilensis	493138134	-----------I---RA-CT		AVY--DT---SDAQ--------G-----
	Neisseria cinerea	489771633	--L---------V-RRE---		M-Y-------L-RDT-F-----GM----
	beta proteobacterium L13	516079020	--L----L---Q---AA--S		AVY--DA---I-R---F-----Q-----
	Cupriavidus sp. WS	519045348	-----------T---RA-CL		DV---EA----DAQ--------G-----
	Cupriavidus taiwanensis LMG 19	194289465	-----L-----T---RA-CV		DV---DT---ADAD--------G-----
	Neisseria sp. GT4A_CT1	496669542	--L---------V-RRE---		M-Y-------L-RDT-F-----GQ----
	Herbaspirillum sp. JC206	517970905	-FL--L-L---M--NSA--A		KV---GF----DAR-------EKT----
	Janthinobacterium sp. Marseill	152981128	--L----H---MT--K---T		KVS--NA---SDPQ--------G-----
	Chitiniphilus shinanonensis	517578874	-WW--L-L---Q--VA---T		A----E----GDAA--F-----G-----

Supplemental Figure 19: Partial sequence alignment of a conserved region of hypothetical protein BYI23_A015260 showing a 2 amino acid insertion (boxed) that is uniquely shared by the Clade IIa species.

Group	Species	ID	321		362
Clade IIa *Burkholderia*	Burkholderia sp. YI23	377822128	HAEALGSQAALAEVAEIARG		TNDATWLRGVVDKEKSLHEAVR
	Burkholderia sp. SJ98	495627833	---------------D----		V---------------------
	Candidatus Burkholderia kirkii	494057772	-------E----QM-D----		----------------------
	Burkholderia sp. RPE64	507517331	------------Q--D----		M---------F-----------
Other *Burkholderia*	Burkholderia phytofirmans PsJN	187925899	-------GN----IGA----	Q	V----------ER---------
	Burkholderia phenoliruptrix BR	407715219	-GD----GH----IGA----	Q	I----------E----------
	Burkholderia sp. Ch1-1	494323506	-------GN----IGA---S	Q	V----------ER---------
	Burkholderia xenovorans LB400	91785771	-------GN----IGA---S	Q	V----------ER---------
	Burkholderia graminis	492933552	-GD----GH----IGA----	Q	I----------EQ---------
	Burkholderia sp. CCGE1001	323527875	-GD----GH----IGA----	Q	I----------E-------V--
	Burkholderia bryophila	518911520	-G---A-GH----IGT----	Q	V----------EE---------
	Burkholderia sp. WSM4176	517230069	-------VN-----GA----	Q	V-------S-FAQ---------
	Burkholderia sp. CCGE1002	295678169	--Q----VN-----GA----	Q	V-------S-FAQ---------
	Burkholderia kururiensis	516382242	--D----GH----IGM---S	Q	V----------AQ---------
	Burkholderia sp. JPY251	517243610	--Q----VN-----GA----	Q	V-------S-FAQ---------
	Burkholderia phymatum STM815	186477808	--D----GK--E-IGA----	Q	V-------N--EQ---------
	Burkholderia sp. H160	496198117	-------VN-----GA----	Q	V---K---S-FAQ---------
	Burkholderia sp. CCGE1003	307731493	-GD----GH----IGA----	Q	I---------AEQ------V--
	Burkholderia sp. JPY347	517252534	--Q--H-G---DQI-V---E	Q	M-------S--A----------
	Burkholderia glumae BGR1	238025722	--A----R---D-IGSL--A	R	V---S---T-FKQ----N-T--
	Burkholderia rhizoxinica HKI 4	312794862	--R----ID--TDIER----	G	E-------N-FEQ-Q--P-V--
	Burkholderia gladioli BSR3	330815010	--A----R---D--GAL-KA	R	V---S---T-FKQ----N-T--
	Burkholderia vietnamiensis G4	134294201	--A----R---D-IGAL-KA	R	V---S---T-FKQ----N-T--
	Burkholderia ambifaria AMMD	115350115	--A----R---D-IGAL-KA	R	V---S---T-FKQ----N-T--
	Burkholderia cenocepacia	493542467	--A----R---D-IGAL-KA	R	V---S---T-FKQ----N-T--
	Burkholderia pyrrocinia	515904175	--A----R---D-IGAL-KA	R	V---S---T-YKQ----N-T--
	Burkholderia sp. TJI49	325523603	--A----R---D-IGAL-KA	R	V---S---T-FKQ----N-T--
	Burkholderia oklahomensis	497803374	--A----R---D-IGAL-KA	R	V---S---TIFKQ----N-T--
	Burkholderia thailandensis MSM	488604890	--A----R---D-IGAL-KA	R	V---S---TIFKQ----N-T--
	Burkholderia dolosa	493815402	--A----R---D-IGAL-KA	R	V---S---TIFKQ----N-T--
	Burkholderia pseudomallei K962	53717640	--A----R---D-IGAL-KA	R	V---S---TIFKQ----N-T--
	Burkholderia cepacia GG4	402564874	--A----R---D-IGAL-KA	R	V---S---T-FKQ-R--N-T--
	Burkholderia lata	78064727	--A----R---D-IGAL-NA	R	V---S---T-FKQ----N-T--
	Burkholderia multivorans	493452796	--AE---R---D-IGAL-KA	R	V---S---T-FKQ----N-T--
	Burkholderia ubonensis	497778968	--AE---R---D-IGAL-QA	R	V---S---T-YKQ----N-T--
	Burkholderia sp. BT03	495009402	--RI--AEEG-DI-KRTLTE	G	VS--M---RIYGVHS----VA-
Other Bacteria	Pandoraea sp. B-6	515803113	--AV-EAE--CE--RQLVAS	G	K---V---D-HEEA---N-M--
	Cupriavidus sp. BIS7	518280190	-------LQ---NIRAL-AS	R	S---H-V-TIN-DAH--RDT--
	Ralstonia solanacearum GMI1000	17548015	-----A-EG--DM-R-VVER	R	DS--E-I-ATEGETRN---T--
	Pseudogulbenkiania ferrooxidan	545114825	--------HQ--ALRQR-LK	C	HS-SR---Q-FEQSG--S-V--
	Chromobacterium violaceum ATCC	34498359	--------HQ--ALRQR-LK	C	HS-SR---QIFEQSG--S-V--
	Ralstonia pickettii 12D	241664747	-----S-EG--DM-R-VVEA	R	DA--E-I-AAQRETRN---T--
	Cupriavidus taiwanensis LMG 19	194291156	-------LP---NIRAL-ER	R	DG--H---Q-DAEAR-QR-T--
	Hylemonella gracilis	493339361	--QSP-AES--KLLR-SVLQ	G	Q---R---E-QGR-QF-A-VS-
	Thiomonas sp. 3As	410694623	--ADCK-D---R-LLADVSA	R	G---E-I-E-FSR-HH-P-V--
	Variovorax paradoxus B4	538392801	-S---NATQ--G-LRTSVEA	N	R---R---EKQG--RL-A-V--
	Polaromonas sp. CF318	495147996	--T-M-AG-S-HLLRSSVER	G	D---R---ERQGE-HL-A-VI-

Supplemental Figure 20: Partial sequence alignment of a conserved region of carboxylate-amine ligase showing a 1 amino acid deletion (unboxed) that is uniquely shared by the Clade IIa species.

Group	Species	ID	303		348
Clade IIa *Burkholderia*	Candidatus Burkholderia kirkii	494056355	VAYSSIAHMGFVTLGFFMFG	ESS	QLAMEGGIIQMISHGFVSGAMFL
	Burkholderia sp. YI23	377821133	--------------------	P--	--G---A----------------
	Burkholderia sp. SJ98	495617133	--------------------	A--	---V--A----------------
	Burkholderia sp. RPE64	507516272	--------------------	P--	--GV--A----------------
Other *Burkholderia*	Burkholderia kururiensis	516384384	-----------------I-S		--GV--A-V--------------
	Burkholderia vietnamiensis	544733132	-----------------I-N		--GV--A----------------
	Burkholderia cenocepacia	493524210	-----------------I-N		--GV--A----------------
	Burkholderia ambifaria	493802584	-----------------I-N		--GV--A----------------
	Burkholderia multivorans	493450271	-----------------I-N		--GV--A-V--------------
	Burkholderia sp. TJI49	325524290	-----------------I-N		--GV--A-V--------------
	Burkholderia dolosa	493816260	-----------------I-N		--GV--A-V--------------
	Burkholderia xenovorans LB400	91782624	-----------------I-N		--GV--A-V--------------
	Burkholderia sp. CCGE1003	307730328	-----------------I-N		--GV--A-V--------------
	Burkholderia sp. CCGE1001	323525448	-----------------I-N		--GV--A-V--------------
	Burkholderia bryophila	518913716	-----------------I-N		--GV--A-V--------------
	Burkholderia lata	78067034	-----------------I-N		--GV--A----------------
	Burkholderia graminis	492940371	-----------------I-N		--GV--A-V--------------
	Burkholderia pyrrocinia	515901031	-----------------I-N		--GV--A----------------
	Burkholderia phenoliruptrix BR	407712819	-----------------I-N		--GV--A-V--------------
	Burkholderia thailandensis E26	83719938	-----------------I-N		--GV--A-V--------------
	Burkholderia ubonensis	497777019	-----------------I-N		--GV--A-V--------------
	Burkholderia pseudomallei K962	53718859	-----------------I-N		--GV--A-V--------------
	Burkholderia oklahomensis	497788347	-----------------I-N		--GV--A-V--------------
	Burkholderia phytofirmans PsJN	187923352	-----------------I-N		--GV--A-V--------------
	Burkholderia cepacia GG4	402565967	-----------------I-N		--GV--A----------------
	Burkholderia phymatum STM815	186476752	-----------------I-N		--GT--A-V--------------
	Burkholderia sp. JPY347	517254090	-----------------I-N		--GT--A-V--------------
	Burkholderia sp. WSM4176	517231204	-----------------I-N		--GA--A-V--------------
	Burkholderia sp. H160	496200756	-----------------I-N		--GA--A-V--------------
	Burkholderia sp. JPY251	517242675	-----------------I-N		--GA--A-V--------------
	Burkholderia sp. CCGE1002	295676049	-----------------I-N		--GA--A-V--------------
	Burkholderia rhizoxinica HKI 4	312796889	------T----------L-S		--GV--A-V--------------
	Burkholderia gladioli BSR3	330817780	-----------------I-S		--GI--A-V--------------
	Burkholderia glumae BGR1	238028131	-----------------I-S		P-GV--A-V--------------
Other Bacteria	Cupriavidus sp. HMR-1	495918782	-----------------I-N		EIGI-------------------
	Ralstonia solanacearum	489366002	-----------------I-N		EIGV----V-------I------
	Polynucleobacter necessarius s	171463538	-----V-----------L-S		P-GI----V--------A-----
	Thiomonas intermedia K12	296136694	-----------------L-S		D-GVQ--LV-------I------
	Ideonella sp. B508-1	551360979	-----------------I-N		E-G-S---V--------------
	Azoarcus sp. BH72	119897699	------S------------N		P--V--ALV--------------
	Lautropia mirabilis	491920002	-------------------N		ETG-Q-AVV--V-----A-----
	Janthinobacterium sp. Marseill	152980305	-------------------N		DM-VQ---V--------------
	Candidatus Glomeribacter gigas	493733119	-----------------L-D		A-----ALV--------------
	Thauera phenylacetica	490502749	------S------------N		S-GV--ALV--------------
	Thiomonas sp. FB-6	517743748	-----------------L-S		N-GV---LV-------------M
	Herbaspirillum sp. JC206	517971135	-----V-------------N		DMSVQ-A-V--------------
	Variovorax sp. CF313	495112919	-----V-----------I-N		E-GVS---V---A----------
	Methylotenera sp. 1P/1	519026470	I-----S------------S		--GL--AVV-------I-S----
	Janthinobacterium lividum	498085566	-------------------N		DISVQ---V--V----I------
	Dechlorosoma suillum PS	372489649	------------------LN		P-GV--ALV--------------
	Herminiimonas arsenicoxydans	134095019	-------------------N		DM-VQ---A--------------
	Leptothrix cholodnii SP-6	171058197	-----------------I-S		E-GIS--LV--------------
	Uliginosibacterium gangwonense	517435011	------S------------N		TIG---ALV---------A----
	Burkholderiales bacterium JOSH	497237631	-----------------I-N		E-GVS-ALV--V-----------
	Methylibium petroleiphilum PM1	124266608	-----------------I-N		E-GVS---V--------------

Supplemental Figure 21: Partial sequence alignment of a conserved region of NADH:ubiquinone oxidoreductase subunit M showing a 3 amino acid insertion (boxed) that is uniquely shared by the Clade IIa species.

Group	Species	Accession	538		585
Clade IIa *Burkholderia*	Candidatus Burkholderia kirkii	494056354	HEGLKEMAEEFHGWVAMALHGI	I	TPGPVWLAALGVIVAWFFYMKRPDL
	Burkholderia sp. RPE64	507516273	----------------------	-	-------------------------
	Burkholderia sp. SJ98	495617135	--------------I-------	-	-------------------------
	Burkholderia sp. YI23	377821134	----------------------	-	-------------------------
Other *Burkholderia*	Burkholderia rhizoxinica HKI 4	312796890	-DA-R---G-----MP----SL		SSL-I---LV-------L-L-----
	Burkholderia pseudomallei	490691823	-PA-A---------T--G--SV		SGL-----LA--V----L-L-----
	Burkholderia thailandensis E26	83720737	-PA-A---------T--G--SV		SGL-----LA--V----L-L-----
	Burkholderia dolosa	493816259	-PA-A----------G-G--SV		SGL-----LA--V----L-L-----
	Burkholderia vietnamiensis G4	134296422	-PA-A----------G-G--SV		SGL-----LA--V----L-L-----
	Burkholderia multivorans	493460316	-PA-A----------G-G--SV		SGL-----LA--V----L-L-----
	Burkholderia glumae	515992959	-PAVA---D-----A--G--SV		-GL-----LA--V----L-L-----
	Burkholderia mallei ATCC 23344	53725804	-PA-A---------T--G--SV		SGL-M---LA--V----L-L-----
	Burkholderia sp. JPY347	517254091	-PA-H---------L--G--SV		SGL-----LA--VT---L-L-----
	Burkholderia oklahomensis	497788345	-PA-A---------A-LG--S-		STL-L---LA--VT---L-L-----
	Burkholderia ambifaria AMMD	115352327	-PA-T----------G-G--SV		SGL-----LA--V----L-L---E-
	Burkholderia cepacia GG4	402565966	-PA-A----------G-G--SV		SGL-----LA--V----L-L---E-
	Burkholderia cenocepacia J2315	206560692	-PA-A----------G-G--SV		SGL-----LA--V----L-L---E-
	Burkholderia lata	78067035	-PA-A----------G-G--SV		SGL-----LA--V----L-L---E-
	Burkholderia kururiensis	516384383	-PA-A---------LDLG--AV		-GL-M---LA--V----L-L-----
	Burkholderia gladioli BSR3	330817781	-PA-A---D-----A--G--SV		SGL-A---LA--V----L-L-----
	Burkholderia sp. CCGE1003	307730329	-PA-H---------A--G--SV		-GL-----LA--L----L-LL--E-
	Burkholderia sp. CCGE1002	295676048	-PA-R------Q--AS-G--SV		SGL-----LA--V----L-LV----
	Burkholderia pyrrocinia	515901032	-P--E-LGK-----A--G--SL		-TL-L---IA--AT---L-L---E-
	Burkholderia ubonensis	497777017	-P--E-LGK-----A--G--SL		-TL-L---IA--AT---L-L---E-
	Burkholderia phenoliruptrix BR	407712818	-PA-H---------A--G--SV		AGL-----LA--V----L-LV--E-
	Burkholderia sp. CCGE1001	323525447	-PA-H---------A--G--SV		AGL-----LA--V----L-LV--E-
	Burkholderia bryophila	518913715	-PA-H---------A--G--SV		AGL-----LA--V----L-LV--E-
	Burkholderia sp. BT03	495017894	-PA-H------Q--AS-G--AA		SGL-----LA--V-S--L-L-----
	Burkholderia graminis	492940374	-PA-H---------A--G--SV		AGL-----LA--L----L-LV--E-
	Burkholderia terrae	494862035	-PA-H------Q--AS-G--AA		SGL-----LA--V-S--L-L-----
	Burkholderia sp. H160	496200757	-PA-H------Q--AS-G--SV		SGL-----LA--L----L-LV----
	Burkholderia phymatum STM815	186476753	-PA-H------Q--AS-G--AA		SGL-A---LA--V----L-L-----
	Burkholderia phytofirmans PsJN	187923351	-PA-H------Q--AS-G--SV		AGL-----LA--V----L-LV----
	Burkholderia sp. Ch1-1	494312570	-PA-H------Q--AS-G--SV		AGL-----LA--V----L-LI----
	Burkholderia xenovorans LB400	91782623	-PA-H------Q--AS-G--SV		AGL-----LA--V----L-LI----
	Burkholderia sp. WSM4176	517231203	-PA-H--T---Q--AS-G--SV		SGL-----LA--V----L-LV----
Other Bacteria	Cupriavidus sp. WS	519047766	-HAME-L--A-------G--SL		-TPVL---IA--VLS---------I
	Ralstonia sp. GA3-3	498508643	-HAMA-LK-A--------I-SL		-TPVL---VA--VLS---------I
	Azoarcus toluclasticus	517822020	-V--S-L--H---P-------L		QTA-F---MG--VL------V---I
	Thauera sp. 28	489015980	-V----LEAS---P----I--L		QTA-F---IG--ALS---------I
	Aromatoleum aromaticum EbN1	56478187	-V--S-L--N---P----V--L		QTA-F---MG--GL------V---I
	Pandoraea sp. SD6-2	498506742	-PAME-LSH-----------SF		GTL-IA-S---IVL-A---L----I
	Pseudogulbenkiania sp. NH8B	347538450	-P-MH-L------P----V-AL		STL-F---LS--V------L-A-QI
	Chromobacterium violaceum ATCC	34496407	-P-ME-L-H----AA--T--SF		-TL-L---IA--V---Y----A-HI
	Dechloromonas aromatica RCB	71906599	-PAMEHL------A---GM-SF		-SL-FI--LS--V-S---------I
	Methylobacillus flagellatus KT	91776402	-GAMR-L-HH---AW------L		-SL-FI---S--AL----------I
	Comamonas testosteroni	489148084	-GAMA-L--QI----P-----F		-AA-F---LA--V-SYV---VK-EI

Supplemental Figure 22: Partial sequence alignment of a conserved region of NADH:ubiquinone oxidoreductase subunit L showing a 1 amino acid insertion (boxed) that is uniquely shared by the Clade IIa species.

Group	Species	Accession	59		99
Clade IIa *Burkholderia*	Burkholderia sp. YI23	377821271	GRIVANGIDVTHQSPQRVSR		TLARSFQTTSVFGAMSVLDNL
	Burkholderia sp. RPE64	507516423	-----G-----GKP------		-------------GL------
	Burkholderia sp. SJ98	495617403	-----S---I-GKP------		S------------GL--FE--
	Candidatus Burkholderia kirkii	494057657	-----D-T-ING-P-P----		V-------------L------
Other *Burkholderia*	Burkholderia kururiensis	516388040	---EL-SV-I-RLT-A--NH	L	G------------RL--Y---
	Burkholderia pseudomallei	497656514	---AL---ELGRRR-HA-A-	L	G------Q----AR---Y---
	Burkholderia thailandensis MSM	488602751	---AL--VELGRRP-HA-A-	L	G------Q----AR-T-----
	Burkholderia mallei	490310235	---AL---ELGRRR-HA-A-	L	G------Q----AR---Y---
	Burkholderia phenoliruptrix BR	407714229	---DLF-AEI-RLA-AAIA-	R	G------S-----RL--F---
	Burkholderia sp. CCGE1002	295677098	---HLF-A-I-RLA-AAIA-	R	G------S----ARL--F---
	Burkholderia sp. JPY251	517244669	---HLF-A-I-RLA-AAIA-	R	G------S----ARL--F---
	Burkholderia phymatum STM815	186476363	---ELY-A-I-RLA-AAIC-	R	G--------RF-AKL------
	Burkholderia sp. CCGE1003	307730450	---HLF-AEI-RLA-AAIT-	R	G------S----ARL--F---
	Burkholderia sp. WSM4176	517232379	---HLF-A-I-RLA-AAIA-	R	GV-----S----ARL--F---
	Burkholderia xenovorans LB400	91784479	---DLF-AEI-RLA-TAIA-	R	G------S----ARL--F---
	Burkholderia graminis	492938594	---DLF-AEI-RLA-AAIA-	R	G------S----ARLT-F---
	Burkholderia phytofirmans PsJN	187924791	---DLF-AEI-RLA-AAIT-	R	G------S----TRL--F---
	Burkholderia sp. Ch1-1	494313820	-K--FD-H----EPDHKRV-	R	GV-----V--L-ANL--RE--
	Burkholderia sp. CCGE1001	323526784	---DLF-AEI-RLA-AAIA-	R	G------S----ARL--F---
	Burkholderia sp. JPY347	517250143	--VLLA-R---GERSHELA-	M	G-G------RI-ASLT-F---
	Burkholderia bryophila	518915499	-T--FD-H----EPDHKRV-	R	GV-----V--L-ANL--QE--
	Burkholderia vietnamiensis	544732673	--V-LH-V-LRGRG-VVA--	L	GIG----Q--A-ARLT-F---
	Burkholderia pyrrocinia	515901928	--V-LH-V-LRGRG-VAA--	L	GIG----Q--A-ARLT-F---
	Burkholderia cenocepacia J2315	206559790	--V-LH-VELRGRG-VVA--	L	GIG----Q----ARLT-F---
	Burkholderia cepacia GG4	402566780	--V-LH-VELRGRG-VVA--	L	GIG----Q--A-ARL--F---
	Burkholderia ambifaria MC40-6	172060387	--V-LH-VELRGRG-VVA--	L	GIG----Q--A-ARL--F---
	Burkholderia sp. TJI49	325519808	--VTFD-R---GAA-E-LAA	L	G---T--HGR---NL-----V
	Burkholderia sp. H160	496197676	----FE-S-I--LT-HKRA-	L	G------IL---PNL-AFE-V
Other Bacteria	Herbaspirillum seropedicae SmR	300311565	---TLH-V-I-R----QIN-	L	G------I--L-PRL-AFE-V
	Herbaspirillum sp. YR522	495394181	---LLH-Q-I-RA---AIN-	L	G------LS-L-PRL-A---V
	Herbaspirillum sp. GW103	495604000	--VLLH-R---REP---IH-	L	G------I--L-PRL-AFE-V
	Herbaspirillum frisingense	493763978	--VLLH-Q-I-QC---QIN-	L	G------I--L-PRLTAFE-V
	Limnohabitans sp. Rim47	518258794	-D-LL--QRIQ-KT-YEIN-	M	G------I-NI-PKL--FE--
	Pandoraea sp. B-6	515804033	--VLM-TS---GRASHVI--	L	G-S-----SQL--Q---I-H-
	Ramlibacter tataouinensis TTB3	337278640	-T-TY--V-I--EK-AQTA-	R	GIV----ISA--PH-T--E-V
	Bordetella petrii DSM 12804	163858813	----FD-V---DVQ--DRVA	M	GM--T--I-E--RELT-RE--
	Limnohabitans sp. Rim28	518255821	--MTLS-T-I-GWT-EKI--	H	G-G--Y-K-NI--TFT-W--V
	Acidovorax sp. CF316	495129171	----FD-H----EPDH-RVQ	R	GM-----V--L-LTLP-RE--
	Cupriavidus metallidurans CH34	94313403	----FE-K---AEADYQRVQ	R	GI-----V--L-PNL--RE--
	Cupriavidus taiwanensis LMG 19	194292063	----FD-K---AEADYQRVQ	R	GI-----V--L-PSLP-RE--
	Ralstonia pickettii DTP0602	549450724	----FD-K---AETDYQRVQ	R	GI-----V--L-PSLP-RE--
	Bordetella sp. FB-8	518782028	----FD-A---DVP--NRVA	M	GM--T--I-E--REL--RE--
	Pandoraea sp. SD6-2	498507416	--VLLHAR---GRA-HVI--	L	G-S-----SQL-LQ---I-H-
	Curvibacter lanceolatus	518405125	---TLG-Q---GW-AE-I--	Q	G-G--Y-K-NI-APL--W--I
	Polaromonas sp. JS666	91789914	-T-TY--T-I--EQ-AQTA-	R	GIV----ISA--PH-T--E-V
	Cupriavidus sp. HPC(L)	555415592	----FD-H---GETDYQRVQ	R	GI-----V--L-PSLP-RE--
	Achromobacter xylosoxidans A8	311107607	-S--FD-Q---QEADFQRVQ	R	GI-----V--L-ANL--RE--
	Thiomonas sp. FB-6	517745025	-S--FD-R-I-RMQ-AQ-A-	L	G-V----ISAT--H--L---V
	Rubrivivax benzoatilyticus	497541661	----F--H-I-GE--AHIA-	R	GVI----ISA--PHLT--E-V
	Ralstonia sp. AU12-08	544767950	-T--FD-H---READYQRVQ	R	GI-----V--L-PSL--RE--
	Janthinobacterium sp. Marseill	152979977	-D-LL--SSIAG-K-YQIN-	R	G-S----I-NI-HRL--FE--
	Curvibacter putative symbiont	260222937	-K-EL--Q--S-SG-LELN-	R	GVG-T---LQ---K-T-R---

Supplemental Figure 23: Partial sequence alignment of a conserved region of ABC transporter showing a 1 amino acid deletion (unboxed) that is uniquely shared by the Clade IIa species.

Group	Species	Accession	133		172
Clade IIa *Burkholderia*	Burkholderia sp. YI23	377819666	VWLRTDDKPVADVIKTAYP	QP	DTRMLVADEKAVAAWLPVD
	Burkholderia sp. SJ98	495627170	---------------S---	--	-----------I------N
	Candidatus Burkholderia kirkii	494056850	----------S--------	--	---L-----Q---------
	Burkholderia sp. RPE64	507514747	----------S--L-----	--	-----------I-----AG
Other *Burkholderia*	Burkholderia graminis	492926693	------AGN-----QK---		--N--I--PAQ-S------
	Burkholderia sp. CCGE1001	323524647	------AGK-----QK---		--D-----PAQ-S------
	Burkholderia phenoliruptrix BR	407712025	------AGK-----QK---		--D-----PAQ-S------
	Burkholderia sp. JPY347	517254804	---L--AGA-P----G---		--------PA-I-----AE
	Burkholderia sp. WSM4176	517230495	------ADK-----Q----		N-D--I--PAQ------A-
	Burkholderia sp. CCGE1002	295675371	------ADK-----Q----		N-D--I--PAQ------A-
	Burkholderia sp. H160	496202032	------ADK-----Q----		N-D--I--PAQ------A-
	Burkholderia sp. JPY251	517245898	------ADK----VQ----		N-D--I--PAQ------A-
	Burkholderia xenovorans LB400	91781682	------AGN-----QK---		--N-MI--PAQ------TE
	Burkholderia sp. CCGE1003	307728357	------AGN-P---QK---		--N--I--LAQ-S------
	Burkholderia sp. Ch1-1	494322120	------AGN-----QK---		--N-MI--PAQ-S----TE
	Burkholderia kururiensis	516382574	------TQQ-PPAVQG--D		--E--I--PS--S------
	Burkholderia phymatum STM815	186475053	-F----PGK-P-IVDN--K		--E--I--P-E------AE
	Burkholderia phytofirmans PsJN	187922559	------AGN----VQK---		--N-MI--PAQ-S----TA
	Burkholderia bryophila	518911150	-----GAGN-----QK---		--N--L--PAQ-S----TA
	Burkholderia pyrrocinia	515903726	----S-AGQ-PEKVSA---		---R----PA-------A-
	Burkholderia ubonensis	497776425	----S-AGA-P-K-TA---		---R----PA---R---A-
	Burkholderia sp. TJI49	325525371	----S-AGA-PQKVLD---		---R----PA---------
	Burkholderia cenocepacia	493545202	----S-AGA-PQKVLD---		---R----PA---------
	Burkholderia multivorans	493461626	----S-TGA-PQKVLD---		---R----PA-------A-
	Burkholderia ambifaria MC40-6	172061797	----S-AGA-PPKVLE---		---R----PA-------A-
	Burkholderia dolosa	493815739	----S-AGAIPQKVLD---		---R----PA-------A-
	Burkholderia cepacia GG4	402565399	----S-AGA-PPKVLE---		---R----PA-------A-
	Burkholderia vietnamiensis G4	134297030	----S-AGAIPPKVLE---		---R----PA-------A-
	Burkholderia sp. KJ006	387903350	----S-AGAIPPKVLE---		---R----PA-------A-
	Burkholderia lata	78067638	----S-AGAMPQKVLD---		---R----PA-------A-
	Burkholderia rhizoxinica HKI 4	312797384	-------GAIPERVAN---		---K-IVPPAQ------P-
	Burkholderia thailandensis MSM	488604679	---KS-AAN-PAA-AD---		---K-R--PA-------A-
	Burkholderia pseudomallei MSHR	237810701	---KS-AAN-PAA-AD---		---K-R--PA-------A-
	Burkholderia mallei ATCC 23344	53724511	---KS-AAN-PAA-AD---		---K-R--PA-------A-
	Burkholderia gladioli BSR3	330818384	----S-AGS-DAKLLA---		---RFIV-PA--E----A-
	Burkholderia oklahomensis	497787276	---KS-AAN-PAA-AD---		--VR-R--PA-------A-
	Burkholderia glumae	515993317	----R-AAAIDARLLA---		---R----PA-LD----AE
Other Bacteria	Pandoraea sp. SD6-2	498504092	L--V---A--SGKLDA--E		---R-RT-PV-L-S---TQ
	Pandoraea sp. B-6	515801926	L--V--AAS-PQK-DS---		---R-RV-PA-L-----TQ
	Herminiimonas arsenicoxydans	134096071	---I-----LDTILIREFD		G-H--RVKSE--R-----E
	Janthinobacterium sp. Marseill	152982287	---I------DTILIREFD		G-D--RVK-EL-R----T-
	Herbaspirillum sp. CF444	495154775	---I--A--LDT-LMRE-D		G-D--RV-A-LLK----T-

Supplemental Figure 24: Partial sequence alignment of a conserved region of hypothetical protein BYI23_A002220 showing a 2 amino acid insertion (boxed) that is uniquely shared by the Clade IIa species.

Group	Species	Accession	147		201
Clade IIa *Burkholderia*	Candidatus Burkholderia kirkii	494056031	RDAALALKGHTVNIARSEFPKLD	S	EDEFYWVDLIGLDVENEAGVALGRVADLIDN
	Burkholderia sp. SJ98	495617762	----------------GD-----	-	D------------------------------
	Burkholderia sp. RPE64	507516575	--T-A-----A-YV--GD-----	-	-------------------------------
	Burkholderia sp. YI23	377821406	-N--E----YA-H----D-----	-	---------------S---------------
Other *Burkholderia*	Burkholderia phymatum STM815	186475535	-------R--R-YVR-RD--A-G		T--Y-----V----V----IE--K-------
	Burkholderia oklahomensis	497802265	-------R-FR-FVR-GD--A-A		D-------------V--------KI--M---
	Burkholderia gladioli BSR3	330815921	--Q----R-YR-YVR-ED--A--		A-------------V-----E----G-M---
	Burkholderia graminis	492931826	-------R-SR-YVS-----A-E		A--------L----V-L---N--K---M---
	Burkholderia bryophila	518913457	-----S-R-SR-Y-S-----A-E		A--------L----V-L---N--K---M---
	Burkholderia xenovorans LB400	91784786	--V----R-SR-Y-S-----A-G		A--------L----V-V---S--K---M---
	Burkholderia glumae BGR1	238026560	--Q----R-YR-FVR-ED--A--		T-----------E-V----T-----G-M---
	Burkholderia thailandensis	497590230	--T----R-FR-FVR-GD--A-A		A-------------V--------K---M---
	Burkholderia kururiensis	516384574	-------R--R-YVS-T---A-E		A--------L--E-V--G-EV--K--SM---
	Burkholderia sp. JPY347	517252696	--D--G-R-YR-HVS-AD--A-G		---------VD-E-V----------------
	Burkholderia pseudomallei K962	53720100	--T----R-AR-FVR-GD--A-A		A-------------V--------KI--M---
	Burkholderia mallei ATCC 23344	53724763	--T----R-AR-FVR-GD--A-A		A-------------V--------KI--M---
	Burkholderia phytofirmans PsJN	187924952	--V----R-SR-Y-S-----A-G		A--------L----V-V---N--K---M---
	Burkholderia sp. H160	496203658	--V----R-SR-Y-S-----A-E		A-------------V-L--LN--K--SM---
	Burkholderia sp. WSM4176	517232527	--T----R-SR-Y-G-----A-E		A-------------V-L--LN--K--SM---
	Burkholderia sp. Ch1-1	494326081	--V----R-SR-Y-S-----A-G		V--------L----V-V---N--K---MV--
	Burkholderia phenoliruptrix BR	407714369	-------R-SR-Y-S-----A-E		A-----------E-V-L--AN--K--GM---
	Burkholderia sp. CCGE1001	323526928	-------R-SR-Y-S-----A-E		A-----------E-V-L--AN--K--GM---
	Burkholderia sp. JPY251	517244546	--V--G-R-SR-Y-S-----A-E		A-------------V-L--LN--K--SM---
	Burkholderia ubonensis	497777586	-------R-FR-SVR-ED--A--		A-------------V--QS----K-IGM---
	Burkholderia sp. CCGE1002	295677252	--V--G-R-SR-Y-S-----A-E		A-------------V-L--LN--K--SMV--
	Burkholderia sp. CCGE1003	307730594	-------R-SR-YVS-----A-Q		A--------L----V-L--EN--K--GM---
	Burkholderia vietnamiensis	544729944	------MR-FR-FVR-ED--A-A		A-------------V--QS----K-SGM---
	Burkholderia lata	78065656	----F-MR-FR-FVR-ED--A-A		A-------------V--QS----K-SGM---
	Burkholderia sp. TJI49	325524025	-------R-FR-FVR-ED--A-A		A-----------E-V--QS----K--GM---
	Burkholderia ambifaria MC40-6	172060005	-------R-FR-FVR-ED--E-A		A-----------E-V--QS----T-SGM---
	Burkholderia dolosa	493817268	-------R-FR-FVR-ED--A-A		A-------------V-QQS----Q-IGM---
	Burkholderia pyrrocinia	515902528	-----S-R-FR-FVR-ED--A-A		I-------------V--QS----K-IGM---
	Burkholderia cenocepacia AU 10	107022148	-----SMR-FR-FVR-ED--A-A		A-----------E-V--QS----K-SGM---
	Burkholderia cepacia GG4	402567206	-------R-FR-FVR-ED--A-A		A-----------E-V--QS----K-SGM---
	Burkholderia multivorans	493445332	-------R-FR-FVR-ED--A-A		A-------------V--QSAT--K-VGM---
	Burkholderia rhizoxinica HKI 4	312795478	--Q-F--R-A--HVR-AD--A-E		R--Y--------A-V--F-D---Q-VA-M--
Other Bacteria	Herminiimonas arsenicoxydans	134093875	----E----A--Q-P--H--A-S		DN----------E---LQ--R--Q-S-MM--
	Janthinobacterium sp. Marseill	152980656	----E----A--Q-P--H--A-S		DN----------E---LQ-EH--Q-S-MM--
	Herbaspirillum lusitanum	515279582	----E----AV-Q----H--A-S		DG----------A---MQ-ES--V-S--M--
	Massilia niastensis	522141247	-NE-E----AS-QV------E-E		---Y--S-------V-LQ-E---K---MMH-
	Collimonas fungivorans Ter331	340786358	----E-M--SV-H-P--H--A-A		D----------MM---LQ-EQ--V----M--
	Massilia timonae	491915131	-NE-E----AS-QV------A-E		---Y--S-------V-LQ-E---K-T-MMH-
	Oxalobacteraceae bacterium AB_	522197945	--M-E----AA-SVP--R--A-S		D-----TE--------LQ-EH--T-T-MMS-
	Duganella zoogloeoides	518764419	-NV-E----AA-S-P--R--V-S		------TE----E---LQ-ES--T-T-MMS-
	Janthinobacterium sp. CG3	516490305	----E----AA-QVP--H--T-S		A-----S-----T-Q-LQ-EC--Q---MMS-
	Oxalobacter formigenes	492547899	-E--E--R-SVIC-S--R--E-P		---Y--------S-F-LR-D---T-RG----
	Acidovorax radicis	498149350	----E--R-ARIFVP--S--STT		---Y--------E-V-RE-----Q-QE-MST
	Rubrivivax gelatinosus IL144	383757203	--I-Q--A-ARIFV---S--TP-		-------------AR-R-D-V---IVG-VET
	Janthinobacterium lividum	498081369	----E----VS-Q-P--H--T-T		A-----S-----T---LQ-EC--T-H-MMS-
	Burkholderiales bacterium JOSH	497236897	-N--EG---AR-FVS-TA--TP-		-G----I-------V-RD-A-----VG--ET
	Cupriavidus taiwanensis LMG 19	194288940	-NL-E--R-RR-W-R-AD--AP-		-----------CN-S--Q-EL--E-SG----
	Methylibium petroleiphilum PM1	124266297	-N--E--R-CS-FVS-AS--TA-		V--Y--I-----A-V-RE-Q---N----L-T
	Ralstonia eutropha H16	113866920	-NL-E----RR-W-R-AD--APE		-----------CT-S--Q-EL--E-SG----
	Thiomonas sp. FB-6	517743243	--Q-E----WSIHLR-ED--PPE		G----------CE-C-RE-A-----IG-L-S
	Janthinobacterium sp. HH01	495721440	-NV-E----AA-SVP--R--A-S		D-----AE------V-LQ-EH--T-T-MMS-
	Verminephrobacter aporrectodea	497790553	----DS-R-ARIFV---G--SPA		Q--Y--------Q-S-RE-----Q-RE-LST

Supplemental Figure 25: Partial sequence alignment of a conserved region of 16S rRNA-processing protein RimM showing a 1 amino acid insertion (boxed) that is uniquely shared by the Clade IIa species.

Group	Species	ID	106		144
Clade IIa *Burkholderia*	Burkholderia sp. YI23	377819737	LAEIEKRRSADLSAHSDEAG	S	RERAGRVQLLIAAARRAV
	Burkholderia sp. RPE64	507514825	------------T-------	-	------------------
	Burkholderia sp. SJ98	495627055	------------T-------	-	----E-----V-------
	Candidatus Burkholderia kirkii	494057640	----------------E---	-	----E-------------
Other *Burkholderia*	Burkholderia thailandensis	497597074	-S------R----E-G-T--		----A--EM-A---Q---
	Burkholderia pseudomallei K962	53718277	-S------R----E-G-A--		----A--EM-A---Q---
	Burkholderia mallei ATCC 23344	53724988	-S------R----E-G-A--		----A--EM-A---Q---
	Burkholderia sp. CCGE1001	323524731	-S------R---TE-G----		V---A--ET-VQ------
	Burkholderia phenoliruptrix BR	407712106	-S------R---TE-G----		V---A--ET-VQ------
	Burkholderia ubonensis	497776519	-G------R---T--Q-D--		----V--GM-E---Q---
	Burkholderia sp. JPY251	517243135	--------R----E-G----		VD--A--EK-VQ-----I
	Burkholderia sp. CCGE1002	295675459	--------R----E-G----		VD--A--EK-VQ-----I
	Burkholderia kururiensis	516382652	--------R---QE------		ID--A--ET-VG-T---I
	Burkholderia sp. KJ006	387903244	-T--G---R---TE-R-D--		-------EM-E---Q---
	Burkholderia vietnamiensis G4	134296932	-T--G---R---TE-R-D--		-------EM-E---Q---
	Burkholderia sp. Ch1-1	494321969	-S------R---TE-G----		VD--A--ET-VQ------
	Burkholderia gladioli BSR3	330818299	----D---Q----E-G--P-		----S--AM-TV---G--
	Burkholderia xenovorans LB400	91781792	-S------R---TE-G----		VD--A--ET-VQ------
	Burkholderia phytofirmans PsJN	187922653	-S------R---TE-G----		VD--A--ET-VQ------
	Burkholderia bryophila	518913746	-S------R---TE-G----		VD--A--ET-VQ------
	Burkholderia sp. CCGE1003	307728442	-S------R---TE-G----		VD--A--ET-VQ------
	Burkholderia graminis	492926510	-S------R---TE-G----		VD--A--ET-VQ------
	Burkholderia multivorans	493455224	-T--G---R---TE-H----		----A--EM-E---Q---
	Burkholderia ambifaria AMMD	115352837	-T--G---R---T--H-D--		----E--EM-E---Q---
	Burkholderia oklahomensis	497787533	-S------R----E-G-V--		---SA--EM-AS--Q---
	Burkholderia cepacia GG4	402565504	-T--G---R---T--H-D--		----V--EM-E---Q---
	Burkholderia pyrrocinia	515903605	-G------R---T--Q-D--		----V--GA-E---Q---
	Burkholderia sp. WSM4176	517230573	--------R---RE-G----		VD--A--EK-VQ-----I
	Burkholderia sp. H160	496203923	--------R---RE-G----		VD--A--EK-VQ-----I
	Burkholderia glumae BGR1	238027885	----D---Q----T-D-D--		HD--S--A--TV---G--
	Burkholderia cenocepacia	493543531	-T--G-------TE-R-D--		----S--EM-ET--Q---
	Burkholderia lata	78067531	-T--G-------TE-R-D--		----S--EM-ET--Q---
	Burkholderia rhizoxinica HKI 4	312797310	-N--D---H---RE-G-D--		HG--A--GS-VT---G--
	Burkholderia dolosa	493815820	-T--G---H---TE-H-D--		-G--A--EM-E---Q---
	Burkholderia sp. TJI49	325523404	-T--G---R---TE-H-D--		----V--EA-E---Q---
Other Bacteria	Ralstonia solanacearum	489362762	--------T--QT--N-PE-		D---R--EQ-V-H-----
	Pandoraea sp. SD6-2	498504375	-N--D---R--VAE-A-AT-		----A--EQ-E-------
	Cupriavidus sp. HPC(L)	493626750	--------A--RAE-AEP-A		ED-SH--EQ-VTF--Q-I

Supplemental Figure 26: Partial sequence alignment of a conserved region of FAD linked oxidase domain-containing protein showing a 1 amino acid insertion (boxed) that is uniquely shared by the Clade IIa species.

Group	Species	Accession	306		341
Clade IIa *Burkholderia*	Burkholderia sp. SJ98	495626933	DAVPPGDEAFKQGD		RIVLLRKQVIFTGDRIIDASAG
	Burkholderia sp. YI23	377819803	--------------		----------------V-----
	Candidatus Burkholderia kirkii	494057159	E-------------		-T------L-------------
	Burkholderia sp. RPE64	507514903	E-----------N-		-T--------------------
Other *Burkholderia*	Burkholderia sp. WSM4176	517230633	-P------L-T--N	Q	TP--------------------
	Burkholderia sp. H160	496202649	-P------L-T--N	Q	AP--------------------
	Burkholderia sp. CCGE1002	295675562	-P------L-T--N	Q	AP--------------------
	Burkholderia sp. JPY251	517243084	-P------L-T--N	Q	AP--------------------
	Burkholderia glumae BGR1	238026272	-P------L-T--N	Q	VP--------------------
	Burkholderia thailandensis	518085509	-P----E-L-T--N	Q	VP---K----------------
	Burkholderia phenoliruptrix BR	407712162	-P------L-T--N	Q	TP------I-------------
	Burkholderia phytofirmans PsJN	187922707	-P------L-T--N	Q	TP------I-------------
	Burkholderia xenovorans LB400	91781853	-P------L-T--N	Q	TP------I-------------
	Burkholderia sp. CCGE1001	323524787	-P------L-T--N	Q	TP------I-------------
	Burkholderia sp. Ch1-1	494321824	-P------L-T--N	Q	TP------I-------------
	Burkholderia sp. CCGE1003	307728502	-P------L-T--N	Q	TP------I-------------
	Burkholderia graminis	492926384	-P------L-T--N	Q	TP------I-------------
	Burkholderia pseudomallei	497666717	-P----E-L-T--N	Q	AP---K----------------
	Burkholderia oklahomensis	497802852	-P----E-L-T--N	Q	AP---K----------------
	Burkholderia mallei ATCC 23344	53726172	-P----E-L-T--N	Q	AP---K----------------
	Burkholderia sp. TJI49	325523863	-P----E-L-T--N	Q	VP---K-D--------------
	Burkholderia dolosa	493817558	-P----E-L-T--N	Q	VP---K-D--------------
	Burkholderia multivorans	493455086	-P----E-L-T--N	Q	VP---K-D--------------
	Burkholderia ambifaria MC40-6	172059696	-P----E-L-T--N	Q	AP---K-E--------------
	Burkholderia cepacia GG4	402567554	-P----E-L-T--N	Q	AP---K-E--------------
	Burkholderia vietnamiensis	544734174	-P----E-L-T--N	Q	AP---K-E--------------
	Burkholderia lata	78065284	-P----E-L-T--N	Q	AP---K-E--------------
	Burkholderia gladioli BSR3	330815587	-P------L-T--N	Q	AP-----S--------------
	Burkholderia kururiensis	516387304	AP------L-T--N	Q	AP--------------------
	Burkholderia ubonensis	497777212	-P----E-L-T--N	Q	AP---K-D--------------
	Burkholderia cenocepacia AU 10	107021796	-P----E-L-T--N	Q	AP---K-D--------------
	Burkholderia bryophila	518913803	-P------L-TH-N	Q	TP---K--I-------------
	Burkholderia sp. JPY347	522054903	EP------L-TE-N	Q	VP------------S-------
	Burkholderia pyrrocinia	515902827	-P----E-L-T--N	Q	AP---K-D--------------
	Burkholderia phymatum STM815	186477275	-P--A---L-T--N	A	VP-----S--------------
	Burkholderia terrae	494852539	-P--A---L-T--N	A	AP-----S--------------
	Burkholderia sp. BT03	495014255	-P--A---L-T--N	A	AP-----S--------------
	Burkholderia rhizoxinica HKI 4	312795157	-P--V--DL-T--N	G	AP-Y-K----------T-----
Other Bacteria	Cupriavidus metallidurans CH34	335055638	-P--F---L-T--N	S	AP--------------ES----
	Ralstonia pickettii	490770609	-P--L---L-T--R	S	AP---Q----------TS----
	Candidatus Glomeribacter gigas	493733377	-P------L-T--R	D	APA--K-DL--S-E--V--G—

Supplemental Figure 27: Partial sequence alignment of a conserved region of preprotein translocase subunit SecD showing a 1 amino acid deletion (unboxed) that is uniquely shared by the Clade IIa species.

			101		143
Clade IIa *Burkholderia*	Candidatus Burkholderia kirkii	494057445	LCGIAVIYTMFYVARRVFSRAQ	AEH	EANALLYLFEKLVMVIVW
	Burkholderia sp. RPE64	507515116	--------------------GE	---	-------------TA---
	Burkholderia sp. YI23	377819991	----------L---------G-	---	-G-----------T----
	Burkholderia sp. SJ98	495626609	----T-----L---------G-	T--	-------------T----
Other *Burkholderia*	Burkholderia sp. JPY347	517254370	-F---L--IA----------GE		Q-H-W-FIV----T-L--
	Burkholderia sp. TJI49	325520800	-F--GL--VL-FF-----A-DG		HTH-W-SIV--I-S----
	Burkholderia oklahomensis	497787915	-F--GL--IL-F------A-DG		HTH-W-SIV--I-SIV--
	Burkholderia multivorans	493443129	-F--GL--VL-FF-----A-DG		HSH-W-SIV----S----
	Burkholderia pseudomallei	497622429	-F--GL--IL-F------A-DG		HTH-W-SIV----SIV--
	Burkholderia mallei ATCC 23344	53726233	-F--GL--IL-F------A-DG		HTH-W-SIV----SIV--
	Burkholderia sp. CCGE1001	323527122	-F--GL--IV-FF------QDG		-THPW-F-V--I-SLV--
	Burkholderia thailandensis E26	83718952	-F--GL--IL-F------A-DG		HTH-W-SIV--F-SIV--
	Burkholderia phytofirmans PsJN	187925129	-F--GL--IV-FL------HDG		-THP--F-V--I-SLV--
	Burkholderia sp. H160	496199723	-V--GL-FIV-FL-----N-DG		GEHPW-F-V--A-S-V--
	Burkholderia cepacia GG4	402565719	-F--GL--VL-FF-----A-DG		HTH-W-SIV--I-S----
	Burkholderia dolosa	493816017	-F--GL--VL-FF-----A-DG		HTH-W-SIV--I-S----
	Burkholderia terrae	494860137	-F--GL--IV--L-----G-DD		S-HTW-S-V--V-SL---
	Burkholderia phymatum STM815	186475345	-F--GL--IV--L-----G-DG		S-HTW-S-V--V-SL---
	Burkholderia sp. BT03	495008588	-F--GL--IV--L-----G-DD		S-HTW-S-V--V-SL---
	Burkholderia ubonensis	497776754	-I--GL--VL-FF-----A-DG		STH-W-SIV--I-STV--
	Burkholderia sp. CCGE1003	307730759	-F--GL--IV-FF------QDG		-THPW-F-V--I-SLL--
	Burkholderia gladioli BSR3	330818068	-F---L--LL-S----A-A-DG		DTH-W-SII----S-L--
	Burkholderia ambifaria	493812336	-F--GL--VL-FF-----A-GG		HTH-W-SIV--I-STV--
	Burkholderia rhizoxinica HKI 4	312795302	-F---L--VAL-LM-----QDG		HLHG-A--V--I-T-L--
	Burkholderia sp. Ch1-1	494325051	-F--GL--IV-FF------HDG		ATHPW-F-V--I-SLV--
	Burkholderia kururiensis	516387122	-F--GL--IA-F------G-DG		-THVW-GIV--V-SLV--
	Burkholderia xenovorans LB400	91784969	-F--GL--IV-FF------HDG		ATHPW-F-V--I-SLV--
	Burkholderia cenocepacia MCO-3	170733869	-F--GL--VL-FF-----A-DG		HTH-W-SIV--I-STV--
	Burkholderia phenoliruptrix BR	407714527	-F--GL--IV-FF------QDG		-THPW-F-V--I-SLV--
	Burkholderia vietnamiensis	544730974	-F--GL--VL-FF-----A-DG		HTH-W-SIV--I-STV--
	Burkholderia lata	78067309	-F--GL--VL-FF-----A-DG		HTH-W-SIV--I-STV--
	Burkholderia sp. KJ006	387903025	-F--GL--VL-FF-----A-DG		HTH-W-SIV--I-SSV--
	Burkholderia graminis	492931277	-F--GL--IV-FF------HDG		-THPW-FVV--I-SLV--
	Burkholderia bryophila	518913239	-F--GL--IV-FF------HDG		-THPW-F-V--I-SLG--
Other Bacteria	Pandoraea sp. SD6-2	498506444	-F-T-L--LA------L-ASSP		Q-YG--RV--RVLTTFA-
	Ralstonia syzygii R24	344172153	-L-L-ML-LV---L---L-ANG		DLHGM-M-V-RVLTTLM-
	Ralstonia sp. PBA	497209044	-F---FL-GV---L---L-ANG		KLSHM-GFL--V-TTL–

Supplemental Figure 28: Partial sequence alignment of a conserved region of mechanosensitive ion channel protein MscS showing a 3 amino acid insertion (boxed) that is uniquely shared by the Clade IIa species.

Group	Species	Accession	199	Insertion	253
Clade IIa *Burkholderia*	Burkholderia sp. YI23	377820057	HHEATVQRLDTPFGSVTAAAKLDG	G	ARPFPLSGDLGYAGKVAGEAVNVSARLSGS
	Candidatus Burkholderia kirkii	494057777	---V--------------S--F--	-	---------A------D-----------A
	Burkholderia sp. RPE64	507515198	--------------A---SM----	-	---------A------G--T---G------
	Burkholderia sp. SJ98	495626483	----A-E-------A---S-----	-	---------A------NN-T----------
Other *Burkholderia*	Burkholderia phytofirmans PsJN	187923015	----AIE-------A---------		V--------I--S---ND---Q-GGH----
	Burkholderia xenovorans LB400	91782347	----AIE-------A-----R---		V--------I--S---NN---Q-GGH----
	Burkholderia sp. Ch1-1	494313286	----AIE-------A-----R---		V--------I--S---NN---Q-GGH----
	Burkholderia sp. BT03	495012230	----S-D-------A---------		V-----T--A--S---ND---Q-R-H----
	Burkholderia terrae	494862495	----S-D-------A---------		V-----T--A--S---ND---Q-R-H----
	Burkholderia bryophila	518909307	----A-E-------A---S-----		V-----T--V--S---NE---Q-GGH---T
	Burkholderia sp. WSM4176	517230891	----AID-------AL--------		V-------EV-----IND---Q-AGH---T
	Burkholderia graminis	492927605	----A-E-------G---S-----		VK----T--V--S---ND---Q-GGH----
	Burkholderia phenoliruptrix BR	407712553	----A-E-------A---S-----		VK----T--V--S---RD-P-Q-GGH----
	Burkholderia sp. CCGE1001	323525135	----A-E-------A---S-----		VK----T--V--S---RD-P-Q-GGH----
	Burkholderia phymatum STM815	186476944	----S-D-------A---V-----		M---S-T--A-----LND---Q-R-H----
	Burkholderia sp. CCGE1003	307728926	----A-E-------A---S-----		VK--A-A---S-S---RD---Q-GGQI---
	Burkholderia sp. CCGE1002	295675815	----AID--E----AL--------		VQ----T-EV--S---ND---Q-AGH---T
	Burkholderia sp. JPY251	517242871	----AID--E----AL--------		VQ----T-EV--S---ND---Q-AGH---T
	Burkholderia ubonensis	497776824	---LALE-----Y-AL--R-----		V---A-T--AT----L-D-P-DAR--V---
	Burkholderia pseudomallei	497622512	R-D--LE-------AL--N-R---		V---AID-SAT----LS--S-DAR--V---
	Burkholderia kururiensis	516383149	----S-E-----Y-A---H-T---		V---A-A--A--S---ND-D-Q-R-H-T-T
	Burkholderia oklahomensis	497788037	R-D---E-------AL--N-R---		V---AID--AT----LS--P-DAR--V---
	Burkholderia thailandensis	497597398	R-D--LE-------AL--N-R---		V---AID-SAT----LS--S-DAR--V---
	Burkholderia gladioli BSR3	330817989	--AL-LE-------AL--DVR---		----AIA-TA--S---SN-Q-D-H-T----
	Burkholderia mallei ATCC 23344	53725217	R-D--LE-------AL--N-R---		V---AID-SAT----LS--S-DAR--V---
	Burkholderia pyrrocinia	515903293	--DLVLEHV-----ALS-R-----		V---A-T--AT----L-D-P-DAR--V---
	Burkholderia dolosa	493816081	---IVLDNV---Y-AL--R-----		VK--A-T--AT----L-D-P-DAR--V---
	Burkholderia multivorans	493458823	---LALDSI---Y--L--R-----		MK--A-T--AT----F-D-P-DAR--V---
	Burkholderia ambifaria MC40-6	172061395	---LALDGV---Y--L--R-----		VK--A-T-NAT-V--L-D----A--NV---
	Burkholderia sp. JPY347	517254288	--ALSLDGI---Y-A---ELS---		V---A-A--V----N-DK---RA------T
	Burkholderia vietnamiensis	544735072	---LVLDGV---Y-AL--R-----		VK--A-T-EAT----LSD-P-DAR--V---
	Burkholderia cenocepacia	493543317	---LVLDGV---Y-AL--R-----		VK--A-T-EAT----LSD---DAR--V---
	Burkholderia cepacia GG4	402565788	---LALDGV---Y-AL--R-----		VK--A-T-NAT-V--L-N-P--A--NV---
	Burkholderia lata	78067238	---LVLDGV---Y-AL--R-----		VK--A-T-EAT----LSD-P-DAR--V---
	Burkholderia glumae BGR1	238028343	--TLSLE-------ALS-N-R---		----AID-AAS-T-NLSNQQ-DAH------
	Burkholderia sp. KJ006	387902962	---LALDGV---Y-AL--R-----		VK--A-T-TAT----LSD-P--A--NV---
	Burkholderia rhizoxinica HKI 4	312795360	R-HLVLD--R--Y--LH-DVA---		V---AVR--A-LS-QFD-QEMRLGVQ----
Other Bacteria	Cupriavidus basilensis	493147398	R-RVS-E--V--Y-KLA-N-QVA-		QA--A-N-AALLE-SWQK-SFSI--NVR--
	Herbaspirillum sp. JC206	517970759	Q-RIDLDNAV--Y-TLR-SLR-N-		K---AT--SVSMN-PIRD-SYRAA-Q----
	Ralstonia sp. PBA	497208896	R-RIA-DH-L----QT-LHGQ-E-		T---A-QAAATLDSTLK--QYQIA--A---
	Neisseria sp. GT4A_CT1	496669967	E-RLDLKT----WSNS-GS-VVGL		EK--A-NTTIQTK-ELE--TIEGK---W--
	Thauera phenylacetica	490526021	--RLESLAVEL-Q-R-R--LA---		VA--V-A-EA-FD-I-ETH--A-Q-T-AD-

Supplemental Figure 29: Partial sequence alignment of a conserved region of hypothetical protein BYI23_A006130 showing a 1 amino acid insertion (boxed) that is uniquely shared by the Clade IIa species.

			37		79
Clade IIa *Burkholderia*	Candidatus Burkholderia kirkii	494056428	LIDIAPVADDAPLRAALDEL	FLPPDAP	DRYALVVFVSPNAIDH
	Burkholderia sp. YI23	377820074	------I-------------	Y------	G---------------
	Burkholderia sp. RPE64	507515219	--------------------	-------	A---------------
	Burkholderia sp. SJ98	495626419	--------------------	----E--	G---------------
Other *Burkholderia*	Burkholderia sp. CCGE1001	323525183	-----------------GS-		E--S------------
	Burkholderia phenoliruptrix BR	407712597	-----------------GS-		E---------------
	Burkholderia graminis	492927492	-------E---------GS-		E---------------
	Burkholderia bryophila	518909351	-----------------AS-		E-----I---------
	Burkholderia sp. CCGE1003	307728970	----------T------GS-		E---------------
	Burkholderia sp. Ch1-1	494313115	---V-----E-------SS-		E------------V-Y
	Burkholderia phytofirmans PsJN	187923071	-------I---------AS-		E------------V--
	Burkholderia xenovorans LB400	91782421	---V---T-E-------SS-		E------------V-Y
	Burkholderia sp. JPY251	517242833	-------I-----C---AS-		E------------V--
	Burkholderia sp. CCGE1002	295675859	-------I-----C---AS-		E------------V--
	Burkholderia sp. WSM4176	517230937	-------I---S-C---AS-		E------------V--
	Burkholderia sp. H160	496198394	-------V-----CE-FAS-		E------------V--
	Burkholderia kururiensis	516383164	--A-G-AE-E----E-FAS-		-------------V--
	Burkholderia thailandensis MSM	488602117	------L------AE-FAR-		-A--------------
	Burkholderia pseudomallei	497622535	------L------AE-FAR-		-A-----------V--
	Burkholderia mallei ATCC 23344	53725179	------L------AE-FAR-		-A-----------V--
	Burkholderia oklahomensis	497800217	------L------AD-FAR-		-A--------------
	Burkholderia sp. BT03	495012267	-----AAD-E-----S-AS-		-------------V-Y
	Burkholderia gladioli BSR3	330817975	---------P---D--FAR-		-A-------------R
	Burkholderia ambifaria	493811788	-------D-P---D--FAT-		------I--------R
	Burkholderia phymatum STM815	186476929	--E--ATD-AT------AS-		EK-----------V-Y
	Burkholderia glumae BGR1	238028331	------AT-FG--D--FAR-		-A-------------R
	Burkholderia rhizoxinica HKI 4	312795371	----TDAT-N---VDG-LD-		E----A-------V-R
	Burkholderia sp. KJ006	387902948	------LD-P---V--FAT-		AD----I--------R
	Burkholderia vietnamiensis G4	134296606	------LD-P---V--FAT-		AD----I--------R
Other Bacteria	Ralstonia sp. PBA	497208863	-LE-R----S---A---SD-		S------------VT-
	Massilia niastensis	522143708	-LE-S----P-------A--		SD----A--------A
	Herbaspirillum lusitanum	515272800	-LE------STE--RV-SV-		E--------------A
	Cupriavidus necator N-1	339326970	-LA-G-A-----------AR-		-TF-----------TY
	Janthinobacterium sp. Marseill	152981768	-LE-L-LD-QQS--Q--R--		-Q---IA--------A

Supplemental Figure 30: Partial sequence alignment of a conserved region of Uroporphyrinogen-III synthase /Homolog of E. coli HemX protein showing a 7 amino acid insertion (boxed) that is uniquely shared by the Clade IIa species.

Group	Species	Accession	5		50
Clade IIb *Burkholderia*	Burkholderia phytofirmans PsJN	187923943	ELSNDEWALLAALVSDEP	A	VRLNRRGRPRAEPRIVANAVLWILTTG
	Burkholderia sprentiae WSM5005	548699263	---------V-P------	-	----------------T----------
	Burkholderia phenoliruptrix BR3459a	407711772	----------S-------	-	-----------D---------------
	Burkholderia sp. URHA0054	522812216	----------S-------	-	-----------D--V------------
	Burkholderia sp. WSM3556	548691598	------------------	-	-----------D--V------------
	Burkholderia sp. WSM2230	548606727	------------------	-	-----------D--V------------
	Burkholderia sp. WSM2232	548691232	------------------	-	-----------D--V------------
	Burkholderia xenovorans LB400	91783566	------------------	-	---------------------------
	Burkholderia sp. Ch1-1	385209645	------------------	-	---------------------------
	Burkholderia sp. CCGE1003	307729702	------------------	-	-----------D--V------------
	Burkholderia graminis C4D1M	170692457	----------S-------	-	-----------D--V------------
	Burkholderia sp. CCGE1001	323526003	----------S-------	-	-----------D--V------------
	Burkholderia mimosarum LMG 23256	548694969	---D---MQVSS------	P	I-------------V------------
	Burkholderia terrae BS001	390571745	--------QVS-------	-	---------------------------
	Burkholderia phymatum STM815	186475820	--------QVS-------	-	---------------------------
	Burkholderia sp. CCGE1002	295676560	--N--------P------	-	----------------T------M---
	Burkholderia sp. BT03	420247716	--------QVS-------	-	----------------V----------
	Burkholderia sp. H160	209517012	--N-----V--P------	-	I---------------T----------
	Burkholderia sp. WSM4176	517229217	--N--------P------	-	----------------T----------
	Burkholderia bryophila	518910075	----------S-------	-	-----------D--V------------
	Burkholderia sp. JPY251	517243162	--N--------P------	-	----------------T------M---
	Burkholderia kururiensis	516383878	----E---QV--------	V	--------------V------------
Other *Burkholderia*	Burkholderia cenocepacia J2315	206562710	--NDE--FR-S--IA---		I-------------V------------
	Burkholderia ambifaria IOP40-1	170700255	--NDE--VR-ST-IA---		I-------------V------------
	Burkholderia ubonensis Bu	167583852	--NDE--VR-ST-IA---		I-------------V------------
	Burkholderia sp. KJ006	387904652	--TDE--FR-ST-IA---		I-------------V------------
	Burkholderia vietnamiensis G4	134292908	--TDE--FR-ST-IA---		I-------------V------------
	Burkholderia cepacia GG4	402569668	--NDE--FR-ST-IA---		I-------------V------------
	Burkholderia sp. 383	78063125	--NDE--FR-ST-IA---		I-------------V------------
	Burkholderia multivorans CGD2M	221197125	--NDE--FR-SP-IA---		I-------------V------------
	Burkholderia sp. TJI49	416976992	--NDE--FR-SP-IA---		I-------------V------------
	Burkholderia dolosa AU0158	254253552	--TDE--FR-SP-IA---		I-------------V------------
	Burkholderia thailandensis TXD	167577089	--NDE---R-ST-IA---		I-----------N-V------------
	Burkholderia oklahomensis C678	167573672	--NDE---R-ST-IA---		I-----------N-V------------
	Burkholderia pseudomallei 406e	254301118	--NDE---R-ST-IA---		I-----------N-V------------
	Burkholderia mallei ATCC 23344	53716692	--NDE---R-ST-IA---		I-----------N-V------------
	Burkholderia glumae BGR1	238024998	---DE--TRVST-IA---		I-----------T-V----I-------
	Burkholderia gladioli BSR3	330821273	---DE--TRVST-IA---		I-----------T-V----I-------
	Burkholderia rhizoxinica HKI 4	312796176	D-KDH----VE--FCE--		A-VE------V---A-V-----V-A--
	Burkholderia sp. SJ98	413962416	D-RDN----VE--FCS--		A-SE------V-A-A-V-----V-S--
	Burkholderia sp. YI23	377821081	D-RDN----VE--FCA--		A-SE------V-A-A-V-----V-S--
	Candidatus Burkholderia kirkii	350545884	D-RDN----VE--FCA--		A-SEQ-----V-A-S-V-----V-S—

Supplemental Figure 31: Partial sequence alignment of a conserved region of transposase A-like protein showing a 1 amino acid insertion (boxed) that is uniquely shared by the Clade IIb species.

Group	Species	ID	153	Insertion (boxed)	194
Clade IIb *Burkholderia*	Burkholderia phymatum STM815	186475830	ITAVSQKVAAEIAAIGLT	P	RNRLDVIYNGVDTQGFAAAEGDR
	Burkholderia sp. BT03	420247706	---------T--------	-	-------------------Q---
	Burkholderia terrae BS001	390571755	---------T--------	-	-------------------Q---
	Burkholderia sp. WSM2232	548691232	---------D--R-----	A	E--V---------R-----T---
	Burkholderia sp. JPY251	517243153	---------D--R-----	-	---V---------------S---
	Burkholderia sp. WSM3556	548691598	---------D--R-----	-	D--V---------R--S--S---
	Burkholderia sp. URHA0054	522812216	---------D--R-----	-	D--V---------------T---
	Burkholderia bryophila	518910085	---------D--R-----	-	D--V---------------T---
	Burkholderia sprentiae WSM5005	548699263	---------D--R-----	-	---VE-------I------S---
	Burkholderia sp. WSM4176	517229207	---------D--R-----	-	---V---------------S---
	Burkholderia phenoliruptrix BR3459a	407711772	---------D--R-----	-	D--V---------R-----T---
	Burkholderia sp. WSM2230	548606727	---------D--R-----	-	D--V---------R-----T---
	Burkholderia sp. CCGE1002	295676570	---------D--R-----	-	---V---------------S---
	Burkholderia sp. H160	209517002	---------D--R-----	-	---V---------------S---
	Burkholderia graminis C4D1M	170692466	---------D--R-----	-	D--V---------------T---
	Burkholderia sp. Ch1-1	385209635	---------D--R-----	-	D--V---------------T---
	Burkholderia xenovorans LB400	91783577	---------D--R-----	-	D--V---------------T---
	Burkholderia sp. CCGE1003	307729712	---------D--R-----	-	Q--V---------R-----T---
	Burkholderia sp. CCGE1001	323525993	---------D--R-----	-	D--V---------R-----T---
	Burkholderia phytofirmans PsJN	187923953	---------D--R-----	S	D--V---------------T---
	Burkholderia kururiensis	516383888	-----K---D--RS----	-	S------------------S---
	Burkholderia mimosarum LMG 23256	548694969	-A---R---D--R-C-GH	-	VE-----------R--S--SA--
Other *Burkholderia*	Burkholderia sp. RPE64	507519372	VA---E---E--R-G-IE		KD-VE-V------TA-S--AP--
	Burkholderia multivorans ATCC	161521447	---------D----L-ID		-SKIG-------GSA--G-QA--
	Burkholderia sp. 383	78063115	---------D---GL-ID		SRKIS-------ASA--G-QA--
	Burkholderia oklahomensis EO14	167566579	---------D--ER--ID		GD-IG--------RA--N--P--
	Burkholderia sp. TJI49	416911476	---------D---GL-ID		SRKIS-------GSA--G-QA--
	Burkholderia cenocepacia J2315	206562719	---------D---GL-ID		SRKIS-------GSA--G-QA--
	Burkholderia ambifaria AMMD	115360297	---------D---GL-ID		SRKIS-------GSA--G-QP--
	Burkholderia sp. KJ006	387904663	---------D---GL-ID		SRKIS-------AGA--H-QA--
	Burkholderia vietnamiensis G4	134292918	---------D---GL-ID		SRKIS-------AGA--H-QA--
	Burkholderia cepacia GG4	402569658	--G------D---GL-ID		SRKIS-------GSA--G-QA--
	Burkholderia thailandensis MSM	167840472	---------D--RQ--ID		GE-IG-------A-A--N-AP--
	Burkholderia pseudomallei 1655	254184849	---------D--RR--ID		GG-IG-------A-A--N-AP--
	Burkholderia dolosa AUO158	254253562	---------D--RGL-ID		GGKIS-------GSA--G-QA--
	Burkholderia glumae BGR1	238024988	-V---G---D--RR--ID		GS-VG--------RA--G-RA--
	Burkholderia ubonensis Bu	167583863	---------D--RGL-ID		GGKIS-------AGA--D-QP--
	Burkholderia gladioli BSR3	330821263	VI---G---D--RRL-ID		G--IG-------A-A-GG-QP—

Supplemental Figure 32: Partial sequence alignment of a conserved region of group 1 glycosyl transferase showing a 1 amino acid insertion (boxed) that is uniquely shared by the Clade IIb species.

			145		219
Clade IIb *Burkholderia*	Burkholderia sp. H160	496202984	KYGVDRYVRFNAKVSAARFDEARQIWLVELDTN	GAR	EIIEADVLIAASGPLSRPALPRIAGIERFEGKLFHSA
	Burkholderia sp. CCGE1002	295676766	---------C----N---------V--I-----	---	-----------N-------------------------
	Burkholderia xenovorans LB400	91783855	----E----C--R---------H-L-R----V-	-V-	-S-----V-----------M-----LD----------
	Burkholderia sp. Ch1-1	494327703	----E----C--R-A---------L-R----V-	-V-	-S-----V-----------M-----LD----R-----
	Burkholderia graminis	492936566	---------C--R-V--------HV-QI---AG	-M-	-S-----------------M-----L-N-K-------
	Burkholderia sp. CCGE1003	307729928	----E----C--R-V--------YV-AI---AH	-T-	-T-----------G-----M-----------------
	Burkholderia phytofirmans PsJN	187924282	--D-E----C--R-A-S-------V-H-DV-V-	-V-	-T-----V-----------M-Q---L-----------
	Burkholderia sp. CCGE1001	323525825	----EA---C-TR-V--------HV-Q-D--AG	---	-H-------G---------M---P-LDS-K-------
	Burkholderia phymatum STM815	186476032	--Q--SAIQ---R-T--------LV-QLD-AR-	-MH	-TV---IV-----------M---D--GS-S-RI----
	Burkholderia sp. BT03	495008604	--Q--SAMQ---R-M--------LV-RLDIAR-	-VH	-TV---AV-S-I-L-----I---D-LDK-G--I----
	Burkholderia terrae	494862209	--Q--SAMQ---R-M--------LV-RLDIAR-	-VH	-TV---AV-S-I-L-----I---D-LDK-G--I----
	Burkholderia sp. WSM2232	548691109	---------C--R-V------V-YV-NI---AH	-T-	-------------G-----M-----------------
	Burkholderia sp. WSM2230	548606735	---------C--R-V--------HV-NI---AG	-T-	-H-----------G-----M------D----------
	Burkholderia sp. WSM3556	548691174	---------C--R-L--------HV-HI---VD	-T-	-N-----------G----SM-----L-S-K-------
	Burkholderia sp. URHA0054	522812216	---------C--R-V--------HV-QI---AG	-M-	-N-----------------M-----L-N-K-------
	Burkholderia sprentiae WSM5005	548699263	---------C----------Q------------	---	-T---------N-------------------------
	Burkholderia mimosarum LMG 23256	548694927	--D-E-FIEYRSR-R----V--HG--Q--IERD	--L	RT----IV---N---------QVP--G--A-------
	Burkholderia bryophila	518914327	---------C--R-V--------HV-HID--VD	-M-	-NV------G---G----SM-Q---L-S-K-------
	Burkholderia phenoliruptrix BR3459a	407713185	----EA---C-TR-V--------HV-Q----AG	---	-H-------G---------M---P-LDS-K-------
	Burkholderia sp. WSM4176	517228895	---------C------V----------------	---	--V--------N-------------------------
	Burkholderia sp. JPY251	517246749	---------C----NT--------V-------H	---	-----------N-------------------------
Other *Burkholderia*	Burkholderia cepacia GG4	402569879	---LA--L--G-E-ER--Y----AL-E-T-ADG		TVLS-A--VSGT-Q-------DLR--DT-R-RA----
	Burkholderia thailandensis	497584847	-F-IE-HIK-G-E-ER-EY--S-SV-R-A-ADG		TLV-TAL-VT-T-Q----V--KL----T---RA----
	Burkholderia sp. 383	78063361	---LA--L--G-E-AH--Y---AAL-R-T-ADG		TTLS-A--VSST-Q-----M-DLP--DT-R-RA----
	Burkholderia oklahomensis	497808405	--DLM-H--LG-E-AS-EY--R-SL-R-A-TDG		T-VATTL-VT-T-Q----I--KLP---T-K-HA----
	Burkholderia ambifaria MC40-6	172062537	---LA-HL--G-E-ER-QY----AL-H-T-ADG		TTLS-A--VSGT-Q-----M-DLP--DT-R-RA----
	Burkholderia cenocepacia	493541781	---LA--L--G-E-EH--Y---GAL-H-T-RDG		TTLS-AL-VSGT-Q-----M-DLP--DT-R-RA----
	Burkholderia vietnamiensis G4	134291654	---LA-HL--G-E-EH--Y---GAL-R-A-ADG		TTLS-A---SGT-Q-------KLP--DT-R-RA----
	Burkholderia sp. KJ006	387906357	---LA-HL--G-E-EH--Y---GAL-R-A-ADG		TTLS-A---SGT-Q-------KLP--DT-R-RA----
	Burkholderia glumae BGR1	238023351	---L---L--H-E-TH--Y--PHAL-RLT-SDG		STLS-AL--SGT-Q-------NLP--DT-R-RA----
	Burkholderia gladioli BSR3	330821758	---LE-HL--R-E-TH--Y--S-AL-C-T-SDG		STLG-AQ--SGT-Q-------KLP-MNE-R-RA----
Other Bacteria	Cupriavidus basilensis	493139021	Q--LAPHLQ-GTE-TS-S--KDAGH-VITSSDG		AVLH-R--VT-C-Q-N---V-AVP--DA-Q--V----
	Pseudomonas sp. GM48	495264983	---LSK-I--G-E-VS-E----TSM-Q-IQKDG		TVLT-SL--SGT-Q-----F-KL--MDN-K-HI----
	Marinobacter santoriniensis	496224236	---LQGHI--DSE-TR-E--SSANV-HIT-SND		TRLR--I--T-T-Q-NQ--I-DLE-LD----T-----
	Alcanivorax hongdengensis	496209449	--DIY-HI---TE-AD--YRDDQGL-E-T-TNG		-TLTCRA--T-T-Q-NQ--Y-PLK--DD-H-PH----
	Hydrocarboniphaga effusa	494341098	--DLL-HL--G-E-HS--Y--TGNL-T-L-RDG		SELRSKL--SGV-Q-----T-------S---HG----
	Acinetobacter sp. NIPH 298	491338612	--QIRPH-Q--TEIAS-D---VQGL-QIQST-G		-KF--EF----V-Q-N---Y-K-K---H-K--A----

Supplemental Figure 33: Partial sequence alignment of a conserved region of 4-hydroxyacetophenone monooxygenase showing a 3 amino acid insertion (boxed) that is uniquely shared by the Clade IIb species.

Group	Species	Accession	208	(boxed)	275
Clade IIb *Burkholderia*	Burkholderia sp. H160	209521823	SLSEEPLICALIDEFRNDLVNIRFMPDVRSHALFEGS	G	VVDLLGVPAINLVASPLSANAMFKKEIFDR
	Burkholderia sp. CCGE1002	295675675	----------------D--------------------	-	------M---------------L-------
	Burkholderia sp. CCGE1001	323524889	--A------S-VG---D-------------L------	-	-I------------------S-L-------
	Burkholderia phytofirmans PsJN	187922822	---------S-VS---D-------------L------	-	-I------------------S-L-------
	Burkholderia sp. CCGE1003	307728617	--A------S-VG---D-------------L------	-	-IE----------------SS-L-------
	Burkholderia xenovorans LB400	91781978	---------S-VS---D-------------L------	-	-I-----------------SS-L-------
	Burkholderia graminis C4D1M	170691276	--A------S-VS---D-------------L------	-	-IE----------------SS-L-------
	Burkholderia sp. Ch1-1	385179022	---------S-VS---D-------------L------	-	-I-----------------SS-L-------
	Burkholderia phymatum STM815	186477223	--TD-RT-FRIVT-----------------L---DT-	S	-IE---------------SR-L---DL---
	Burkholderia terrae BS001	390567786	--TD-RT-FR-VT-----------------L---DT-	S	-IE---------------SR-L---DL---
	Burkholderia sp. BT03	398072227	--TD-RT-FR-VT-----------------L---DT-	S	-IE---------------SR-L---DL---
	Burkholderia sp. WSM2232	548691215	--AQ-----S-VG---D-------------L------	-	-IE----------------SS-L-------
	Burkholderia sp. WSM2230	548606715	--A------S-VG---D-------------L------	-	-IE----------------SS-L-------
	Burkholderia sp. URHA0054	522812206	--A------S-VS---D-------------L------	-	-IE----------------SS-L-------
	Burkholderia sprentiae WSM5005	548699347	----------------D--------------------	-	------M---------------L--D----
	Burkholderia sp. JPY251	517242987	----------------D--------------------	-	------M---------------L-------
	Burkholderia phenoliruptrix BR3459a	407712258	--A------S-VG---D-------------L------	-	-I------------------S-L-------
	Burkholderia sp. WSM4176	517230753	----------------D--------------------	-	----------------------L-------
	Burkholderia bryophila	518913906	---------S-VS---D-------------L------	-	-I------------------S-L-------
	Burkholderia mimosarum LMG 23256	548694975	--A--RTVVR-VN-----------I-----L---DT-	S	-IE--------------P-S-Q---AM---
Other *Burkholderia*	Burkholderia sp. WSM3556	548691524	-----RA-LE-V-------I-V-------AL---D--		MTE-----T---A----PP--LV---L---
	Burkholderia kururiensis	516386685	P----RM-ARVVN---D-------I---SDMT--DS-		MIE-I-------M----PLH-KL---L---
	Burkholderia sp. SJ98	413958678	P-----T-LRFVN---D--I-V--I-----L----SG		-T----Q-------T----S-QLQ------
	Burkholderia sp. YI23	377821492	P----RT-LRFVN---D----V--I-----L----SG		-T----Q---D--------S-QLQ--L---
	Burkholderia sp. KJ006	387906656	P----AT-LRFVNA-GD-------I-----V---D-E		M---M-A-----------PRH--W---L---
	Burkholderia vietnamiensis G4	134291910	P----AT-LRFVNA-GD-------I-----V---D-E		M---M-A---------MPRH--W---L---
	Burkholderia sp. TJI49	416926494	P----AT-LRFVN--GDE------I-----V---D-A		MI--V-A-----------RH--IQ------
	Burkholderia ambifaria MC40-6	172062723	P----RT-LRFVS---DE----------SALS--DSA		LT-V--------A----PPH-LLS------
	Burkholderia mallei SAVP1	121597372	P-----Q-HRIVT-----F-----V-----LSF-NQE		-IE---------A---ITDVRILP-FV---
	Burkholderia rhizoxinica HKI 4	312602276	P--H-RV-QRIVR---H-F--L--L-----MT--SQ-		-TEV--M-----A---V-DPQLWP-RV---
	Burkholderia pseudomallei 1106	126456541	P-----Q-HRIVT-----F-----V-----LSF-NQE		-IE---------A---ITDVRILP-FV---
	Burkholderia thailandensis Bt4	167615262	P-----Q-HRIVT-----F-----V-----LSF-NQE		-IE---------A---ITDVRILP-FV---
	Burkholderia ubonensis Bu	167583854	P-----Q-NRIVTV--H-F-----I----AVSF-NQE		--E---------A---ITDVRILP-FV---
	Burkholderia cenocepacia J2315	206562080	P-A-DAS-TRV--K--S-F-EL-L----SQ----GSQ		-DEI--E---S-A-P---RG-LCV-AV---
	Burkholderia dolosa AU0158	254253554	PI---RR-HQIVTV--D-F-----I-----LSF-NQE		--EV--------A---ITDVRILP-FV---
	Burkholderia cepacia GG4	402569666	PIT--RR-HQIVTV--H-F-----I----TLSF-NQE		--E---------A---ITDVRILP-FV---
	Burkholderia sp. 383	78063710	PIG-ARIVM-VL-A--D-----------SKL-M-D-E		MI--V-A-----------SR-LLH-A----
	Burkholderia multivorans ATCC	189352376	PI---RR-HQIVTV--H-F-----I-----LSF-NQE		--EV--------A---ITDVRILP-FV---
	Burkholderia oklahomensis EO14	167566587	P-----Q-HRIVT-----F-----V-----LSF-NQE		-IE---------A---ITDVRILP-FV---
	Burkholderia gladioli BSR3	330821271	PIT--RQ-HRIVT--QH-F----LI-----LSF-NQE		--E---------A---ITDVRILP-FV---
	Burkholderia glumae BGR1	238024996	PIT--RQ-HRIVK--QH-F----LI-----LSF-NQE		--E---------A---ITDVRILP-FV---
Other Bacteria	Cupriavidus necator HPC(L)	421750268	PMAACRDL-DVVYQ------D--WV--IM-VE-LGHR		FSEF--L-V-D-NSP-D-RITGLL-AS---
	Achromobacter piechaudii ATCC	293606521	PMEAGQELREVLYHL-----D--WI---M-IQ-LGHR		IGEF--L---Q-NSL-AAGVRGWA--V---

Supplemental Figure 34: Partial sequence alignment of a conserved region of undecaprenyl-phosphate glucose phosphotransferase showing a 1 amino acid insertion (boxed) that is uniquely shared by the Clade IIb species.

Group	Organism	Accession	102		148
Clade IIb *Burkholderia*	Burkholderia phymatum STM815	186476032	IQFNARVTAARFDEARLVWQLDLARN	GMH	ETVEADIVIAASGPLSRP
	Burkholderia sp. BT03	420248446	M------M-----------R--I---	-V-	-------A--S-I-L----
	Burkholderia terrae BS001	390573067	M------M-----------R--I---	-V-	-------A--S-I-L----
	Burkholderia graminis C4D1M	170695221	VRC----V---------H---IE-DAG	--R	-SI---VL----------
	Burkholderia phytofirmans PsJN	187924282	VRC----A-S-------Q--HV-VDV-	-VR	--I---V-----------
	Burkholderia sp. H160	209520814	VR---K-S---------QI-LVE-DT-	-AR	-II---VL----------
	Burkholderia sp. Ch1-1	385209290	VRC----A---------QL-RVE-DV-	-VR	-SI---V-----------
	Burkholderia xenovorans LB400	91783855	VRC----S-------HQL-RVE-DV-	-VR	-SI---V-----------
	Burkholderia sp. CCGE1001	323525825	VRC-T--V---------H---V--DAG	-AR	-HI---VL-G--------
	Burkholderia sp. CCGE1003	307729928	VRC----V---------Y--AIE-DAH	-TR	--I---VL-----G----
	Burkholderia sp. CCGE1002	295676766	VRC--K-N---------Q--LIE-DT-	-AR	-II---VL---N------
	Burkholderia bryophila	518914327	VRC----V---------H--HI--DVD	--R	-N----VL-G---G----
	Burkholderia sp. WSM2232	548691109	VRC----V------V-Y--NIE-DAH	-TR	-II---VL-----G----
	Burkholderia sp. WSM2230	548606735	VRC----V---------H--NIE-DAG	-TR	-HI---VL-----G----
	Burkholderia sp. WSM3556	548691174	VRC----L---------H--HIE-DVD	-TR	-NI---VL-----G----
	Burkholderia sp. URHA0054	522812216	VRC----V---------H---IE-DAG	--R	-NI---VL----------
	Burkholderia sprentiae WSM5005	548699263	VRC--K-S-----Q--QI-LVE-DT-	-AR	--I---VL---N------
	Burkholderia mimosarum LMG 23256	548694927	-EYRS--R----V--HGI--VEIE-D	-AL	R-I---------N------
	Burkholderia phenoliruptrix BR3459a	407713185	VRC-T--V---------H---VE-DAG	-AR	-HI---VL-G--------
	Burkholderia sp. JPY251	517246749	VRC--K-NT--------Q--LVE-DTH	-AR	-II---VL---N------
	Burkholderia sp. WSM4176	517228895	VRC--K-S-V-------QI-LVE-DT-	-AR	-I----VL---N------
Other *Burkholderia*	Burkholderia glumae BGR1	238023351	LR-H-E--H--Y--PHAL-R-T-SDG		S-LS-ALL-SGT-Q----
	Burkholderia thailandensis TXD	167577045	-K-G-E-ER-EY--S-S--RVA--DG		TL--TALLVT-T-Q----
	Burkholderia cepacia GG4	402569879	LR-G-E-ER--Y----AL-EVT--DG		TVLS-AVLVSGT-Q----
	Burkholderia ambifaria AMMD	115360079	LR-G-E-ER-QY----AL-HVT--DG		T-LS-AVLVSGT-Q----
	Burkholderia gladioli BSR3	330821758	LR-R-E--H--Y--S-AL-CVT-SDG		S-LG-AQL-SGT-Q----
	Burkholderia sp. 383	78063361	LR-G-E-AH--Y---AAL-RVT--DG		T-LS-AVLVSST-Q----
	Burkholderia vietnamiensis G4	134291654	LR-G-E-EH--Y---GAL-RVA--DG		T-LS-AVL-SGT-Q----
	Burkholderia sp. KJ006	387906357	LR-G-E-EH--Y---GAL-RVA--DG		T-LS-AVL-SGT-Q----
	Burkholderia cenocepacia H111	421865838	LR-G-E-EH--Y---GAL-HVT-RDG		T-LS-ALLVSGT-Q----
Other Bacteria	Variovorax sp. CF313	398809804	---KT---R-VW----KL-TVTTD-G		D-Y--TYC-M-T----I-
	Cupriavidus basilensis OR16	374365435	-R-GTE--E--Y---ACL--VATSNG		D-IA-R-LVSGT-Q-N--
	Comamonas testosteroni KF-1	221070098	----S--KS-T---S-VR-TVETEAG		-KFD-QYL-M-T-C--I-
	Nitrosospira multiformis ATCC	82702497	-RL--AL-S----AEQGL-L-R-ENG		-QLK-SFF-CSA----E-
	Limnobacter sp. MED105	149925661	----TSI-GSV---QAGL-NITT-DG		K-IQ-RN-VT-V--F-A-
	Methylibium petroleiphilum PM1	124268099	--LET---S-H---KASR-TVTTN-G		---S-QFC-M-T-C--T-
	Acinetobacter nosocomialis Ab2	424055752	K--HE--H-QY--T-H--M--F-NQ		PSL--QF-VF-----HI-
	Alcanivorax dieselolei B5	407694497	-R--SE-R------K-AR-T-R-TDG		T--DCQHWVL-T---HV-
	Caulobacter crescentus CB15	16126061	---T--QTIA--D--R--T-TTDQG		QRF---NL-M-T----L-
	Novosphingobium aromaticivoran	146275933	-R--SE-VGLDW----HL-T-AT-DG		LRE-Y-V------V-HH-
	Tistrella mobilis KA081020-065	389877836	-R-GTA-E--A--DQ-GL-TI--VGG		GRM--EVL-S-V-Q-D–

Supplemental Figure 35: Partial sequence alignment of a conserved region of putative flavin-binding monooxygenase-like protein showing a 3 amino acid insertion (boxed) that is uniquely shared by the Clade IIb species and *Pseudomonas putida*.
